# AI-redesigned starting points and outcomes enhance protein evolution

**DOI:** 10.1038/s41586-026-10820-0

**Published:** 2026-07-22

**Authors:** Nicholas A. Krasnow, Joy A. Xu, Emily Zhang, Gandhar K. Mahadeshwar, Y. Allen Tao, Julia McCreary, Colin F. Hemez, Logan E. Brown, Wei Jiang, David R. Liu

**Affiliations:** 1 Merkin Institute of Transformative Technologies in Healthcare, Broad Institute of Harvard and MIT, Cambridge, MA USA; 2 Department of Chemistry and Chemical Biology, Harvard University, Cambridge, MA USA; 3 Howard Hughes Medical Institute, Harvard University, Cambridge, MA USA; 4 Center for the Development of Therapeutics, Broad Institute of Harvard and MIT, Cambridge, MA USA

**Keywords:** Molecular engineering, Protein design

## Abstract

Engineered or laboratory-evolved proteins often have suboptimal stability, activity or specificity. Here we applied artificial intelligence (AI)-based protein sequence design to address challenges in experimental enzyme evolution. Using the model ProteinMPNN, we redesigned three distinct botulinum neurotoxin (BoNT) proteases, generating variants with improved stability and full catalytic efficiency^1^. We hypothesized that redesigned enzymes may be more mutationally robust than their wild-type (WT) counterparts, and therefore may serve as better starting points to evolve new function. We performed side-by-side phage-assisted continuous evolution campaigns initiated with AI-redesigned proteases or with the corresponding WT proteases^2^. Evolving three distinct redesigned enzymes as starting points consistently yielded proteases with higher activity than evolving WT proteases in the same selection. Across four evolution campaigns, redesign conferred robustness that unlocked access to otherwise inaccessible highly functional sequences, confirmed by the inability of redesign-evolved mutations to function in WT enzyme backgrounds. When redesign raises fitness in sequence space local to the starting point, redesigned starting points adapt at a faster rate. Finally, we evolved both WT and AI-redesigned BoNT/E protease to selectively cleave the therapeutically relevant protein ataxin-2. Proteases evolved from the redesigned starting point reached higher catalytic efficiency and stability while minimizing native substrate cleavage, achieving more than 79-fold greater selected specificity for ataxin-2 than the best-performing variant evolved from WT BoNT/E. This study establishes a practical workflow using AI-redesigned starting points to evolve enzymes with improved properties compared with those evolved from natural proteins, with broad implications for protein science.

## Abstract (web summary)

A workflow using artificial intelligence-redesigned starting points to evolve enzymes with improved properties compared with those evolved from natural proteins is established.

## Main

Engineering enzymes for new or improved function provides solutions to problems in biomedical research, therapeutics, chemical synthesis and environmental science^3,4^. Enzymes with tailor-made properties can be generated through directed evolution, typically starting from a natural protein. Phage-assisted continuous evolution (PACE) is a particularly powerful protein evolution method owing to its ability to perform dozens of generations of mutation, selection and replication every 24 h without researcher intervention^2,5^. PACE selections have been used to reprogramme the specificity of diverse proteins, including DNA-binding domains, antibody mimetics, degron tags and catalysts including polymerases, aminoacyl-tRNA synthetases, dehydrogenases and proteases^2,6–12^. PACE has also yielded programmable nucleases, base editors, prime editors, transposases and recombinases with robust gene-editing activity in mammalian cells^7,13–17^. All of these examples initiated evolution with genes encoding natural proteins.

Although a natural enzyme can serve as an effective starting point for laboratory evolution, directed evolution experiments struggle when mutations that support the desired function incur excessive folding or solubility deficiencies^18–21^. Most WT proteins are marginally stable^22,23^, and, because most mutations are destabilizing, an evolving protein that accumulates mutations to access new properties will often lose stability^24–26^. The stability that a protein can draw from to accommodate mutations that increase desired function before losing the ability to fold can therefore determine its evolutionary potential^20,27^.

Trading stability for new function may help to explain why evolved enzymes often have lower activity or specificity for their new functions than their WT starting points. Although PACE and other evolution methods can markedly change substrate specificities, evolved catalytic potencies on non-native substrates are often lower those that of the WT enzymes on their native substrates^10,11,28,29^. Reprogrammed enzymes may also retain activity on undesired native substrates^10,11,13^. Observations of natural evolution as well as laboratory evolution experiments have revealed examples in which enzymes must first evolve higher stability before they can evolve activity on new substrates^20,30,31^. Proteins that evolve a desired function but lose stability in the process have required additional engineering to recover sufficient stability to be used effectively^21^.

One approach to increase the evolutionary potential of natural enzymes is to first evolve the enzyme for enhanced thermal stability or soluble expression, then use the stabilized protein as the starting point for evolving desired properties^32,33^. This workflow requires substantial additional time and labour to achieve experimental evolution for stability, in addition to evolving the desired activity. Furthermore, simultaneous selection to retain function in soluble expression evolution is challenging to enforce while mitigating cheating, in which protein expression uncouples from reporter expression and activation^32^. Researchers have also rescued degraded stability by overexpressing chaperones during evolution, but the resulting evolved genotypes might not express well in the absence of a chaperone^19^. We hypothesized that computational protein design might overcome the above limitations of natural proteins as starting points for directed evolution. Machine learning-based methods have progressed rapidly in the de novo design of enzymes^34–36^. De novo-designed enzymes, however, are usually outperformed in catalytic rate by their natural counterparts, and design methodology currently requires an active site from a natural enzyme or predicted by quantum mechanics^36^.

Machine learning models as well as physical modelling have successfully redesigned the sequences of natural proteins. Both the neural network-based ProteinMPNN and the physical energy-based method PROSS have improved protein stability while retaining native function for proteins with various folds and functions^1,37,38^. We sought to explore whether laboratory-evolved enzymes can be redesigned computationally to restore the stability lost during evolution, and to determine whether stability-redesigned enzymes serve as superior starting points for laboratory protein evolution. Encouragingly, a recent report has demonstrated that a sequence-redesigned Fe(II)/αKG-superfamily enzyme can evolve higher hydroxylase activity than a WT enzyme in discrete screening^39^. However, the evolvability of sequence-redesigned enzymes across distinct substrates with diverse starting activities and physicochemical properties has not yet been reported. More generally, how sequence redesign modifies the broad fitness landscape surveyed over many generations of laboratory evolution remains to be investigated. Whether a protein redesigned for native function can more readily evolve to eliminate that function in favour of a non-native one is also unexplored, despite the usefulness of such a capability to generate proteins with novel specificities.

**Fig. 1:**
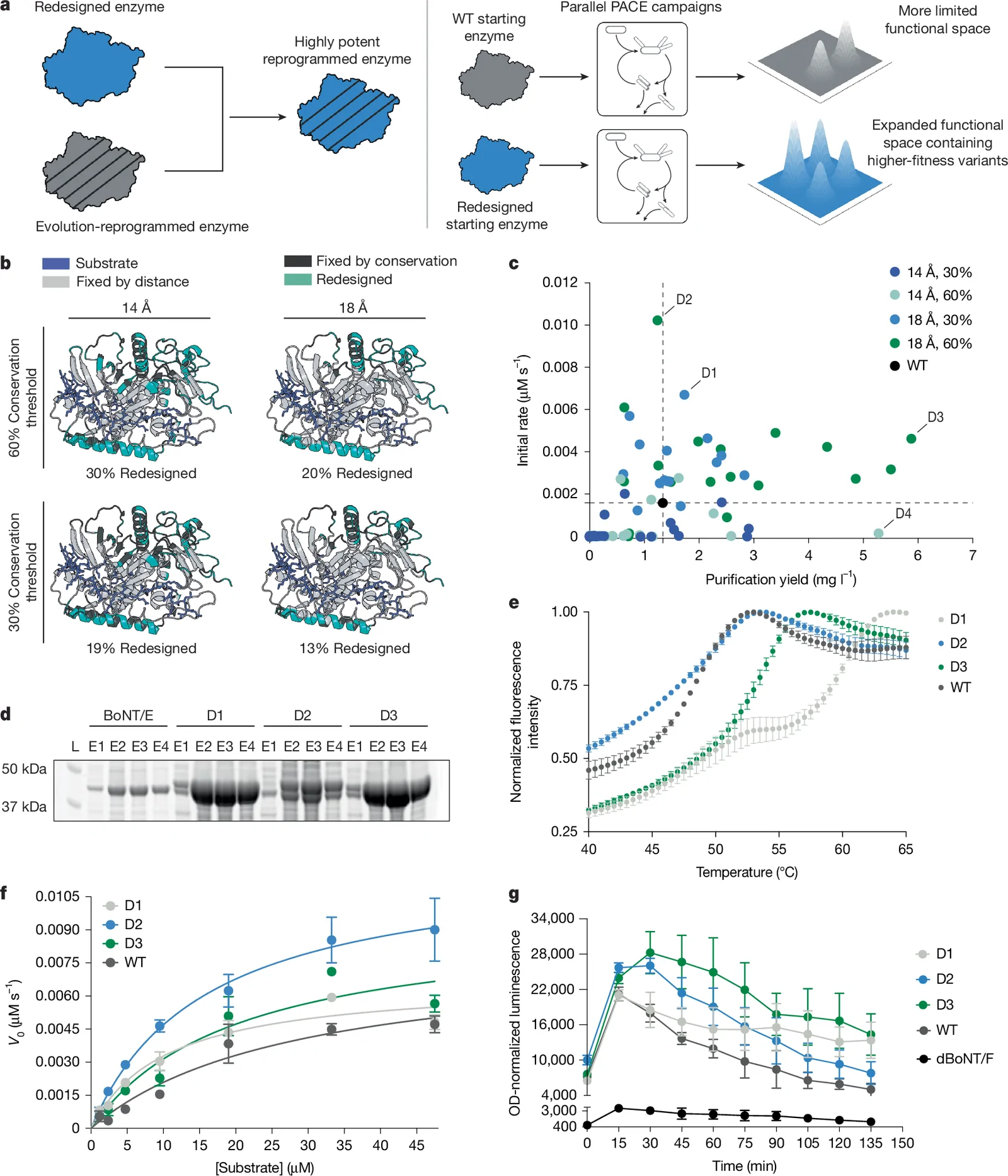
Overview for integrating computational protein sequence design and continuous evolution, and redesign of stabilized BoNT/E proteases. **a**, Left, redesigning laboratory-evolved enzymes yields highly potent enzymes with reprogrammed specificity. Right, redesigned proteases are superior evolutionary starting points that have an expanded, higher-fitness mutation space. **b**, Residues that were constrained or allowed to vary during redesign in BoNT/E protease. **c**, Catalytic rate and soluble yield for assayed designs. Rate assays were conducted with 2.5 nM protease and 4.75 µM SNAP25 FRET substrate. The dashed lines indicate values for the WT protease. **d**, Abundance of top redesigned proteases in purification elution fractions E1–E4 from the soluble fraction of *E. coli* expression cells. Out of approximately 1 ml of elution fraction, 5 µl was loaded into each lane. **e**, Protein thermal melting curves to measure *T*_m_. Thermal unfolding was followed by SYPRO orange fluorophore. *T*_m_ values, calculated as the temperature at which the slope of the curve is maximal, are provided in Supplementary Table 6. **f**, Michaelis–Menten plot for in vitro cleavage kinetics with the SNAP25 FRET substrate. Resulting kinetic parameters for BoNT/E protease variants are provided in Supplementary Table 5. *V*_0_, initial velocity. **g**, Apparent protease activity in PACE protease cleavage selection circuit host cells measured by luciferase signal. OD, optical density. The values and error bars represent mean ± s.d. of three replicates (**e**–**g**).

Here we report that integrating sequence redesign with laboratory protein evolution overcomes challenges inherent to each approach, using protease reprogramming as a model evolutionary challenge. We used ProteinMPNN to redesign reprogrammed proteases after laboratory evolution to restore stability, activity and soluble expression (Fig. 1a, left). To investigate evolvability, we performed replicate side-by-side evolution campaigns for three distinct substrates initiated with three stability-redesigned proteases or the WT protease, revealing that initiating protein evolution with ProteinMPNN-redesigned or PROSS-redesigned enzymes consistently led to higher activity than evolution of the WT starting enzymes (Fig. 1a, right). We found that redesigned starting points expand the functional sequence space of evolution, evolve new enzyme function faster and succeed at a higher rate than WT starting points. Finally, a redesigned protease evolved to specifically cleave the disease-relevant ataxin-2 protein achieved enhanced activity and stability compared with WT-evolved proteases evolved in parallel. The best-performing ataxin-2-cleaving proteases evolved from the redesigned protease starting point were far more specific for ataxin-2 than proteases evolved from the natural enzyme. Our results establish a workflow that integrates computational sequence design and laboratory evolution to generate enzymes with new properties more effectively.

## Protease redesign for stability

We chose proteases to investigate the evolution of computationally reprogrammed enzymes^10,11,40^. We have previously showed that protease PACE can generate enzymes that process substrates vastly different from the native substrate and show low activity on off-target substrates, resulting in specificity changes of more than 10^6^-fold^11^. During protease PACE, the fitness of phage encoding an evolving protease is coupled to the activity of that protease on a target substrate. An autoinhibited T7 RNA polymerase variant with the target substrate inserted between the inhibitory and polymerase domains as well as the essential phage gIII under control of the T7 promoter are encoded on an accessory plasmid in *Escherichia coli* host cells. Phage infection and the protease-mediated cleavage of the target substrate liberates T7 RNA polymerase to transcribe gIII, enabling propagation of phage encoding that active protease^40^. For concomitant negative selection against an off-target substrate, an orthogonal autoinhibited T7 variant is encoded with the off-target substrate such that off-target cleavage activates transcription of gIII-neg, a dominant negative variant that suppresses phage propagation^6^. A host cell mutagenesis plasmid^41^ continuously mutates phage genomes, and propagating phage are continuously diluted by fresh host cell culture so that phage encoding more active or specific proteases enrich over many generations of mutation, selection and replication.

We selected members of the BoNT proteases as candidates for computational redesign and evolution. We have previously used PACE to evolve BoNT proteases with new substrate specificities^11^. Natural BoNT proteases are a component of a holotoxin that self-delivers into human neurons, enabling their widespread use in cosmetics and therapeutics^42^. We began by redesigning BoNT proteases with ProteinMPNN to improve their stability and expression while retaining catalytic function. Guided by previous redesign efforts with ProteinMPNN^37^, we developed a procedure for the design, in silico assessment and experimental validation of BoNT protease variants. For each protease serotype, we identified the set of residues to constrain from design so that ProteinMPNN would not alter amino acids critical to substrate binding, Zn^2+^ binding or catalysis. We constrained the binding pocket as defined by all residues within a specified distance to the substrate in a crystal structure, and we constrained evolutionarily conserved residues that may be important for function. The remaining (unconstrained) residues were redesigned with ProteinMPNN using the WT structure of the BoNT protease as the input. Designs were assessed for folding to the target structure by predicting structures in AlphaFold2 (AF2)^43^, and evaluating model confidence (predicted local distance difference test) and predicted similarity to the WT structure (root mean square deviation).

**Extended Data Fig. 1:**
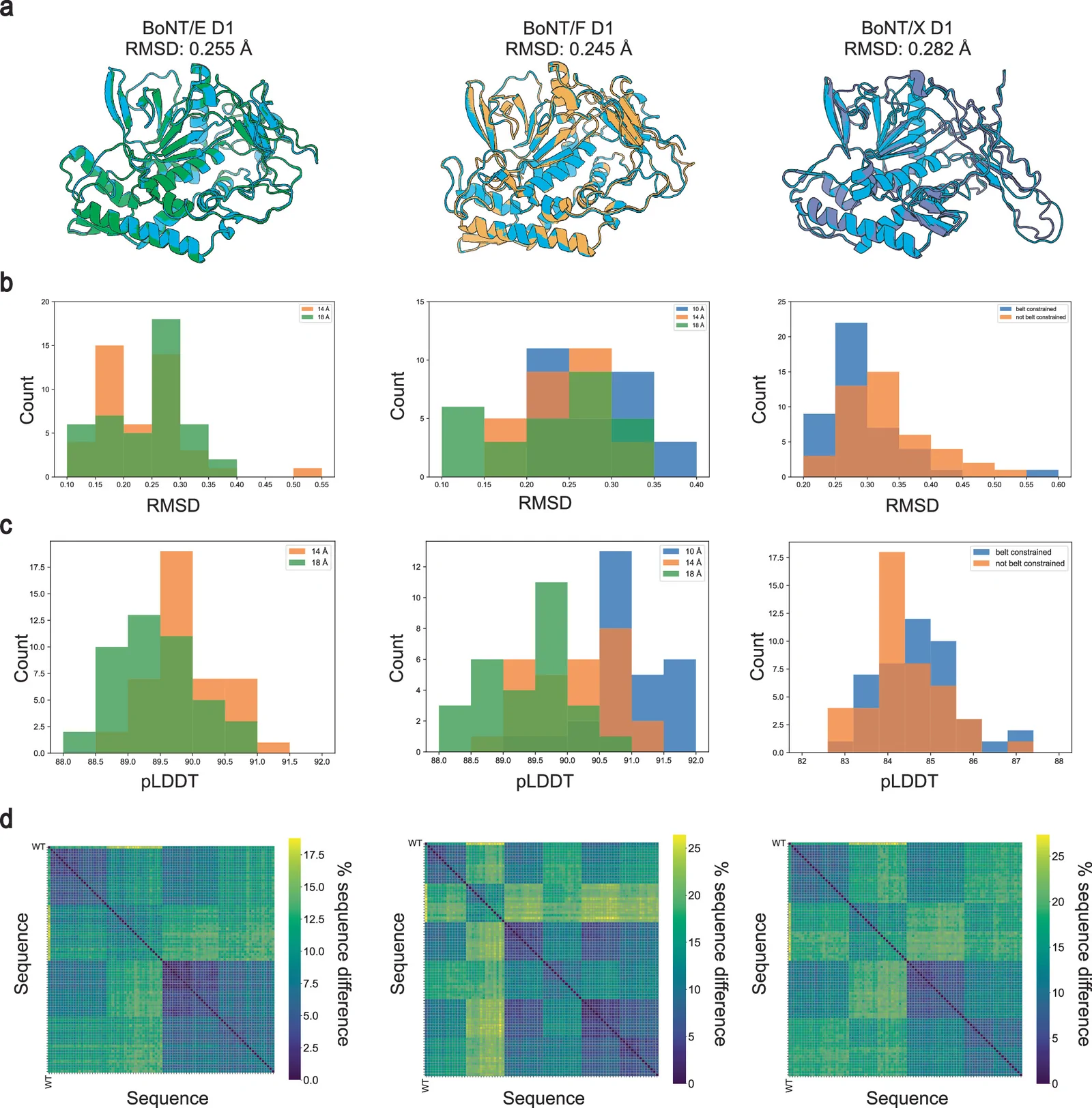
In silico characterization of ProteinMPNN-redesigned BoNT proteases. (**a**) The AlphaFold2 (AF2)-predicted structures of top-performing BoNT protease redesigned variants aligned to the predicted structure of the parental wild-type proteases (cyan) for BoNT/E (left, green), BoNT/F (middle, brown), and BoNT/X (right, purple) proteases. (**b**) Distributions of RMSD values between AF2-predicted redesigned protease and AF2-predicted wild-type protease structures grouped by redesign criteria. (**c**) Distributions of model confidence scores for AF2-predicted redesigned protease structures grouped by redesign criteria. (**d**) Pairwise percentage of residues differing between wild-type and redesigned protease sequences. RMSD, root mean square deviation; pLDDT, predicted local distance difference test.

First, we generated stabilized variants of WT BoNT/E protease. To find the combination of design constraints that best allow improvements to stability without perturbing catalysis, we generated ProteinMPNN designs with binding pocket thresholds of 14 Å or 18 Å. These stringent distance constraints, although heuristic, were set intending to preserve the structural dynamics in BoNT proteases^44^. Each residue closer than the threshold distance to the substrate or catalytic Zn^2+^ was constrained. We also applied two conservation cut-offs of 30% or 60%; each residue with at least the cut-off frequency in a multiple sequence alignment was not allowed to vary (Fig. 1b). We evaluated 22 designs for each combination of constraints with AF2 and confirmed that all predicted structures scored well (Extended Data Fig. 1). Structural features, property changes and mutation conservation of ProteinMPNN-redesigned mutations are provided in Supplementary Figs. 2 and 3. We expressed and purified proteases for 74 designs in plate-based arrayed purification. We measured soluble protease yields and cleavage rates of the native SNAP25 substrate alongside WT BoNT/E protease. Among the 74 designs, 58 (78%) were functional proteases, with 33 (45%) showing cleavage rates at least as high as WT BoNT/E protease. Among these designs, 22 (30%) both retained activity and expressed with higher soluble yield than the WT protease (Fig. 1c). Most active proteases, including those with the greatest improvement in catalysis or soluble yield, came from the 18 Å threshold design group, suggesting that redesign closer to the binding groove in the 14 Å group was usually deleterious.

We selected three of the highest expressing and most active designs for further characterization (D1–D3; Fig. 1c). All three of the top designs showed higher soluble expression in *E. coli* than in the WT protease (Fig. 1d and Supplementary Fig. 1), and enhanced thermal stability (Fig. 1e and Supplementary Table 5). Michaelis–Menten analysis revealed catalytic efficiencies (*k*_cat_/*K*_m_) of 260 mM^−1^ s^−1^, 310 mM^−1^ s^−1^ and 190 mM^−1^ s^−1^ for D1, D2 and D3, respectively, 1.7–2.8-fold higher than that of WT BoNT/E protease (110 mM^−1^ s^−1^; Fig. 1f and Supplementary Table 6).

We next sought to understand how improvements in soluble expression and catalytic rate determine fitness in the PACE selection circuit. We assessed cleavage of the T7 RNAP protease PACE substrate in *E. coli* via a luminescence assay in which target cleavage activates T7 polymerase to transcribe luciferase^6^. D2 and D3 produced higher maximal luciferase signal, and all three top designs mediated more durable cleavage, than WT BoNT/E protease (Fig. 1g), suggesting that sequence redesign improves starting fitness. Together, the experimental characterization of BoNT/E proteases demonstrates that ProteinMPNN design successfully produced variants with improved stability and activity.

**Extended Data Fig. 2:**
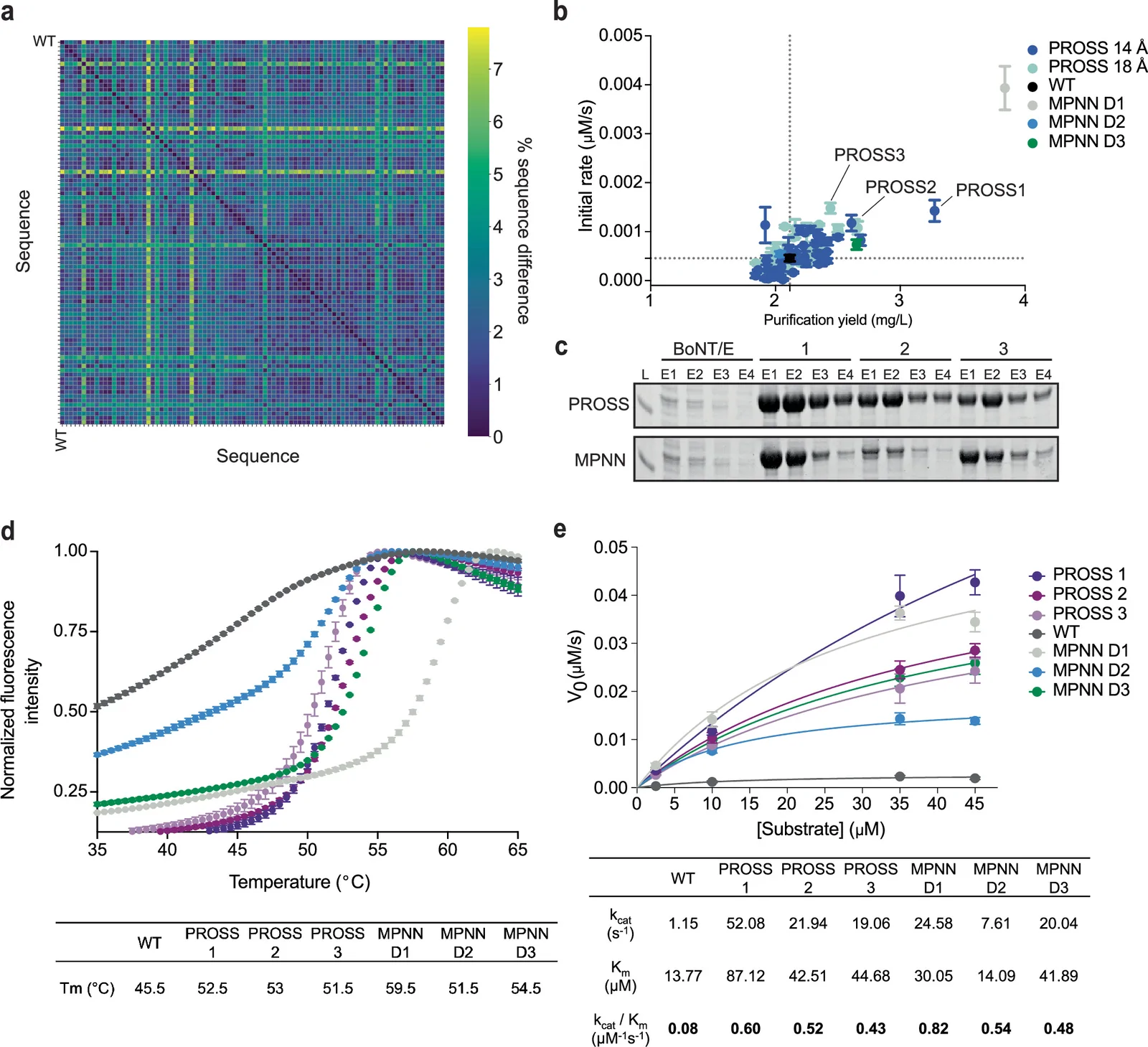
PROSS redesign of BoNT/E proteases and comparison to ProteinMPNN redesign. (**a**) Pairwise percentage of residues differing between wild-type and PROSS-redesigned protease sequences. (**b**) Catalytic rate and soluble yield for assayed PROSS designs in parallel with top ProteinMPNN redesigns. Rate assays were conducted with 2.5 nM protease and 4.75 µM SNAP25 FRET substrate. Dashed lines indicate values for the wild-type protease. (**c**) Abundance of top PROSS- and ProteinMPNN-redesigned proteases in purification elution fractions E1-E4 from the soluble fraction of *E. coli* expression cells. 5 µl out of approximately 1 ml of elution fraction were loaded into each lane. (**d**) Protein thermal melting curves to measure melting temperatures. Thermal unfolding was followed by SYPRO Orange fluorophore and Tm values were calculated as the temperature at which slope of the curve is maximal. (**e**) Michaelis-Menten plot for in vitro cleavage kinetics with the SNAP25 FRET substrate and resulting kinetic parameters for BoNT/E protease variants. In **b**, values represent the mean of two replicates. In **d-e**, values and error bars represent mean ± s.d. of three replicates.

To compare BoNT protease redesign by ProteinMPNN to earlier redesign approaches, we redesigned BoNT/E with PROSS, a computational method that predicts stabilizing mutations using Rosetta energy calculations and evolutionary statistics^38^. Applying the same distance constraints as for ProteinMPNN, we designed and experimentally assayed 82 PROSS-generated variants for activity and soluble yield. PROSS sequences had fewer total mutations, fewer mutations to or from charged residues, and more conservative mutations than those generated with ProteinMPNN (Supplementary Figs. 2 and 3). Of these redesigns, 33 (40%) were at least as active and as highly expressed as WT BoNT/E (Extended Data Fig. 2a,b). Three selected top-performing redesigns (PROSS1–PROSS3) were purified at large scale. The PROSS redesigns showed enhanced expression, stability and enzyme kinetics similar to those of ProteinMPNN D1–D3 (Extended Data Fig. 2c–e), although the top-ranked ProteinMPNN redesigns had a higher average melting temperature (*T*_m_; 55.2 °C) than the top-ranked PROSS redesigns (52.3 °C; Extended Data Fig. 2d).

**Extended Data Fig. 3:**
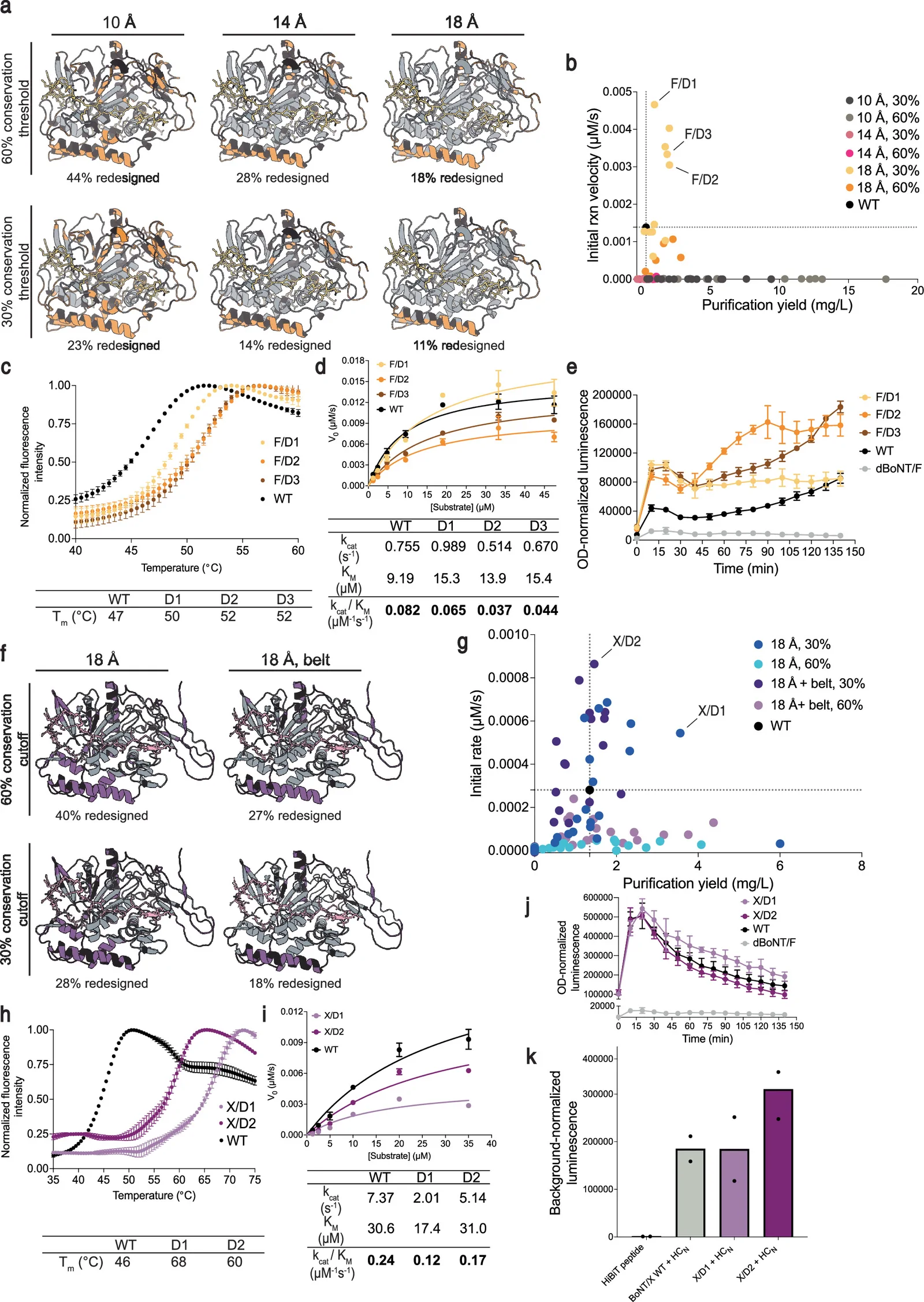
ProteinMPNN redesign and characterization of stabilized BoNT/F and BoNT/X proteases. (**a**) Variable and constrained sequence mapped to the BoNT/F protease structure by constraint group. Gray, fixed by distance; black, fixed by conservation; orange, redesigned. (**b**) Catalytic rate and soluble yield for assayed designs. Cleavage assays were conducted with 10 nM protease and 4.75 µM VAMP1 FRET substrate. Dashed lines indicate values for the wild-type protease. (**c**) Protein melting curves to measure thermal stability (top) and resulting melting temperatures (bottom). (**d**) Michaelis-Menten plot for in vitro cleavage kinetics with native substrate (top) and kinetic parameters (bottom) for BoNT/F protease variants. (**e**) Apparent protease activity in the PACE host measured by luciferase signal. (**f**) Variable and constrained sequence mapped to the BoNT/X protease structure by constraint group. Gray, fixed by distance; black, fixed by conservation; purple, redesigned. (**g**) Catalytic rate and soluble yield for assayed designs. Cleavage assays were conducted with 2.5 nM protease and 4.75 µM VAMP1 FRET substrate. Dashed lines indicate values for the wild-type protease. (**h**) Protein melting curves to measure thermal stability (top) and resulting melting temperatures (bottom). (**i**) Michaelis-Menten plot for in vitro cleavage kinetics with native substrate (top) and kinetic parameters (bottom) for BoNT/X protease variants. (**j**) Apparent protease activity in the PACE host measured by luciferase signal. (**k**) Intracellular delivery of HiBiT-tagged redesigned proteases with BoNT/X toxins in N2A cells, measured by luminescence signal upon luciferase complementation. Values and error bars represent mean ± s.d. of three replicates. Bars in k represent the average of two biological replicates.

BoNT proteases from different serotypes have distinct specificities that offer alternative starting points for evolution. We performed a similar redesign and characterization procedure on BoNT/F protease and BoNT/X protease (Supplementary Note 1). Characterization of the top three BoNT/F redesigns confirmed higher stability and PACE circuit activation than the WT protease, and similar catalytic efficiencies (Extended Data Fig. 3a–e). The top two BoNT/X designs showed successful stabilization as well as compatibility with the toxin heavy chain module for mammalian intracellular delivery (Extended Data Fig. 3f–j). Together, the results for BoNT/E, BoNT/F and BoNT/X proteases demonstrate that the redesign process can stabilize proteases with distinct substrate specificities, while retaining or improving catalytic efficiency compared with the corresponding WT enzyme.

The above redesign campaigns provided a suite of three types of stabilized, highly active BoNT proteases with the potential to be reprogrammed in PACE. ProteinMPNN redesign performance varied across proteases, but characterization of a practical number (96 or fewer) of redesigned variants in all tested cases yielded proteases with improved stability and retained or improved activity.

## Redesign enhances an evolved protease

**Fig. 2:**
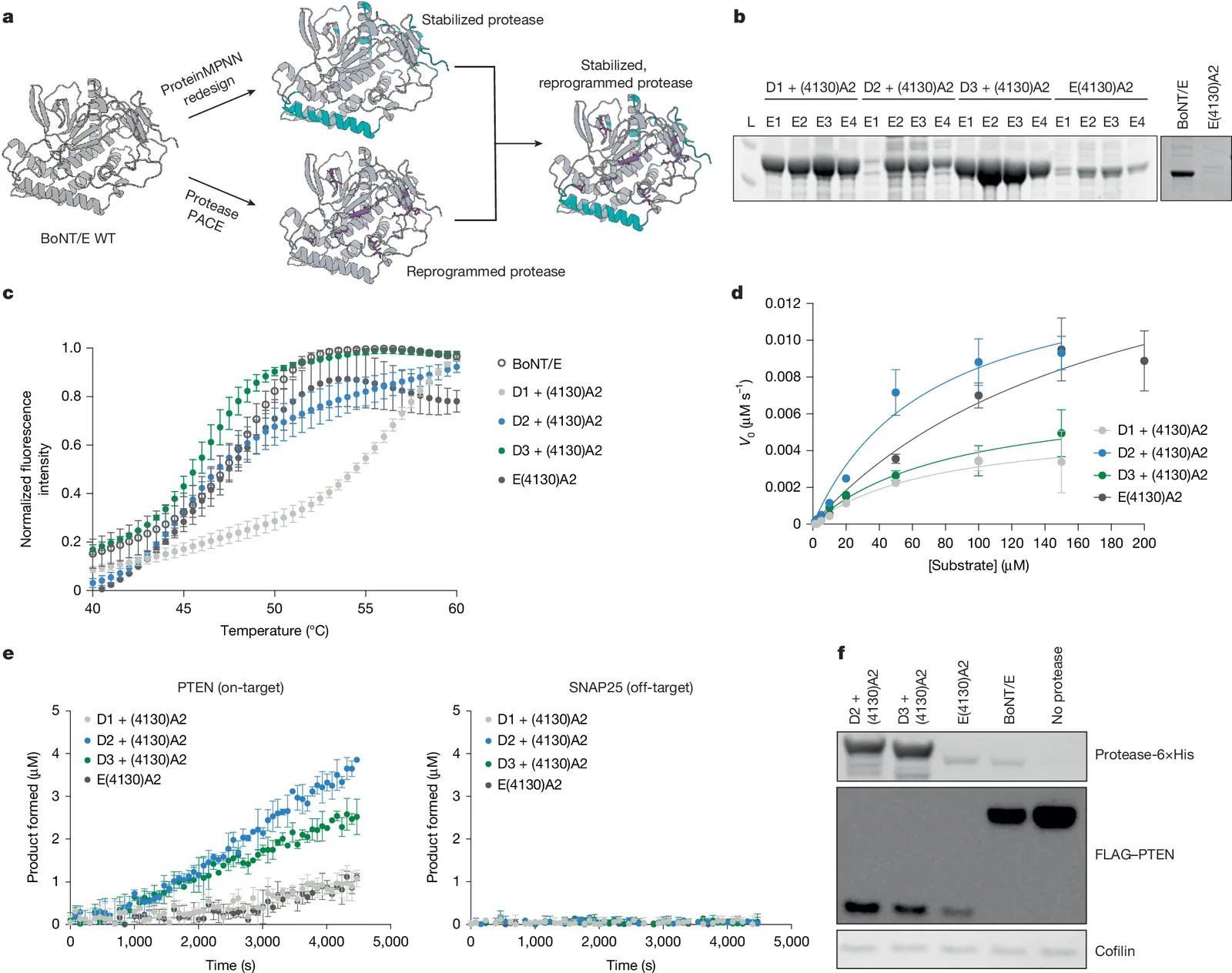
Combining PACE-evolved BoNT/E mutations with ProteinMPNN redesign enhances expression level and activity of reprogrammed PTEN-cleaving BoNT/E protease variants. **a**, Grafting mutations that evolved during PACE of WT BoNT/E protease to cleave PTEN onto a ProteinMPNN-stabilized variant of WT BoNT/E. **b**, Abundance of PTEN-cleaving proteases in purification elution fractions E1–E4 from the soluble fraction of *E. coli* expression cells. **c**, Protein melting curves for PTEN-cleaving proteases measured by SYPRO orange fluorescence. *T*_m_ values, calculated as the temperature at the maximal slope of the melting curve, are provided in Supplementary Table 6. **d**, Michaelis–Menten plot for in vitro cleavage kinetics with PTEN substrate. Kinetic parameters for PTEN-cleaving proteases are provided in Supplementary Table 5. **e**, Cleavage time course comparing on-target PTEN FRET substrate cleavage (left) and off-target SNAP25 FRET substrate cleavage (right) with 10 nM protease and 4.75 µM substrate. **f**, Co-transfection in human HEK293T cells of plasmids expressing pleckstrin domain-tagged PTEN-cleaving BoNT/E proteases and FLAG-tagged PTEN to assess PTEN cleavage from reprogrammed proteases in mammalian cells. Protease and substrate plasmids were co-transfected at a mass ratio of 1:1. The band intensity reflects protease abundance (top), PTEN substrate and cleavage product abundance (middle), or cofilin loading control (bottom). The values and error bars represent mean ± s.d. of three replicates (**c**–**e**).

Next, we investigated whether redesign could rescue the degraded stability of a highly evolved protease while retaining its novel specificity. We have previously evolved BoNT/E to cleave PTEN, a target of therapeutic interest unrelated to any known BoNT protease substrate, while greatly reducing activity on the native SNAP25 substrate^11^. The resulting evolved E(4130)A2 protease, however, cleaves PTEN at a 11-fold slower rate than WT BoNT/E cleaves SNAP25. We tested whether the ProteinMPNN-redesigned BoNT/E variants could be combined with PACE-evolved mutations by grafting the 16 PACE mutations of E(4130)A2 into the top redesigned BoNT/E backgrounds D1, D2 and D3 (Fig. 2a). E(4130)A2 expresses more poorly and is less stable than WT BoNT/E protease (Fig. 2b,c). We observed that the mutation-grafted D1, D2 and D3 enzymes (designated D1 + (4130)A2, D2 + (4130)A2 and D3 + (4130)A2) express to a higher level in *E. coli* cells than E(4130)A2 by 4.2-fold, 2.2-fold and 5.2-fold, respectively (Fig. 2b). The grafted proteases retained their ability to cleave PTEN, D2 showed improved kinetics and D1 showed improved thermal stability compared with E(4130)A2 (Fig. 2c,d and Supplementary Tables 5 and 6). Activity on the original SNAP25 substrate was undetected for all variants (Fig. 2e), demonstrating that the redesigned proteases preserve the reprogrammed specificity that E(4130)A2 evolved in PACE.

To assess these proteases in human cells, we co-transfected HEK293T cells with protease expression plasmids and a PTEN expression plasmid. The two mutation-grafted redesigned proteases were expressed to a much greater level (more than 24-fold) than either E(4130)A2 or WT BoNT/E resulting in 4.5-fold and 3.9-fold higher product formation levels for D2 + (4130)A2 and D3 + (4130)A2, respectively (Fig. 2f). These results together demonstrate that ProteinMPNN redesign of a PACE-evolved enzyme can improve expression and stability while retaining evolved specificity, leading to a much greater level of target substrate proteolysis in mammalian cells.

## Redesign improves evolution outcomes

We hypothesized that the greater stability of redesigned proteases may enhance evolvability on a new substrate by offering compatibility with mutations that improve desired activity but decrease protein stability. We performed a panel of PACE campaigns for new substrate cleavage in which we evolved the top redesigned, stabilized BoNT/E protease in parallel with the WT BoNT/E and compared outcomes between the two starting points.

**Extended Data Fig. 4:**
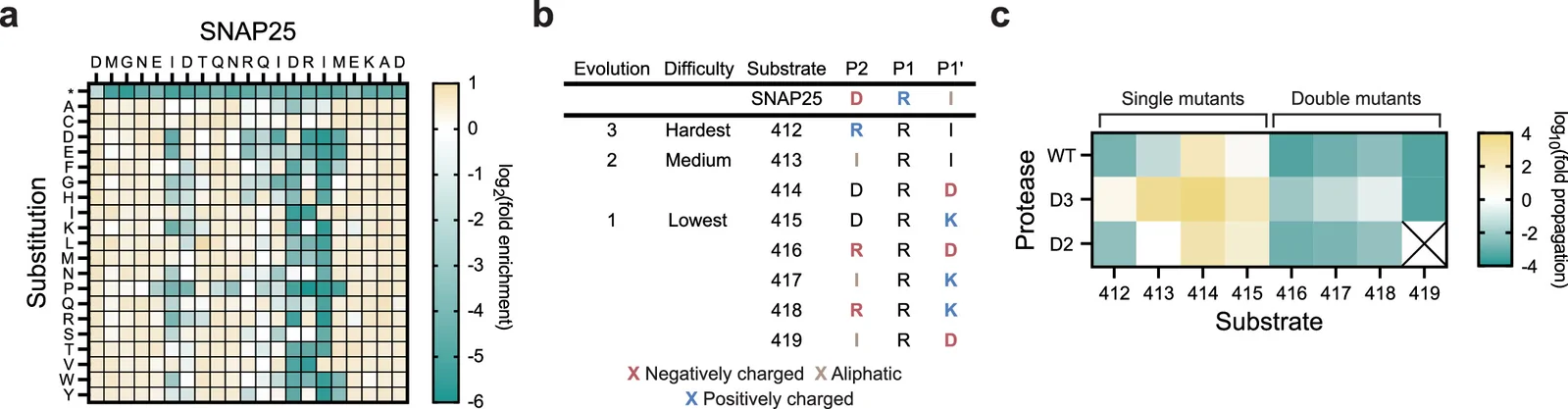
Identification of substrates for parallel evolutions. (**a**) Substrate profiling assay of initial starting activity of BoNT/E protease on all single mutants of 21 residues of SNAP25. Enrichment indicates activity on each mutant substrate. (**b**) Sequence comparison of native SNAP25 substrate with selected new substrates for evolution color-coded by each mutation’s biochemical properties. (**c**) Starting fitness measured by overnight propagation assay of phage encoding wild-type BoNT/E, D3, and D2 proteases on substrates 412-419.

To identify substrates representing a range of challenge, we performed substrate profiling to measure BoNT/E protease activity on all 441 possible SNAP25 single-residue variants. We selected three substrates (415, 413 and 412) of increasing difficulty based on their decreasing starting activity levels (Extended Data Fig. 4). We performed evolution on each substrate in 3–4 replicate lagoons for each starting protease. To enable 22 independent PACE lagoons in this experiment, we performed ePACE using autonomous eVOLVER and min-eVOLVER devices that facilitate parallel PACE experiments^45–47^. We assayed consensus evolved variants to determine fitness (circuit activation), and for enzyme kinetics, stability and soluble expression levels. Finally, mutations derived from either protease starting point (WT or redesigned) were grafted into the other protease background to determine whether the evolved mutations arose because of differences in starting-point robustness, or whether instead they were simply the result of evolutionary stochasticity and could benefit either starting point.

**Fig. 3:**
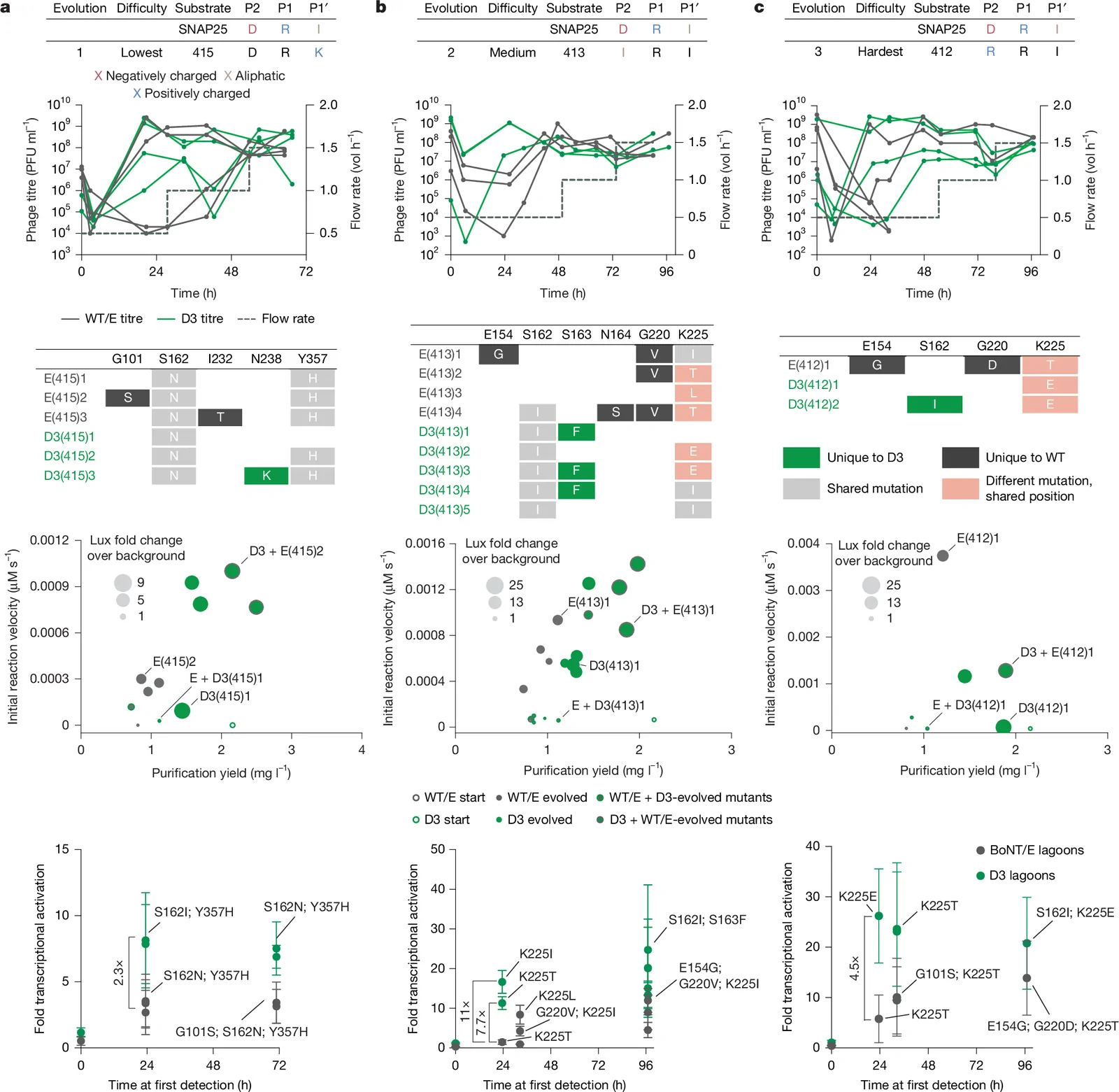
The evolvability of sequence-designed proteases on a panel of PACE substrates. **a**–**c**, Evolutions 1, 2 and 3 on substrates 415 (**a**), 413 (**b**) and 412 (**c**). First row, comparison of SNAP25 and substrate sequences (colours indicate amino acid property). Second row, ePACE phage titres in three or four replicate lagoons initiated with each of the WT and D3 starting proteases. Third row, consensus genotypes from both starting points at the final PACE timepoint with mutations coloured by whether they were unique to either starting point. Fourth row, assay of all variants and the grafts of mutations derived from each starting point into the alternative background measuring soluble yield, initial catalytic rate with the cognate FRET substrate, and PACE circuit activation measured by luciferase signal represented by point size. Fifth row, luciferase-reported circuit activation of intermediate and final consensus variants with their cognate evolution substrate by time at which they were first detected by clonal sequencing. The values represent the mean of two replicates (fourth row panels in **a**–**c**), and the values and error bars represent the mean ± s.d. of three replicates (fifth row panels in **a**–**c**).

On the least challenging substrate 415 (P1′ I→K), both the D3 and WT proteases evolved solutions, but the D3-evolved proteases reached higher levels of activity than the WT-evolved proteases (Fig. 3a). The three D3-evolved enzymes resulted in 7.7-fold, 6.9-fold and 6.1-fold protease cleavage circuit activation over background, compared with 2.6-fold, 3.0-fold and 2.8-fold activation for the WT-evolved proteases. Both starting proteases independently evolved S162N and Y357H mutations. However, these mutations in the D3 protease background, genotype D3(415)2, resulted in 7.7-fold circuit activation, whereas the same two mutations in the WT background, E(415)1, resulted in only 2.6-fold activation.

Beyond these shared mutations, the D3 and WT BoNT/E proteases evolved mutations unique to each starting point, such as G101S and I232T from WT BoNT/E and N238K from D3 (Fig. 3a, third row). WT BoNT/E-derived genotypes with mutations G101S and I232T were compatible with the D3 background: grafting either of these genotypes into the D3 background generated proteases with high PACE circuit activation, purification yield and kinetics. By contrast, the genotype with the N238K mutation evolved by the D3 protease only modestly benefited the WT background, increasing circuit activation when grafted into the WT protease only 1.5-fold, whereas the same genotype in the D3 background enhanced luciferase activation 5.6-fold (Fig. 3a, fourth row). These results demonstrate how redesign of BoNT/E protease into D3 expanded the space of beneficial mutations accessed during PACE to include variants that the WT protease was unable to fully leverage. Apart from end point performance, we also assessed how variants evolved from each starting point perform over the time course of the evolution. Redesign-evolved clones outperform WT-evolved clones by a similar margin at intermediate and final timepoints, indicating that the two starting points approached their peak function during evolution at similar rates (Fig. 3a, fifth row).

The intermediate difficulty evolution on substrate 413 (P2 D→I) resulted in similar outcomes. Once again, redesign-evolved variants attained higher activity than those evolved from WT BoNT/E protease (Fig. 3b). D3-evolved genotypes achieved higher levels of circuit activation of up to 15-fold than the WT-evolved genotypes of up to 8.5-fold. The soluble expression of the design-evolved and D3 background proteases was consistently higher than those of the WT proteases. We observed that the D3 protease again potently benefited from the WT-evolved mutations, whereas the WT BoNT/E protease background showed no activity when hosting the D3-evolved mutations (Fig. 3b, bottom). These data reinforce that design-evolved genotypes demonstrate higher activity, soluble yield and kinetics on the new substrates after PACE than their WT-evolved counterparts, and the D3 background offers compatibility with a greater variety of mutations than WT BoNT/E protease.

We observed that D3 lagoon phage titres during PACE recovered earlier than WT lagoons (Fig. 3b, top, and Supplementary Table 8), suggesting that D3 evolved to pass the selection before the WT enzyme. We investigated the circuit activation of consensus clones at the timepoints over which WT lagoon titres recover and D3 lagoon titres remain high. The WT-evolved consensus mutant at the earliest assayed timepoint, K225T, showed minimal circuit activation of only 1.47-fold above background. At the same timepoint, K225T also evolved in D3 lagoons alongside K225I, both reaching more than 10-fold circuit activation (Fig. 3b, bottom). These genotypes are each a single-base-pair mutation from their respective starting points, and WT-evolved consensus clones did not reach substantial circuit activation until genotypes with two to four DNA mutations arose (Fig. 3b, bottom). As genotypes with multiple mutations appear at later timepoints than single mutants, the analysis of intermediate activity suggests that redesign expanded functional sequences to include those closer to the starting point, allowing the redesigned protease to adapt to the 413 substrate in less time and with fewer mutations than the WT protease.

**Extended Data Fig. 5:**
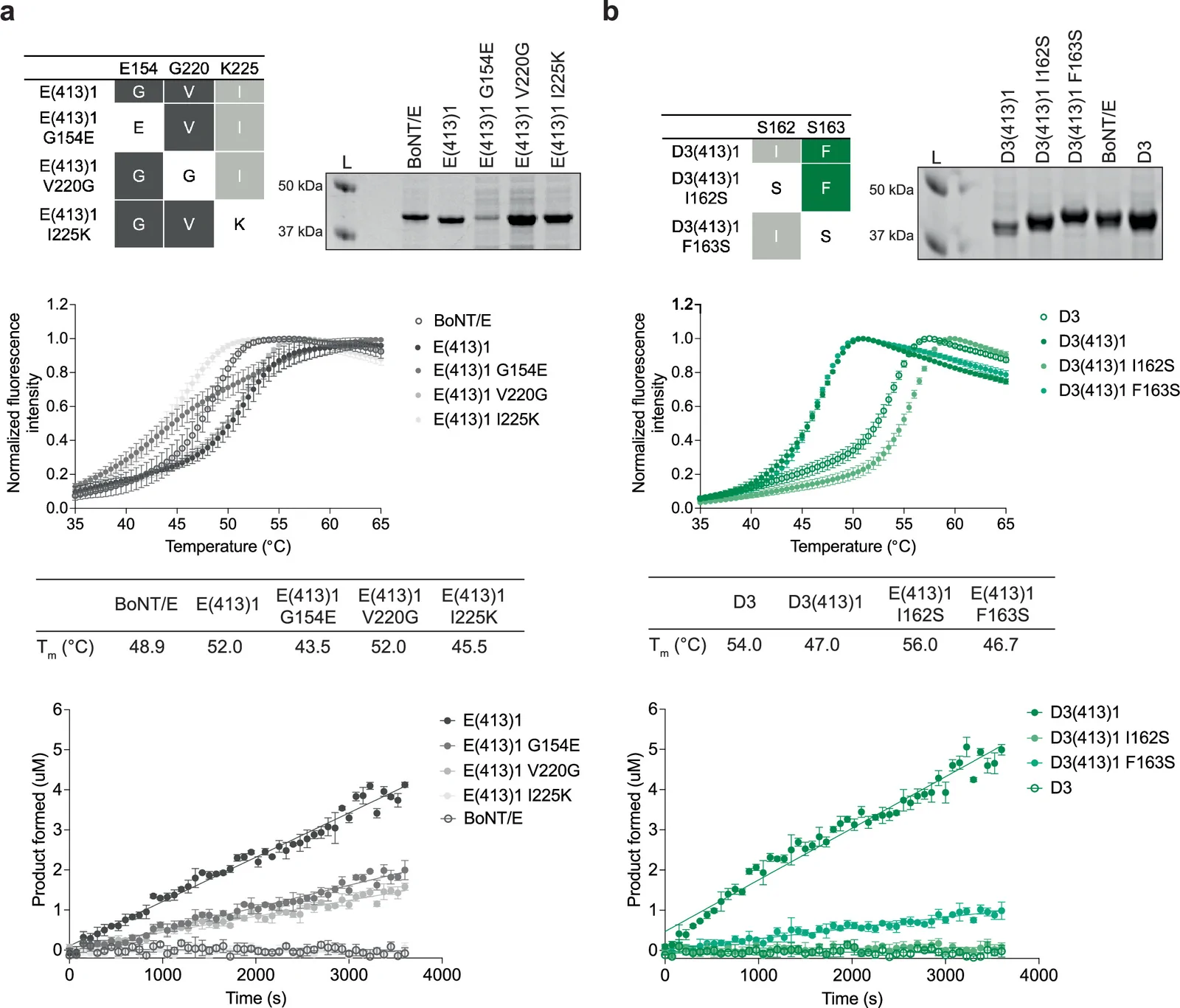
Contribution of individual mutations to expression, stability, and activity in high-activity clones. (**a** and **b**) Reversion analysis of top performing genotypes evolved in the intermediate difficulty (substrate 413) evolution from the (**a**) wild-type or (**b**) D3 starting point. Mutations in evolved variants (top left) were selected for reversion and characterization. Abundance in purification elutions from the soluble fraction of *E. coli* expression cells. (top right). Gels were loaded with 5 µl out of 2.5 ml of total elution each lane. Protein melting curves to measure thermal stability and resulting melting temperatures (middle). In vitro cleavage time course of the 413 FRET substrate at 10 µM with 10 nM protease. Values and error bars represent mean ± s.d. of three replicates.

To understand how evolved variants gain function as they accumulate mutations over time in the intermediate difficulty evolution, we dissected the contributions of individual mutations from high-activity end point genotypes on expression, stability and cleavage activity. For the top-performing WT-evolved triple mutant E154G; G220V; K225I, reverting the E154G mutation yielded a poorly expressed and strongly destabilized enzyme, demonstrating that this mutation compensates for the active but destabilized G220V; K225I double mutant (Extended Data Fig. 5a). By contrast, for the top-performing D3-evolved double mutant variant S162I; S163F from this selection, activity was diminished, but not expression or stability, when either mutation was reverted (Extended Data Fig. 5b). Both of the mutations in the D3-evolved variant were therefore not compensatory. These observations demonstrate how the WT starting point must navigate a more limited path through the fitness landscape that can require compensatory stabilizing mutations to reach high activity, whereas the redesign has sufficient stability to access high activity without relying on the co-evolution of compensatory mutations.

Finally, we evolved both starting point proteases on the most challenging substrate 412 (P2 D→R; Fig. 3c). Out of the four parallel replicates conducted for both starting points, two evolutions starting with the WT protease failed to evolve any activity on the new substrate before phage washout. By contrast, all four parallel evolutions of the D3 protease successfully evolved solutions to the selection (Supplementary Table 8). Thus when faced with a difficult challenge, the ProteinMPNN redesign improved the success rate of the PACE experiment. The two evolutions initiated with the WT protease that succeeded produced a single convergent genotype, whereas the evolutions initiated with the D3 protease produced two genotypes. Once again, the WT-evolved genotype in this evolution, E(412)1, yielded lower protease cleavage circuit activation (10-fold activation over background) than the D3-evolved genotypes that achieved 20-fold and 16-fold activation.

The K225 residue mutated to E in both of the D3-evolved genotypes, whereas the WT protease did not evolve this mutation. Indeed, we found that the K225E mutation conferred no activity when grafted into the WT protease background (Fig. 3c, bottom). These data suggested that only D3 is compatible with K225E because this mutation is destabilizing. Indeed, K225E reduced the *T*_m_ of the WT and D3 proteases by 4.3 °C and 6.5 °C, respectively, such that the WT protease with the K225E mutation had a *T*_m_ of only 45 °C, whereas D3 with K225E (D3(412)1 variant) had a *T*_m_ of 48 °C. Furthermore, the melting transition for the K225E–WT graft occurred over a wider temperature range, suggesting that partial unfolding occurs at even lower temperatures. Similar observations of lower *T*_m_ values and broader transitions were made for design-evolved mutations from the 415 and 413 evolutions that do not function in the WT background (Supplementary Fig. 4). These results reveal that the stabilized D3 protease was able to accommodate destabilizing mutations to enable high activity on the target, whereas the WT protease did not have sufficient stability to fold with these destabilizing mutations.

We again compared consensus clone fitness at intermediate timepoints, and observed that although both the WT and the D3 lagoons evolved active, single-base-pair mutant clones at the earliest assayed timepoint, the D3-evolved clone supported 4.5-fold greater selection circuit activation than the WT-evolved clone. Furthermore, the intermediate time point D3 mutant was as active as alternative and higher-order mutants found at later timepoints. The intermediate timepoint WT clone was 2.4-fold less active than the best final timepoint clone, a triple mutant, demonstrating that D3 required less time and fewer mutations than the WT starting point to reach its maximal evolved activity (Fig. 3c, bottom). This result is consistent with the 413 substrate adaptation, where functional access to local mutants from the redesigned starting point increased adaptation rate.

Collectively, our results from evolving a redesigned protease side-by-side with the corresponding WT protease to cleave three new substrates demonstrate that the enhanced stability of the D3 starting point protease enabled, across all evolutions, higher evolved function and the productive use of mutations that function poorly with the WT protease. When the redesign but not the WT starting point evolved high activity in local, rapidly sampled genotypes, the redesign adapted faster. Greater soluble expression, stability and faster kinetics thus account for the much more beneficial effect of the same mutations in the D3 protease background compared with the WT BoNT/E background.

## Progression of genotype frequencies

**Fig. 4:**
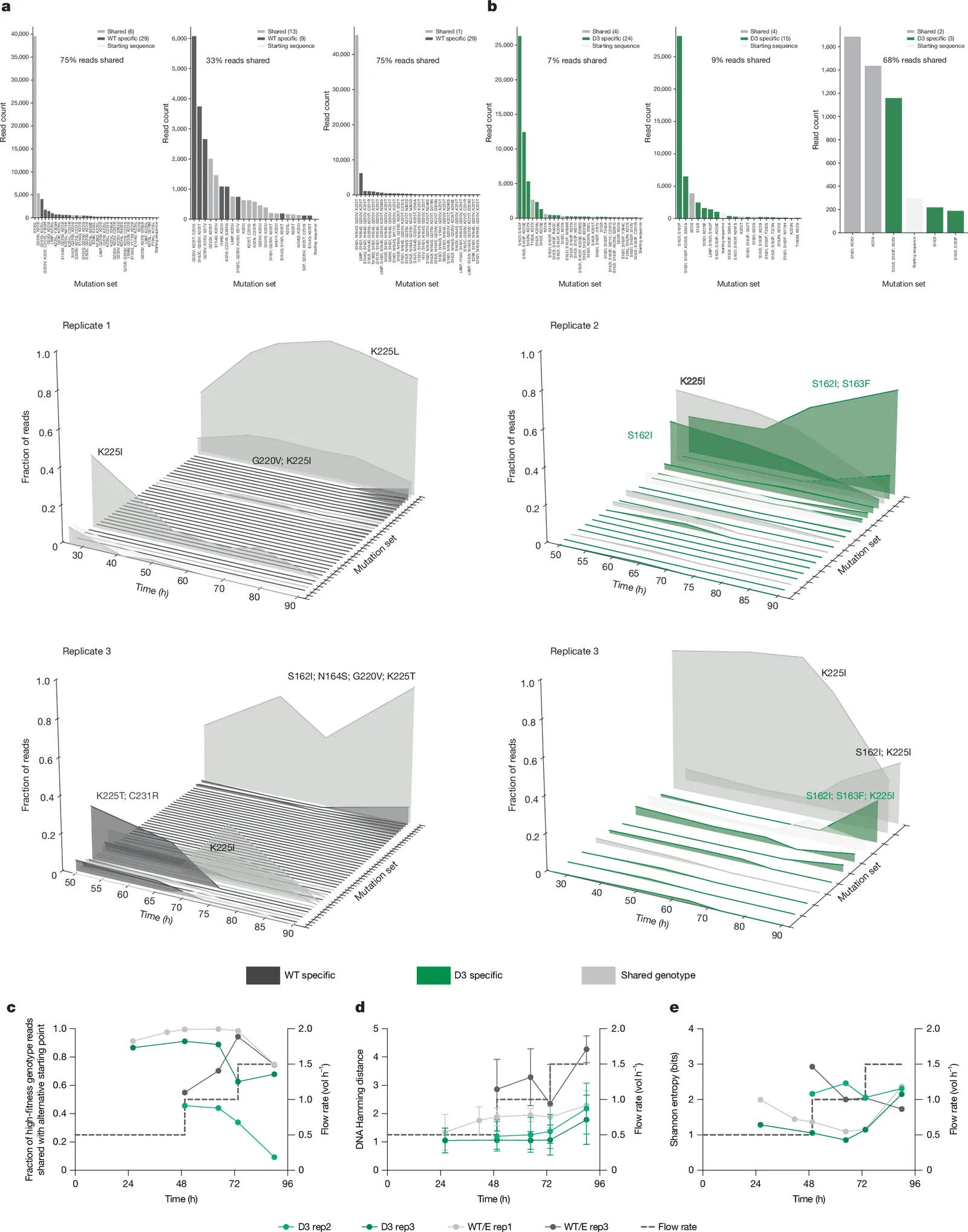
Genotype distributions and starting point-specific mutation sets over the course of the intermediate difficulty evolution. **a**,**b**, High-throughput long-read sequencing quantification of WT-evolved (**a**) and D3-evolved (**b**) populations. Distributions of genotypes detected by LoopSeq for the final timepoint for replicate 1 (top left), replicate 2 (top centre) and replicate 3 (top right). Mutation set frequencies are shown by timepoint for two of the replicates: replicates 1 and 3 for WT-evolved and replicates 2 and 3 for the D3-evolved populations (middle and bottom). Mutation sets with more than 100 assembled long reads mapped are shown. Mutation sets are coloured by whether they were unique to either starting point (green for D3, and black for WT) or detected at least once in clones evolved from both starting points (grey) at the final timepoint. **c**, Fraction of reads by timepoint mapping to plotted genotypes and a mutation set observed at least once in the other starting point at the final timepoint (grey genotypes in panels **a**,**b**). **d**, Average Hamming distance to the starting point of reads mapping to plotted genotypes by timepoint. **e**, Shannon entropy of the plotted genotype distribution observed by timepoint. The values and error bars represent the mean ± s.d. of all sample reads mapping to plotted genotypes (**d**).

We performed high-throughput, synthetic long-read sequencing with LoopSeq^48^ to deeply quantify full-length genotype distributions of the D3-evolved and WT-evolved final timepoint lagoons for the 413 substrate. The most abundant WT-evolved mutations often propagated in the D3-evolved lagoons (Fig. 4a, top). By contrast, D3-evolved mutations infrequently propagated in WT-evolved pools (Fig. 4b, top). The relative scarcity of the most fit D3-evolved mutations among the evolved WT genotypes in these lagoons is consistent with our finding that the WT protease background does not support these mutations. These observations reinforce our finding that the redesigned protease starting point can access both the functional mutation space of the WT starting point as well as unique genotypes during evolution.

To analyse how redesign-enabled genotypes arise over time and over increasing selection stringency, we obtained LoopSeq data and quantified genotype distributions across multiple timepoints for two replicates of each starting point for the intermediate evolution. For both redesign-initiated lagoons, WT-compatible genotypes dominated the lagoon at early timepoints but dropped in frequency as redesign-specific genotypes grew in abundance, with replicate 1 converging almost entirely on redesign-specific genotypes (Fig. 4c). Although multi-mutant genotype frequency rose over time, single mutants persisted to the end of the experiment (Fig. 4b). In WT-initiated lagoons, single mutants were mostly replaced by multi-mutant genotypes such as the two-base substitution K225L in replicate 1 and a quadruple mutant in replicate 3 (Fig. 4a). As a result, the WT starting point tended to evolve more mutationally distant lagoons than the D3 starting point (Fig. 4d). This sequence divergence in some cases occurred with a rise in lagoon diversity (Fig. 4e). Together, genotype dynamics suggest that the redesigned starting point supports local genotypes to survive selection, whereas the WT starting point requires more distant genotypes to pass selection.

## Redesign expands the fitness landscape

**Fig. 5:**
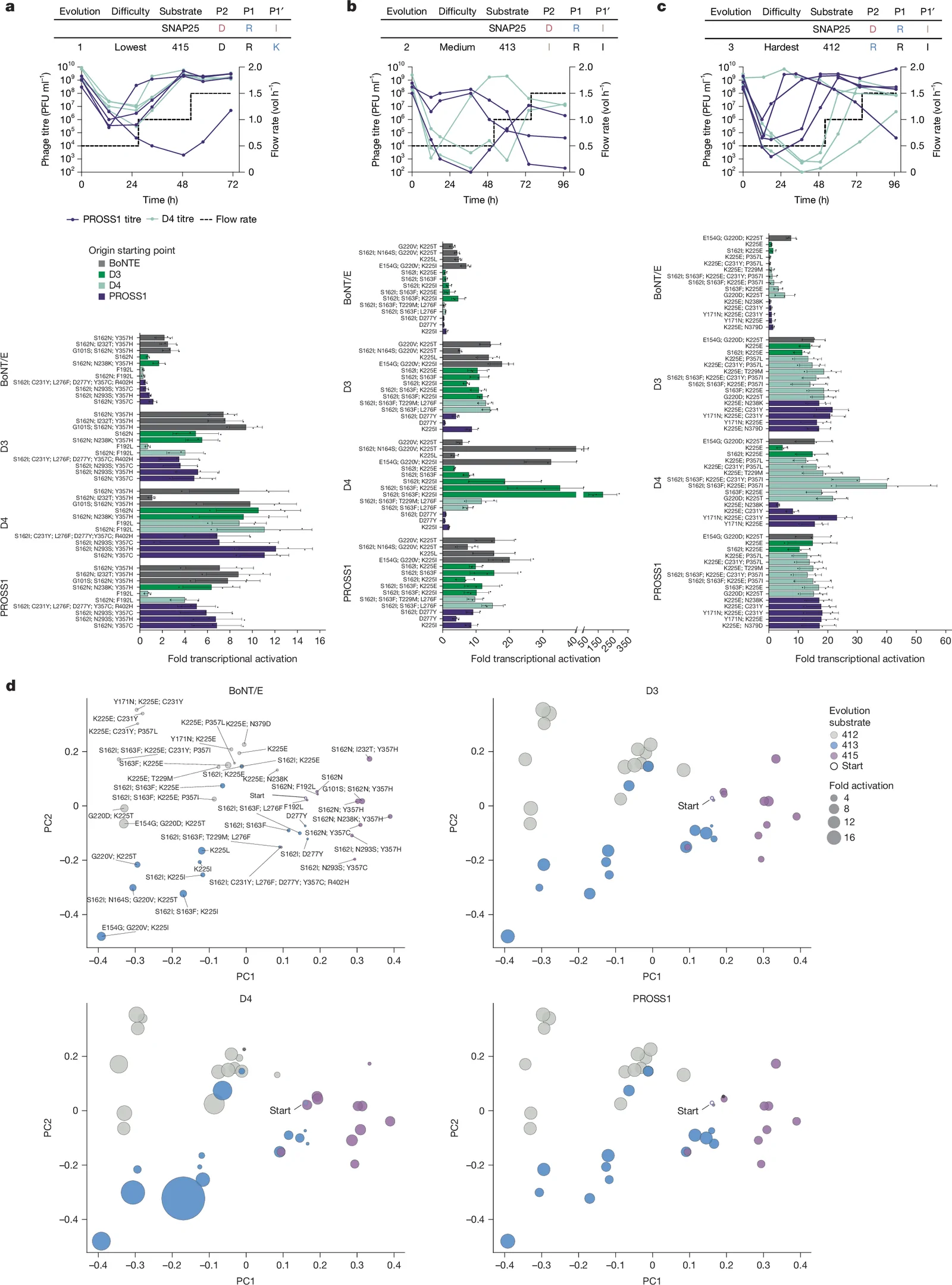
Evolution of alternative redesigns and comprehensive assessment of evolved genotype function in all starting point backgrounds. **a**–**c**, Evolutions with D4 and PROSS1 starting points on substrates 415 (**a**; lowest difficulty), 413 (**b**; intermediate difficulty) and 412 (**c**; highest difficulty), as in Fig. 3. Top, comparison of SNAP25 and target substrate sequences; colours indicate amino acid property as in Fig. 3. Middle, ePACE phage titres in three or four replicate lagoons initiated with each of the D4 and PROSS1 starting proteases; the same PACE flow rate schedule and duration were used as in Fig. 3. Bottom, activity of evolved consensus genotypes in all four starting point backgrounds measured by luciferase-reported circuit activation on the cognate evolution substrate. Variants are organized by the protease background in which each mutation set was assessed, and by the starting point of each evolved mutation set in PACE. Mutations are noted by residue mutated from in the origin starting point, which varies when the mutated position was changed by redesign (see Extended Data Fig. 6a). Signal values were normalized to an inactive dBoNT/F control, and values and error bars represent mean ± s.d. of three replicates. **d**, Consensus genotype activity organized in ESM-C embedding principal component (PC) space for each background. Embedding PCs were generated for the BoNTE sequences with each labelled mutation set, and points were scaled according to activity (top left), then replotted with point scaling by activity of the same mutation sets in the D3, D4 and PROSS1 redesign backgrounds (top right, bottom left and bottom right, respectively). Points with a cross hatch pattern were not assayed.

Following our observation that a stabilized, catalytically enhanced ProteinMPNN redesign evolves distinct, higher-activity genotypes, we investigated how alternative BoNT/E redesigns evolve in PACE and compared how each redesign modifies the fitness landscape. Because stability redesign often trades off with function, we asked whether a highly stabilized but kinetically impaired redesigned enzyme could outperform the WT starting point during evolution. We selected ProteinMPNN redesign D4 as the starting point for this question. D4 showed high expression and low activity in the initial redesign panel (Fig. 1c). Accordingly, D4 cleaves its substrate 20-fold more slowly than WT BoNT/E but has a higher *T*_m_ than both the WT and D3 (Supplementary Fig. 5). To determine whether PROSS redesign can also enhance evolution, we selected the top-performing redesign (PROSS1) with improved stability and catalysis as the final alternative starting point. We performed parallel PACE campaigns initiated with the D4 and PROSS1 starting points on the same panel of substrates that BoNT/E and D3 were previously evolved to cleave, and each evolution followed the same flow rate schedule, duration and number of replicates as before (Fig. 5a–c). Between the four starting points evolved on three substrates with three or four replicates each, we performed a total of 44 independent evolutions on eVOLVER and Min-eVOLVER autonomous culture devices.

**Extended Data Fig. 6:**
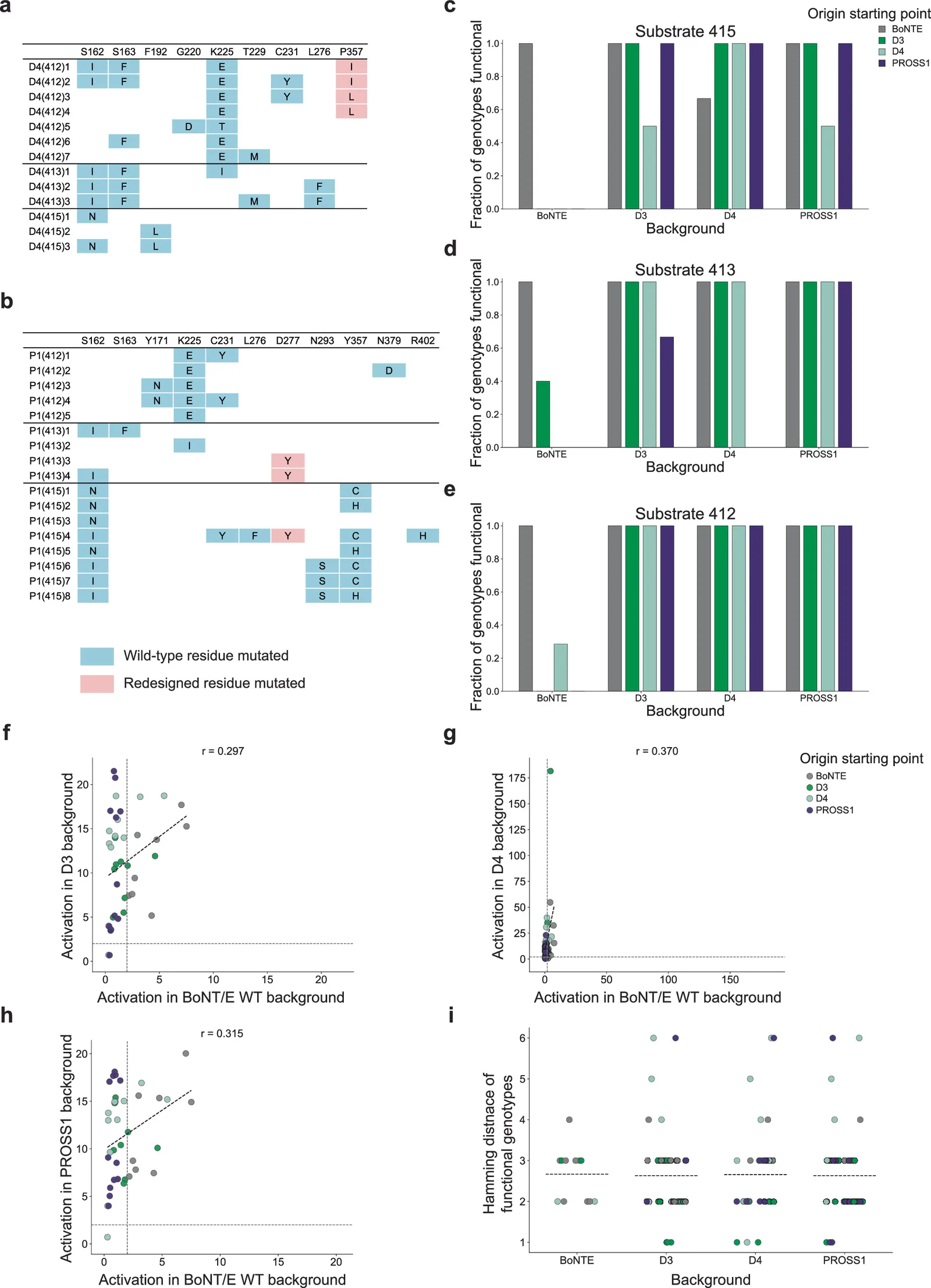
Alternative starting point consensus genotypes and activity grafting these mutations into four protease backgrounds. (**a**) Consensus genotypes from D4 starting point at the final PACE timepoint. (**b**) Consensus genotypes from PROSS1 starting point at the final PACE timepoint. Mutations are colored by whether they occurred at wild-type residues unchanged during redesign (blue) or at redesigned residues (pink). (**c** to **e**) Fraction of consensus genotypes for each origin starting point that are functional on their cognate evolution substrate (415, 413, and 412, respectively) in each background. (**f** to **h**) Correlation of genotype circuit activation over inactive control for each mutation set grafted into a redesigned protease background vs. into the wild-type BoNT/E background. Dashed lines indicate the activation threshold defining a functional genotype. Correlation r values are Pearson correlation coefficients. (**i**) Distribution of functional genotype Hamming Distance for each protease background for all assayed genotypes. Dashed lines indicate the mean for each background. In c-i, a genotype is defined as functional if its luciferase circuit activation level is > 2-fold above that of the inactive dBoNT/F protease control.

The D4-evolved and PROSS1-evolved populations passed selection on all three substrates. Sequencing of clones from final timepoint samples revealed consensus genotypes distinct from those evolved from the WT or D3 starting points (Fig. 3a–c and Extended Data Fig. 6a,b). We asked how the D4-evolved and PROSS1-evolved variants compare in activity with those evolved from the WT and D3 starting points. Furthermore, we wondered how the alternative starting points function with the genotypes evolved from all other starting points. To answer these questions, we performed a comprehensive assessment of genotype activity across various genetic backgrounds, in which we assayed the circuit activation of consensus genotypes evolved from each starting point in all four protease backgrounds after evolution on all three substrates (Fig. 5a–c, bottom).

The three redesign-evolved variants were consistently more active than WT-evolved variants across all three substrates. D3-evolved and PROSS1-evolved proteases achieved similar function, whereas the D4-evolved proteases were as active or more active than those evolved from the other redesigns, despite the 20-fold lower starting catalytic rate for D4 (Fig. 5a–c, bottom, bars with matched background and origin starting point). This result is consistent with our earlier finding that D3 evolves higher fitness than WT. Alternative redesigns therefore enhance evolvability, even when redesign impairs catalysis.

Next, we evaluated evolved genotypes grafted into each alternative background. Each redesign was highly functional with most genotypes from the WT-evolved and other redesign-evolved variants. These graft variants tended to have similar or higher function than the redesign-evolved variants. However, some genotypes were highly active only in the background from which they evolved. Although D4 was highly active with most genotypes, its performance was variable, with some genotypes reaching the highest activity of any variant across backgrounds and others failing to function despite their activity in D3, PROSS1 or even BoNT/E backgrounds (Fig. 5a–c, bottom, and Extended Data Fig. 6c–e). The variable performance of evolved genotypes in the D4 background may result from a robustness to destabilizing mutations balanced with a sensitivity with catalytically slow mutations.

In contrast to the robustness of the redesigned proteases to graft genotypes, the WT enzyme supported systematically lower activity with redesign-grafted genotypes than with its own evolved genotypes. Most redesign-evolved mutations in the WT background were non-functional; the WT background yielded zero functional proteases when grafting redesign mutants for the lowest-difficulty 415 substrate, and was functional with only a minority of redesign-evolved mutants for the intermediate 413 and highest-difficulty 412 substrates (Extended Data Fig. 6c–e). We further found that activity for each genotype correlates poorly between each redesign and the WT background, supporting the idea that the redesigns have high activity with genotypes that rank poorly in the WT background (Extended Data Fig. 6f–h). Evolved genotype function across all four backgrounds demonstrates that alternative redesigns exceed the WT background function with distinct evolutionary solutions. The variable function of some genotypes between redesign backgrounds suggests that the balance between stability and activity in redesign, as well as the particular redesigned residues, influence the precise functional sequence space available.

To visualize how redesign reshapes fitness across sequence space, we analysed the activity of evolved and graft genotypes in the embedded space of a protein language model^49^. The model ESM-C successfully clusters all evolved genotypes in the BoNT/E WT background by the substrate they were evolved to cleave (Fig. 5d, top left). The WT BoNT/E fitness landscape is sparse, with the highest function variants for each substrate distal to the starting point sequence. The sequences immediately surrounding the starting point are poorly active, and none of the single-base-pair mutant genotypes for any substrate functions in this WT background (Extended Data Fig. 6c–e). By contrast, each redesign raises the average fitness level across all three substrates. Redesign enables function with new distal genotypes, as well as with genotypes more proximal to the starting point, including single-base-pair mutations (Fig. 5d and Extended Data Fig. 6i). The robustness to local mutants supports our earlier interpretation of how redesign can accelerate adaptation. Collectively, this analysis points to a model in which redesign raises fitness and expands the fitness landscape across selections.

**Extended Data Fig. 7:**
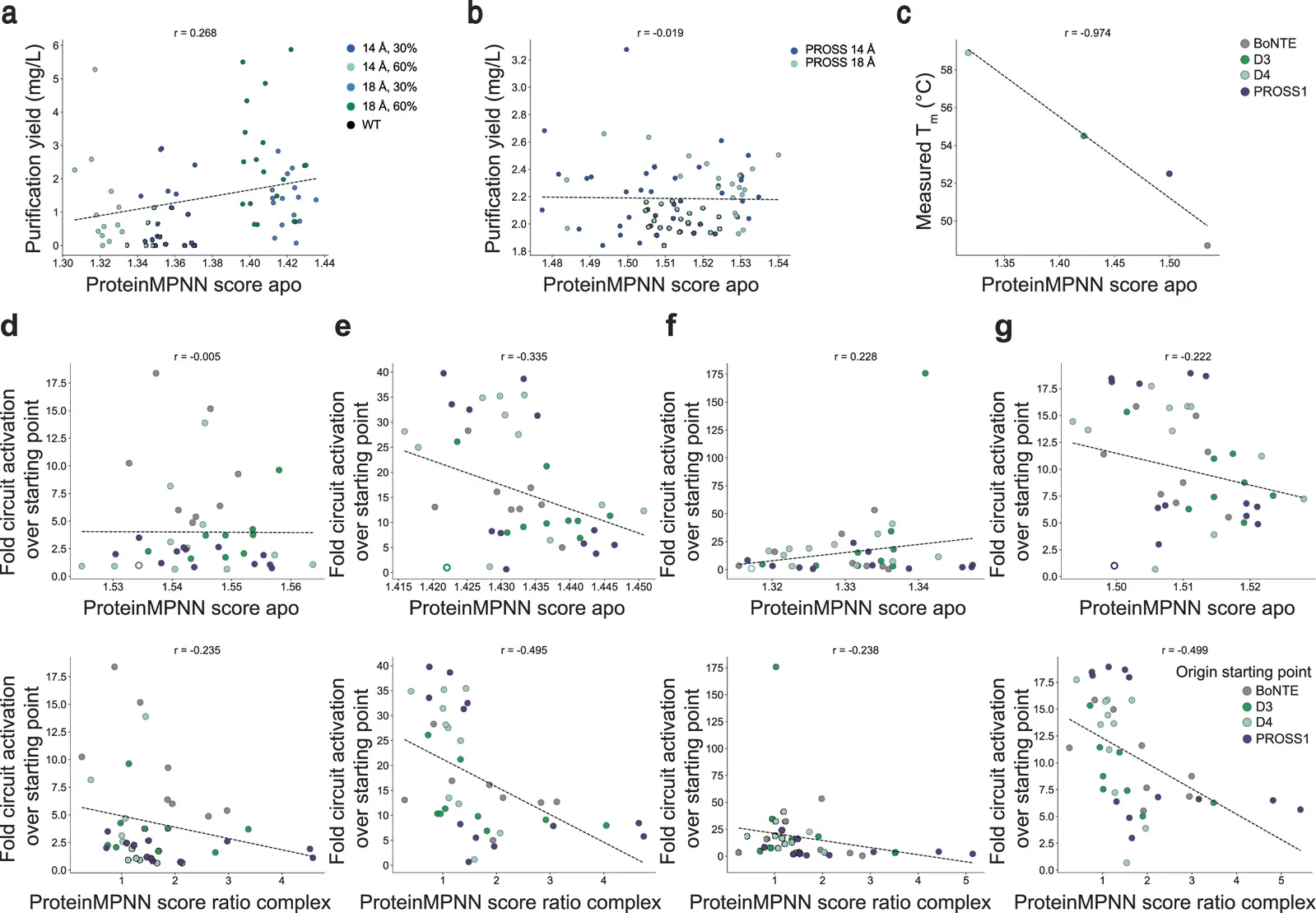
The relationship between redesign objective scores, evolvability, and evolved mutant activity. (**a**) Correlation of BoNT/E ProteinMPNN redesign soluble yield with ProteinMPNN apo score. (**b**) Correlation of BoNT/E PROSS redesign soluble yield with ProteinMPNN apo score. (**c**) Experimentally measured protein melting temperature vs. ProteinMPNN apo score for evolution starting points. (**d** to **g**) Correlation of starting point-normalized circuit activation by luciferase reporter to ProteinMPNN apo scores (top) and to complex scores for evolved residues normalized to unevolved residue scores (bottom) in BoNT/E, D3, D4, and PROSS1, backgrounds, respectively. ProteinMPNN scores are given as residue-averaged negative log-likelihoods (lower values are more likely) for the Alphafold3-predicted BoNT/E protease structure alone (apo) or bound to the cognate substrate (complex), averaged over 10 replicate calculations. Correlations are labeled with Pearson’s r coefficients.

We also asked whether redesign evolvability and evolved genotypes could be predicted from computational redesign metrics. Analyses of these scores suggest that they could contribute, albeit modestly, to predicting evolutionary performance and mutant function (Extended Data Fig. 7 and Supplementary Note 2).

## Evolving specific ataxin-2 proteases

**Fig. 6:**
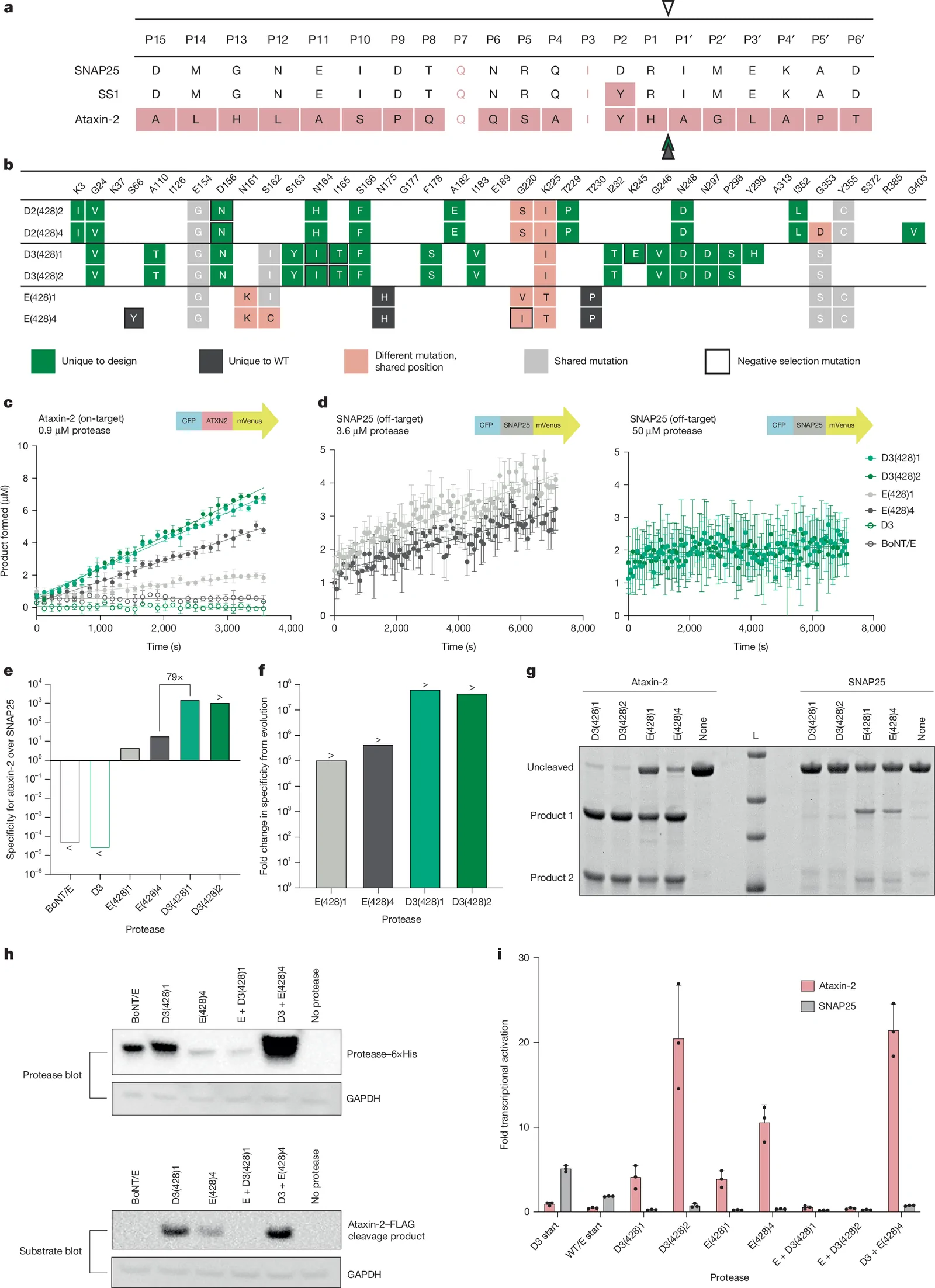
Reprogramming sequence-redesigned versus WT proteases to specifically cleave a therapeutic, non-native target over the native target. **a**, Comparison of native, stepping-stone and final target substrate sequences. The white, grey and green arrowheads indicate cleavage sites of WT BoNT/E, E(428)4 and D3(428)2, respectively. SS1, stepping-stone 1. **b**, Final consensus clones arising from each starting point. **c**, Ataxin-2-targeting protease in vitro cleavage rates of ataxin-2 FRET substrate at 20 µM. **d**, Ataxin-2-targeting protease in vitro cleavage rates of SNAP25 FRET substrate at 20 µM. **e**, Specificity of starting and evolved proteases, defined as cleavage rate on ataxin-2 over cleavage rate on SNAP25. **f**, Change in specificity from evolution, defined as evolved protease specificity divided by starting protease specificity. The < and > symbols indicate a measurement bounded by assay detection limit (**e**,**f**). **g**, Gel shift assay for on-target and off-target cleavage. Substrates at 15 µM were incubated with 3.6 µM protease for 20 h. L, ladder. **h**, Co-transfection assay for expression of evolved proteases and ataxin-2 cleavage product formation in HEK293T cells. Protease and substrate plasmids were co-transfected at a mass ratio of 1:1 for protease expression detection and a ratio of 1:10 for cleavage detection. The band intensity reflects protease abundance (top) or cleavage product abundance (bottom) or GADPH loading control. **i**, Apparent protease activity of evolved and evolution mutation-grafted variants measured by luciferase signal in host *E. coli* normalized to inactive dBoNT/F control. The values and error bars represent mean ± s.d. of three replicates (**c**,**d**,**i**).

**Extended Data Fig. 8:**
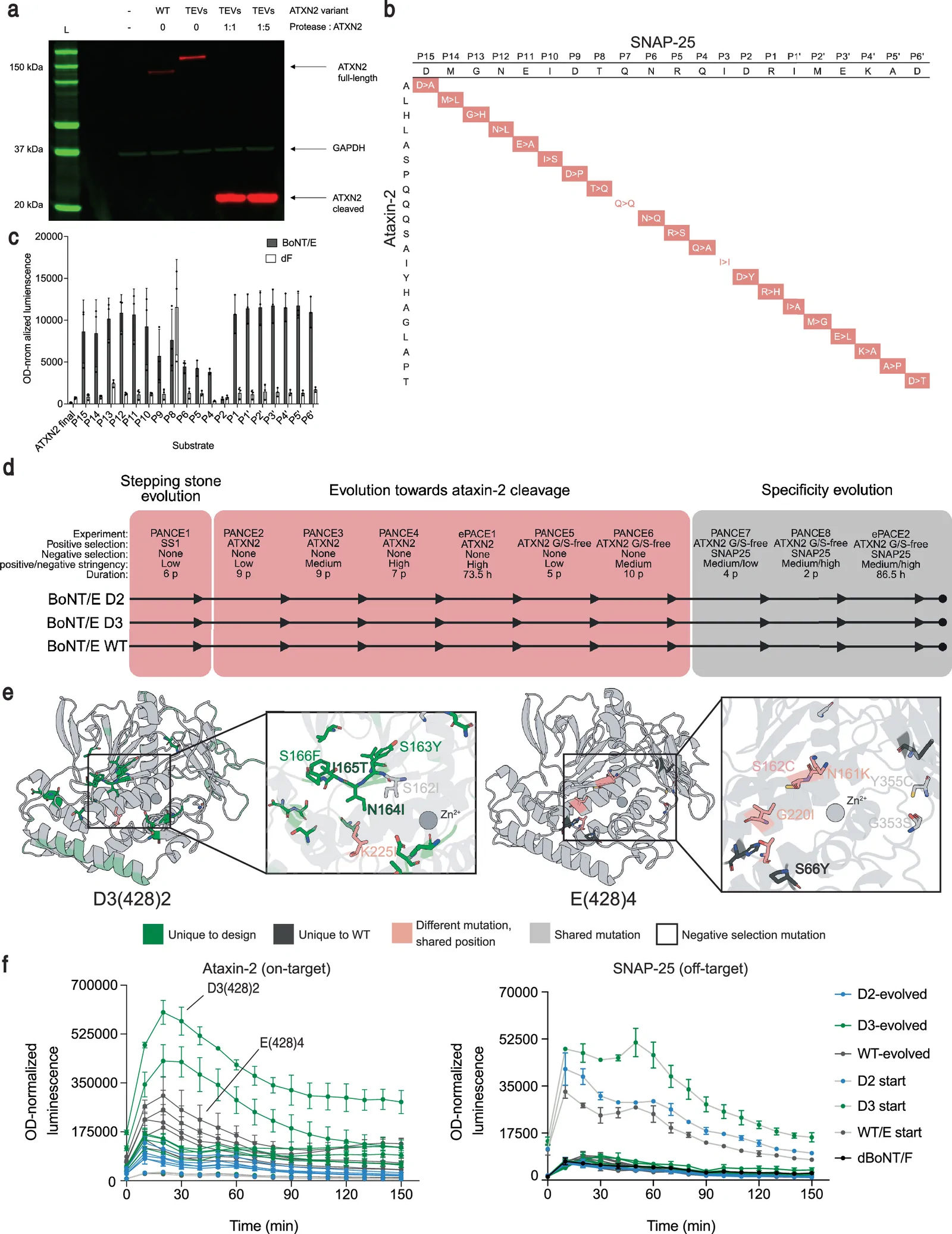
Validation of ataxin-2 as a protease evolution target, evolutionary trajectory, and initial assessment of ataxin-2-cleaving proteases. (**a**) Cleavage of TEV substrate-substituted ataxin-2 to assess if ataxin-2 is proteolytically accessible at the selected target site. Red, anti-FLAG chemiluminescence detection of the ataxin-2 substrate and cleavage product; green, 580 nm fluorescence detection of the GAPDH loading control. (**b**) Mutation at each substrate position that converts the SNAP-25 residue to the corresponding ataxin-2 target peptide residue in the alignment in Fig. 4b. (**c**) BoNT/E wild-type starting activity measured by luciferase signal on each single SNAP-25→ataxin-2 mutant substrate to identify the most challenging (poorly tolerated) single substitution. Catalytically dead BoNT/F (dF) used as a negative control. (**d**) Parallel evolutionary trajectories initiated with designed or wild-type BoNT/E towards specificity for ataxin-2 cleavage. Host selection schemes used for each stage described in Supplementary Fig. 6 and phage titers in Supplementary Fig. 7a–i. (**e**) Top-performing redesign-evolved and wild-type-evolved protease structures modelled in AlphaFold3 (refs. ^66–68^). MPNN-redesigned residues are in light green. (**f**). Apparent protease activity of consensus clones and starting proteases on positive selection substrate ataxin-2 (left) and negative selection substrate SNAP25 (right) measured by luciferase signal. Values and error bars in (**c**) represent mean ± s.d. of three replicates. Values and error bars in (**f**) represent mean ± s.d. of two replicates.

Having established enhanced evolution outcomes for sequence-redesigned proteases on substrates similar to the native substrate, we next applied this principle to reprogram a protease to a disease-relevant target unrelated to the native substrate while minimizing native substrate cleavage. We selected ataxin-2, a protein implicated in neurodegenerative disease that occurs in motor neurons, a cell type to which BoNT holotoxins natively self-deliver^42,50^. We chose to target ataxin-2 residues 1181–1201, which lie in the disordered IDR3 region that contributes to disease-associated condensation. Removal of this region of ataxin-2 is known to block the formation of aberrant aggregates^51^. We selected a target scissile bond in ataxin-2 that maintains a key protease–substrate residue contact and validated that this region is accessible to proteolysis (Fig. 6a, Extended Data Fig. 8a and Supplementary Note 3).

We performed parallel evolution campaigns initiated with redesigned BoNT/E variants D2 and D3, as well as with WT BoNT/E protease. The selections from each starting point were identical in all dilution factors, replicates and host selection stringencies, with two to four replicate selections per starting point at each stage (Extended Data Fig. 8d, Supplementary Fig. 6 and Supplementary Note 4). The 96-well plate format of PANCE and automated continuous culture in ePACE facilitated parallelization.

**Extended Data Fig. 9:**
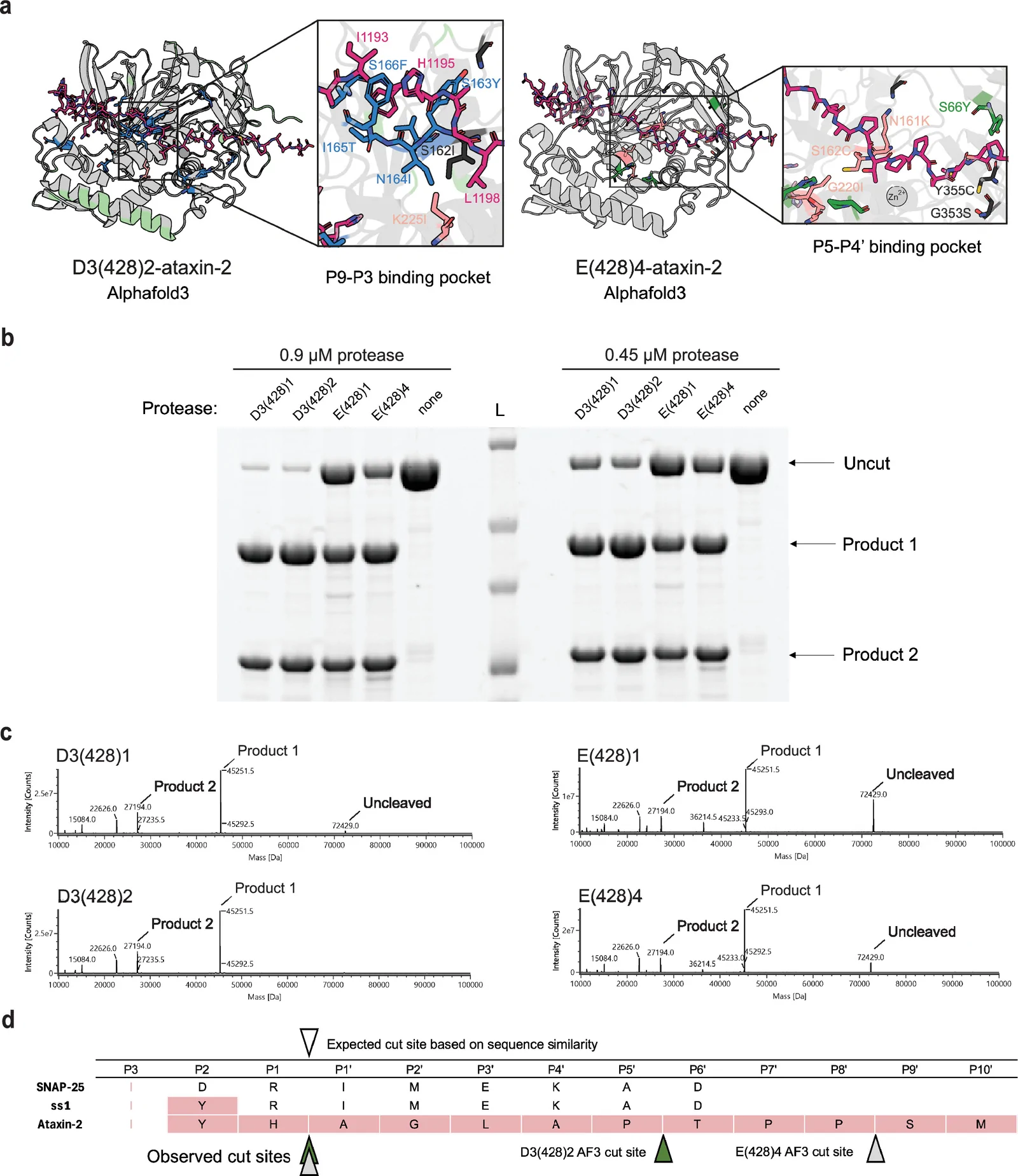
AlphaFold3 prediction and experimental determination of ataxin-2 scissile bonds for reprogrammed proteases. (**a**) AlphaFold3-predicted evolved protease-ataxin-2 complex structures highlighting predicted interactions between evolved residues and ataxin-2 substrate. (**b**) Gel shift assay for ataxin-2 substrate peptide cleavage. Cleavage reactions visualized on this gel were used for mass spectrometry analysis. (**c**) Mass spectra of 0.45 µM protease cleavage reactions for each of four evolved proteases. Peaks with masses consistent with gel band masses observed in b labeled as uncleaved substrate or product. (**d**) Observed cleavage sites in ataxin-2 that yield products with masses detected in c, compared to AlphaFold3-predicted cleavage sites.

Nanopore long-read sequencing of surviving phage clones identified functional consensus genotypes that differed between the redesigned and WT starting points, but were conserved within the lagoons of each starting point (Fig. 6b, Extended Data Fig. 9 and Supplementary Notes 5 and 6). Overall, evolution of sequence-designed proteases generated sequences highly divergent from the original enzyme found in nature: the top redesign-evolved protease has 46 mutations from ProteinMPNN and another 18 mutations from the full PANCE–PACE campaign, totalling 16% sequence difference from the BoNT/E protease. In comparison, the best WT-evolved protease evolved 10 mutations and is only 2.4% different from the natural enzyme.

## How redesign enhances ataxin-2 cleavage

**Extended Data Fig. 10:**
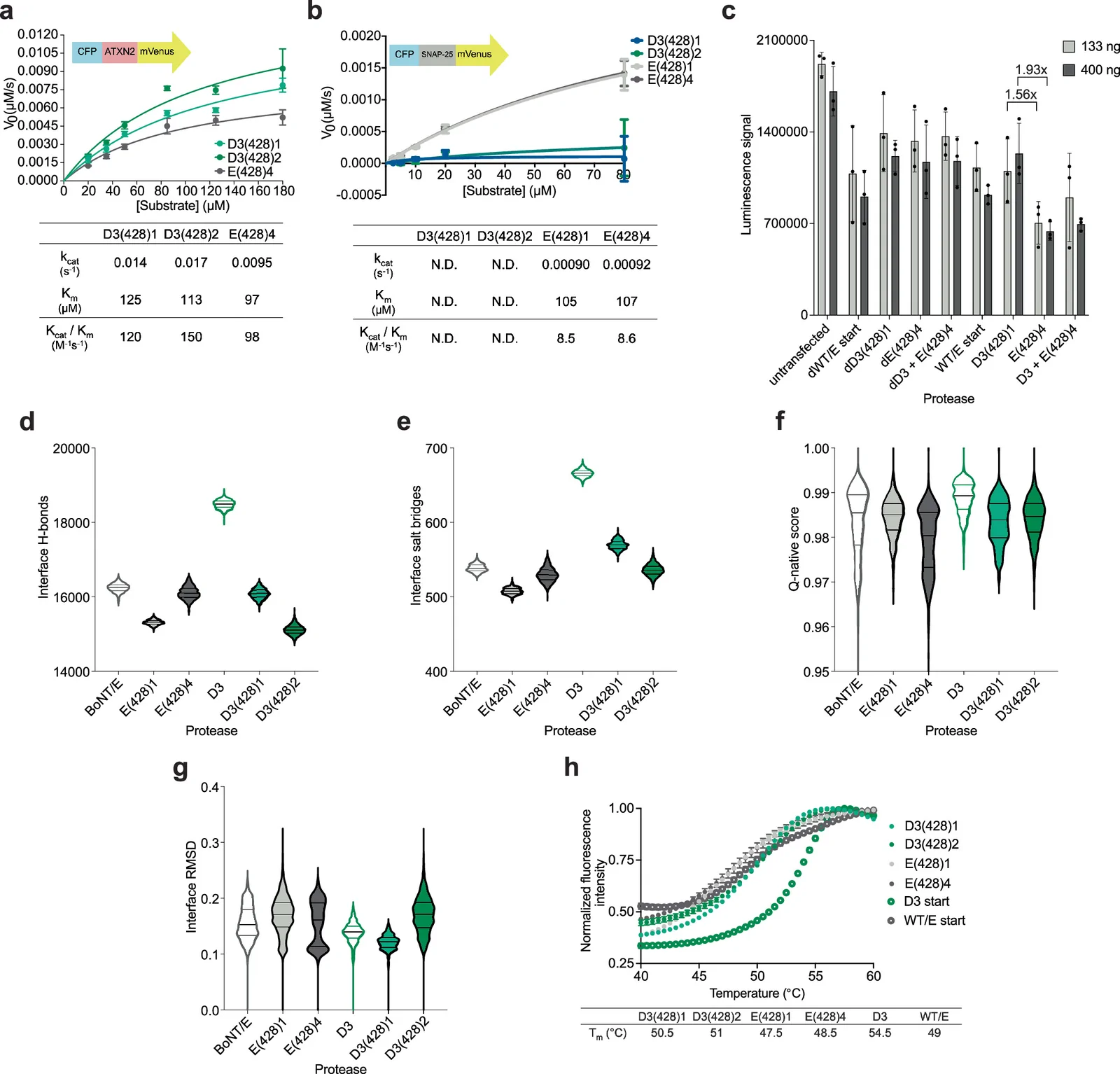
Biophysical analysis and toxicity of ataxin-2 cleaving proteases. (**a**) On-target ataxin-2 FRET substrate cleavage initial rate vs substrate concentration (top) and fitted parameters (bottom). (**b**) Off-target SNAP-25 FRET substrate cleavage initial rate vs substrate concentration (top) and fitted parameters (bottom). (**c**) Viability of HEK293T cells expressing ataxin-2-cleaving proteases measured by CellTiter-Glo assay. Cells were transfected with the indicated mass of protease expression plasmid and incubated for 42 h before measuring ATP-dependent luciferase activity. Values and error bars represent mean ± s.d. of three replicates. (**d to g**) Summary statistics for molecular dynamics simulations of protease-SNAP-25 complexes. The protease-substrate interface is defined as the pairs of inter-chain heavy atoms ≤ 4.5 Å apart. Q-native score is defined as the number of native protease-substrate contact pairs still within 4.5 Å at time t divided by the total number of native contact pairs defined in frame 0. (**h**) Thermal stability measured by SYPRO Orange fluorescence for highest-activity final ataxin-2-cleaving variants and unevolved starting points (top) and Tm values inferred as the temperature at which slope is maximal (bottom). Data are shown as mean ± s.d. for three replicates.

To understand whether evolving redesigned BoNT/E protease enabled improved the properties and applicability of proteases reprogrammed for ataxin-2 compared with starting with WT BoNT/E protease, we purified the best proteases from the redesigned and WT starting points and compared their kinetics, specificity and stability. Measuring on-target cleavage of ataxin-2 with sub-stoichiometric protease concentrations, we observed faster cleavage rates for both of the top two D3-evolved variants than the top two WT-evolved variants, in which D3(428)1 and D3(428)2 were 1.4-fold and 1.7-fold faster, respectively, than the best WT-evolved protease E(428)4 (Fig. 6c). When comparing off-target cleavage of SNAP25, WT-evolved proteases demonstrated detected cleavage rates that were 17,000-fold and 21,000-fold lower than the BoNT/E starting point, demonstrating the success of the dual selection circuit to enrich variants with diminished activity on the negative selection substrate (Fig. 6d). The D3-evolved ataxin-2 proteases showed no detected SNAP25 cleavage when assayed at the same protease concentration (Extended Data Fig. 10b).

To increase the sensitivity of the cleavage rate assay, we re-assayed SNAP25 cleavage with a super-stoichiometric protease concentration. Under these conditions D3(428)1 showed limited SNAP25 cleavage, whereas D3(428)2 still showed no detected cleavage rate, supporting the idea that this D3 variant may have achieved complete substrate reprogramming (Fig. 6d). The design-evolved proteases were quantified to have 79-fold and more than 56-fold greater selected specificity than E(428)4 (Fig. 6e,f).

We confirmed the high selected specificity of design-evolved proteases observed in kinetics time courses with alternative assays. We measured on-target and off-target protease activity with a gel shift assay. Off-target SNAP25 cleavage was observed for WT-evolved proteases but not for design-evolved proteases (Fig. 6g). Furthermore, a toxicity assay in human cells showed that the redesign-evolved D3(428)1 is less toxic than E(428)4, consistent with higher protease specificity that may reduce perturbations of cellular function (Extended Data Fig. 10c). Molecular dynamics simulations also corroborate the loss of SNAP25 activity after evolution, but did not explain differences between WT-evolved and redesign-evolved specificity (Extended Data Fig. 10d–g and Supplementary Note 7). Collectively, the on-target and off-target activity analysis demonstrates that the redesigned protease evolved solutions to the dual-selection challenge with superior specificity than those evolved from the WT.

Protein stability measurements revealed that the D3-evolved proteases were strongly destabilized compared with their starting point, but were still more stable than WT BoNT/E protease or WT-evolved variants. The WT-evolved proteases were also destabilized, although to a lesser degree, probably because their starting point had a smaller stability margin to trade for the evolution of new function (Extended Data Fig. 10h). We next assayed the expression and cleavage activity of the ataxin-2 proteases in mammalian cells. Co-transfection of plasmids expressing protease and ataxin-2 cDNA led to cleavage of ataxin-2 in HEK293T cells for all evolved proteases. Of note, the design-evolved protease showed higher expression than both the WT-evolved protease and the WT BoNT/E starting point, and the design-evolved protease yielded more efficient generation of the ataxin-2 cleavage product than the WT-evolved protease (Fig. 6h). We also found that the WT-evolved protease generated more abundant cleavage byproducts than a single, low-abundance byproduct for the D3-evolved protease, further suggesting enhanced selected specificity of the redesign-evolved protease (Supplementary Fig. 1h–i). Together, characterization and application of the ataxin-2-specific proteases demonstrate that evolution from redesigned proteases yielded solutions to the dual-selection challenge with higher expression, activity and specificity for a non-native target of therapeutic interest than evolution from WT enzymes.

Finally, we asked whether the high-specificity design-evolved genotypes were uniquely compatible with the redesigned protease background or could function in the WT background. Grafting all evolution mutations from D3(428)1 or D3(428)2 into the WT background resulted in no ataxin-2 cleavage activity (Fig. 6i). The WT background is therefore not robust to the evolution mutations that confer enhanced specificity, explaining why these genotypes did not arise in the WT BoNT/E-seeded lagoons. By contrast, grafting the evolution mutations from E(428)4 into the D3 background generated a protease with high activity and specificity for ataxin-2. Although D3(428)2 and E(428)4 showed similar selected specificity in this experiment (30.0-fold versus 30.2-fold), this similarity is probably an artefact of the inability of both proteases to cleave SNAP25 above that of the negative control. Consistent with these results in the context of the PACE circuit, the WT-background protease with D3-evolved mutations grafted had no observed activity in mammalian cells, whereas the D3-background protease with WT-evolved mutations grafted produced the target cleavage product (Fig. 6h). This striking distinction between the WT BoNT/E and D3 starting points is consistent with our findings from the three substrate variant evolutions, and strongly suggests that starting point redesign expands the productive mutation space of an evolving enzyme, enabling the emergence of new genotypes with virtually absolute specificity reprogramming not attained by evolving the natural enzyme.

## Discussion

Current computational protein design methodology is well suited for increasing stability, but the design of new function remains challenging. By contrast, experimental evolution excels at generating new function but often at the cost of reduced stability. Here we have shown that current computational protein design and laboratory evolution can together overcome both limitations to generate stable proteases with potent and specific new function. We restored the stability of a reprogrammed BoNT/E protease, and distinct sequence-redesigned BoNT proteases each consistently outperformed the natural protease at various new substrate selections, in all evolutions reaching high-activity mutation space that is non-functional in the undesigned natural enzyme. Our results reveal how redesign accelerates adaptation by supporting fitness in more local, rapidly accessible mutation space. Finally, the application of redesigned protein evolution developed a selection-specific and stable ataxin-2-cleaving protease with no detected native substrate cleavage, which was not achieved by evolving the starting point without redesign. These new design-evolved enzymes could enable the study of how disrupting pathogenic ataxin-2 inclusions at the protein level may rescue neurodegenerative disease.

Protease therapeutics are approved for various indications, although all current protease medicines target native substrates. Proteases evolved to cleave user-specified targets open new opportunities to exploit non-native cleavage events to control processes as important and diverse as blood coagulation, cell growth and differentiation, cell death and amyloid processing^52^. Our approach to increase the stability, activity and specificity of reprogrammed proteases may help to bring within reach the cleavage of many new targets for which a specific natural protease does not currently exist. The elimination of native substrate cleavage can be a critical property for the safety of a repurposed enzyme.

This work demonstrates how the intersection of computational protein redesign and automated continuous evolution can enable rapid, parallel enzyme reprogramming. The redesign and continuous evolution workflow developed here could in principle be applied to any protein that can be stabilized with sequence redesign and whose function can be selected in PACE, although the extent to which the consistent superiority of evolutionary outcomes from redesigned proteases observed in this study applies to other proteins will require future investigations. Relatedly, we have recently reported the ProteinMPNN redesign of PACE-evolved reverse transcriptases to improve intracellular levels and gene-editing efficiency of prime editors^53^. We recommend the redesign of stabilized starting points for laboratory evolution as a promising approach to generate enzymes with higher activity, specificity and stability than those evolved from natural proteins.

## Methods

### General methods

Antibiotics from Gold Biotechnology were prepared in 1,000× stock solutions in water unless otherwise indicated: chloramphenicol (25 mg ml^–1^ in 70% ethanol), carbenicillin (50 mg ml^−1^), spectinomycin (50 mg ml^−1^), tetracycline (10 mg ml^−1^ in 50% ethanol) and kanamycin (25 mg ml^–1^). PCR amplifications were carried out using either Phusion U Green Multiplex PCR Master Mix (Thermo Fisher Scientific) or Q5 Hot Start High-Fidelity 2× Master Mix (New England BioLabs). DNA oligonucleotides were synthesized by Integrated DNA Technologies. Plasmids encoding synthetic, human codon-optimized *ATXN2* cDNA sequences were obtained from GenScript. Plasmid constructs were assembled via Golden Gate or Gibson cloning protocols following previously described methods^21,37^. Cloning was performed in chemically competent *E. coli* Mach1 cells (Thermo Fisher Scientific) or NEB 5α cells (New England Biolabs). Plasmids from single colonies were amplified using the Illustra Templiphi 100 Amplification Kit (Cytiva) before sequencing via Sanger (Quintara Biosciences) or Nanopore (Quintara Biosciences or Plasmidsaurus). For bacterial experiments, plasmid DNA was purified using the QIAprep Spin Miniprep Kit (Qiagen), whereas plasmids for use in mammalian cells was isolated with the Plasmid Plus Midiprep Kit (Qiagen). All plasmids were eluted in nuclease-free water and quantified using a NanoDrop ONE UV-Vis spectrophotometer (Thermo Fisher Scientific). The list of plasmids and selection phages used in this study is available in Supplementary Table 1.

### ProteinMPNN sequence design

Code and procedure for sequence design are available on GitHub (https://github.com/Nicholas-Krasnow/sequence-design-guide). In summary, AF2-predicted structures of each redesigned BoNT protease were used as input for ProteinMPNN, and residues outside specified thresholds for distance to substrate and evolutionary conservation were constrained from redesign. AF2 predictions were confirmed to agree with existing experimental structures while offering a model of regions unresolved by crystallography^54–56^. Sequences were generated in groups according to the constraint cut-offs used. Substrate distance constraints were 14 Å and 18 Å for BoNT/E, 10 Å, 14 Å and 18 Å for BoNT/F, and 18 Å for BoNT/X as measured in PyRosetta^57^. Conservation of each residue was determined as the residue frequency in a multiple sequence alignment of homologues identified from a database search of Uniref50 with the starting BoNT protease as the search query. Conservation constraints of 30% and 60% were used for all proteases; each residue that was at least as conserved as the cut-off and was the plurality residue in the alignment position was constrained from design. For BoNT/X, an additional criterion was applied to constrain residues predicted to lie within 14 Å of the belt domain in the holotoxin based on alignment to the BoNT/A holotoxin structure (Protein Data Bank ID 3BTA)^58^. During sequence generation, two sampling temperatures of 0.1 and 0.3 were tested. Cysteine was excluded from design to avoid oxidation. Structures of output sequences were predicted in AF2 and evaluated by calculating the predicted local distance difference test score and root mean square deviation to the input structure measured in PyMOL.

### PROSS sequence design

PROSS variants of BoNT/E were generated using the PROSS webserver (https://pross.weizmann.ac.il/step/pross-terms/). The same AF2 input structure and the same distance constraints of 14 Å and 18 Å used for BoNT/E ProteinMPNN were used for PROSS. Because PROSS directly uses a multiple sequence alignment to sample putative stabilizing mutations, the conservation constraints used for ProteinMPNN were not applied. Folding energy was calculated with the ref2015 energy function. The webserver was run until 44 non-duplicate sequences were generated for each distance constraint group.

### Small-scale protein expression and purification

Starter cultures were grown overnight from single colonies and used to inoculate 5 ml of medium supplemented with 50 µg ml^−1^ kanamycin per well in a 24-well plate (50 μl inoculum per well). Cultures were incubated at 37 °C with shaking until reaching mid-log phase, then were subjected to a 1-h cold shock on ice. Protein expression was induced with 1 mM IPTG (Gold Biotechnology) and cultures were incubated overnight at 16 °C.

Overnight cultures were harvested by centrifugation. Cell pellets were resuspended in 400 μl of B-PER reagent (Thermo Fisher Scientific) supplemented with 0.8 μl each of lysozyme and DNaseI and 16 μl of cOmplete protease inhibitor tablet solution prepared in 2 ml nuclease-free water (Roche). Lysis proceeded by incubation at room temperature for 15 min without freeze–thaw cycles. Lysates were transferred to microcentrifuge tubes and clarified by centrifugation at 20,000*g* for 5 min at 4 °C. To prepare affinity resin plates for purification (HisPur Cobalt Spin Plates, Thermo Fisher Scientific), storage solution was removed from the resin by centrifugation at 500*g* for 3 min. Wells were washed once with 400 μl ultrapure water and centrifuged again at 500*g* for 3 min. Plates were equilibrated by two washes with 400 μl wash buffer (20 mM HEPES and 200 mM NaCl, pH 7.3), each followed by centrifugation. Plates were chilled on ice.

Following lysis, 300 µl of each supernatant (cleared lysate) was transferred to the pre-chilled 96-well cobalt resin plate and incubated for 15 min on ice. Plates were centrifuged for 3 min at 500*g* at 4 °C, and flow-through was collected. The resin was washed four times with 400 μl of wash buffer containing 10 mM imidazole, each wash followed by centrifugation at 500*g* for 3 min at 4 °C. Bound proteins were eluted by incubating the resin with 200 μl of elution buffer (wash buffer + 250 mM imidazole, pH 7.3) for 1 min, followed by centrifugation at 500*g* for 3 min at 4 °C. Eluates were sealed and stored at 4 °C.

To remove imidazole and adjust buffer conditions for downstream assay, buffer exchange was performed using Zeba spin desalting plates (Thermo Fisher Scientific). Desalting plates were equilibrated to room temperature, and storage buffer was removed by centrifugation at 1,000*g* for 2 min. Each well was washed four times with 250 μl exchange buffer (50 mM HEPES and 5 mM NaCl pH 7.3), each followed by centrifugation. Purified protein samples (100 μl) were applied to the resin bed, followed by 20 μl of exchange buffer to ensure full recovery. The processed protein was eluted by centrifugation at 1,000*g* for 2 min and collected in a new 96-well plate. SDS–PAGE analysis was used to assess protein expression and purification across various stages. Purified protein samples were stored at 4 °C for subsequent quantification and activity assays. Protein concentrations were measured using Pierce BCA assay (Thermo Fisher Scientific).

### Protease cleavage kinetics assay

FRET assays were conducted as previously described^59^. In brief, substrate proteins were diluted to the indicated concentrations in reaction buffer (50 mM HEPES pH 7.3, 5 mM NaCl, 2 mM dithiothreitol and 10 µM Zn(OAc)_2_). Proteases were diluted in reaction buffer to 20×, the indicated final concentration. Substrates (47.5 µl) were aliquoted into 96w white half-area, clear-bottom plates (Costar), and optimal gain values for 50% signal at 470 nm (donor fluorophore) and 100% signal at 526 nm were measured on a Tecan Spark plate reader. Diluted protease samples (2.5 µl) were added to substrate wells, mixed, then fluorescence emission was measured on the plate reader at 470 nm and 526 nm over a 1–2-h time course at 37 °C. After incubation, 1 µl trypsin (New England Biolabs) from a resuspension in 500 µl reaction buffer was added to each well, and emission at the same wavelengths at 37 °C was measured again until values plateaued. Conversion was calculated at each timepoint as previously reported according to the equation:

$${\rm{C}}{\rm{o}}{\rm{n}}{\rm{v}}{\rm{e}}{\rm{r}}{\rm{s}}{\rm{i}}{\rm{o}}{\rm{n}}={[S]}_{0}\times (X-{\rm{n}}{\rm{e}}{\rm{g}})/({\rm{p}}{\rm{o}}{\rm{s}}-{\rm{n}}{\rm{e}}{\rm{g}})$$

where [*S*]_0_ is the initial substrate concentration, *X* is the emission at 470 nm:emission at 526 nm (Em470:Em526) at a given timepoint, neg is the Em470:Em526 from protease-free substrate control at the same timepoint, and pos is the Em470:Em526 ratio upon complete conversion (trypsinolysis). Data were analysed using Microsoft Excel and subsequently plotted and fit using PRISM (GraphPad). Cleavage rate was calculated as the slope of linear fit to conversion over time in the linear range. When a statistically significant rate conversion (Pearson correlation *P* ≤ 0.05) was not observed, the maximum cleavage rate was calculated as the mean of three replicates plus three standard deviations.

### Medium-scale protein expression and purification

*E. coli* BL21 Star (DE3) cells (Thermo Fisher Scientific) transformed with plasmids encoding 6×His-tagged protease or FRET substrate constructs or MBP–GST substrate constructs were streaked onto kanamycin-containing agar plates, and single colonies were used to inoculate 2×YT cultures containing kanamycin (50 µg ml^−1^). Cultures were grown overnight at 37 °C with shaking at 220 rpm. The following day, overnight cultures were diluted 1:100 into fresh 2×YT medium (250–1,000 ml) containing kanamycin and incubated at 37 °C until reaching mid-log phase (optical density at 600 nm (OD_600_) ≈ 0.4–0.7). Cultures were then subjected to a 1-h cold shock on ice before induction with 1 mM IPTG (Gold Biotechnology). Induced cultures were incubated overnight at 16 °C with shaking.

Cells were harvested by centrifugation at 6,000*g* for 5 min and resuspended in cold lysis buffer (10 ml per 250 ml culture volume; 20 mM HEPES pH 7.3 and 200 mM NaCl, supplemented with cOmplete EDTA-free protease inhibitor tablet, Roche). Cell lysis was performed by sonication (amplitude 10, 3 s pulse on, 6 s pulse off, 6-min processing). The resulting lysate was clarified by centrifugation at 19,000*g* for 20 min at 4 °C.

For His-tagged proteins, the supernatant was loaded onto Ni-NTA affinity resin (Thermo Fisher Scientific; 1 ml resin per 250 ml culture), pre-equilibrated with wash buffer. Binding was carried out by gentle end-over-end mixing at 4 °C for 1 h. The resin was then transferred to gravity-flow columns and washed twice with 20 ml of cold wash buffer supplemented with 10 mM imidazole. Bound proteins were eluted in 4 × 1.5 ml fractions using wash buffer + 250 ml imidazole, pH 7.3. For MBP–GST substrate proteins, supernatants were instead loaded onto glutathione agarose resin (Thermo Fisher Scientific), washed twice with wash buffer, then eluted with wash buffer + 50 mM glutathione. Eluted fractions were evaluated by SDS–PAGE, and those containing the target protease were pooled and subjected to buffer exchange with Amicon 15-kDa centrifugal filters (Millipore Sigma) into the original wash buffer. Final protein concentrations were determined using the BCA assay. For long-term storage, proteins were combined with an equal volume of 20% glycerol dissolved in wash buffer, flash-frozen with liquid nitrogen, then stored at −80 °C.

### Thermal stability assay

Thermal stability was determined by measuring *T*_m_ values according to a published protocol^60^. Proteins were diluted in reaction buffer to 1 µg µl^−1^. In a PCR plate, 1 µl diluted protein samples or 1 µl buffer-only blank samples were then added to 19 µl dye solution comprising reaction buffer with 800×-diluted SYPRO orange (Thermo Fisher Scientific) and mixed. Melting was observed by measuring fluorescence on a Bio-Rad quantitative PCR machine with the following protocol: equilibrate to 25 °C for 2 min, increase temperature by 0.5 °C, then equilibrate for 1 min and measure fluorescence at 560 nm, repeating the cycle until reaching 95 °C. Fluorescence versus temperature and the first derivative versus temperature were plotted, and *T*_m_ values were identified as the temperature of the maximum derivative value.

### Preparation and transformation of chemically competent cells

Strain S20607 was used for all luciferase assays, phage amplification, plaque formation assays and PACE experiments. Chemically competent cells were prepared by diluting overnight cultures 1:50 into 50 ml of 2×YT medium supplemented with tetracycline. Cultures were grown at 37 °C with shaking (220 rpm) until reaching an OD_600_ of 0.4–0.6. Cells were harvested by centrifugation at 4,000*g* for 10 min at 4 °C and gently resuspended in 5 ml of TSS buffer (LB containing 5% v/v DMSO, 10% w/v PEG 3350 and 20 mM MgCl). Aliquots were frozen on dry ice and stored at −80 °C for later use.

For transformation, 100 µl of thawed competent cells was added to a chilled mixture of plasmid DNA (1–2 µl per plasmid, up to three plasmids total) and 95 µl of KCM buffer (100 mM KCl, 30 mM CaCl_2_ and 50 mM MgCl_2_ in water). The mixture was gently stirred and incubated on ice for up to 10 min, followed by a 75-s heat shock at 42 °C. SOC medium (500 µl; New England Biolabs) was then added, and cells were recovered at 37 °C with shaking for 1 h. Transformed cells were plated on 2×YT agar containing the appropriate antibiotics and incubated at 37 °C for 16–18 h.

### Luciferase assay for PACE circuit activation

S2060 cells were transformed with the relevant substrate accessory plasmid and pBAD protease expression plasmid. Single colonies were cultured overnight in 2×YT medium supplemented with the appropriate maintenance antibiotics. The next day, cultures were diluted 1:50 into fresh DRM medium containing the same antibiotics and grown at 37 °C with shaking (220 rpm) until reaching an OD_600_ of 0.4–0.6. Arabinose (1 mM) was added to each sample to induce protease expression, 100 µl of each sample was transferred into a black-walled, flat clear-bottom 96-well plate (Costar) for measurement. Wells containing DRM only were included to obtain background measurements. Absorbance at 600 nm and luminescence were recorded using either a Tecan Infinite M1000 Pro or Tecan Spark microplate reader over a time course of 2.5 h. Luminescence readings were normalized to cell density by dividing the background-subtracted luminescence signal by the background-subtracted OD_600_ absorbance.

### Toxin purification and luciferase complementation assay for toxin delivery

Plasmids encoding each toxin, tagged with a C-terminal 6×His sequence, were transformed into BL21 DE3 competent cells (New England Biolabs). The transformed cells were plated on LB agar containing 50 µg ml^−1^ kanamycin and incubated overnight at 37 °C. A single colony was selected and cultured overnight in 2×YT medium supplemented with 50 µg ml^−1^ kanamycin at 37 °C. The following day, this culture was diluted 1:100 into fresh 2×YT medium and incubated at 37 °C with shaking (200 rpm) until the OD_600_ absorbance reached approximately 0.6. At this point, cultures were subjected to a 20-min cold shock, after which protein expression was induced by adding 1 mM IPTG. The cultures were then incubated overnight with shaking at 16 °C.

Protein purification was performed at 4 °C. Cells were harvested via centrifugation at 4,000 rpm for 5 min and resuspended in lysis buffer (20 mM Tris-HCl, 500 mM NaCl, 1 mM MgCl_2_, 10 mM imidazole, 10% glycerol, 1 mM PMSF, protease inhibitor cocktail, 100 µM ZnCl_2_ and 100 mM arginine, pH 7.5). Lysis was achieved by sonication (3 s pulse on, 6 s pulse off, 3-min processing). The lysate was clarified by centrifugation at 18,000*g*, and the supernatant was applied to a Ni-NTA affinity column. The column was washed twice using a wash buffer containing 20 mM Tris-HCl, 500 mM NaCl, 50 mM imidazole, 1 mM MgCl_2_, 100 µM ZnCl_2_, 10% glycerol and 100 mM arginine (pH 7.4). Proteins were eluted with elution buffer (20 mM Tris-HCl, 500 mM NaCl, 1 mM MgCl_2_, 250 mM imidazole, 10% glycerol, 100 µM ZnCl_2_ and 100 mM arginine, pH 7.4). Eluted proteins were first concentrated using a 15-ml Amicon Ultra centrifugal filter (100,000 kDa cut-off) at 3,500*g* and buffer exchanged into 20 mM Tris-HCl, 150 mM NaCl, 100 µM ZnCl_2_ and 10% glycerol, pH 7.5. A second concentration step was performed using a 0.5-ml Amicon Ultra filter (100 kDa cut-off) at 14,000 *g*. Protein concentrations were measured with the BCA assay, and samples were treated with 10 U mg^−1^ thrombin at 4 °C overnight.

For HiBiT:LgBiT luciferase live-cell complementation assays, 15 K N2A cells were seeded in 96-well plates and transfected with 33 ng per well pLX304_CMV::mCherry-LgBiT-FLAG-NES (Addgene #199715) 24 h after seeding. After another 24 h, the media were replaced with 100 µl of complete medium containing 1 µM HiBiT-tagged toxin or HiBiT peptide. Following another 24-h incubation, cells were washed with PBS and bioluminescence was measured using the Nano-Glo Live Cell Assay System (Promega), according to the manufacturer’s instructions, using a Tecan Spark plate reader. In parallel, 50 µl of media was collected separately from the plate after treatment with Nano-Glo substrate and used to assess background signal. This background reading was subtracted from the luminescence values obtained from the treated cells.

### PTEN cleavage assay in mammalian cells

Of HEK293T cells (American Type Culture Collection (ATCC), mycoplasma free) at 160,000 cells per millilitre, 2.7 ml was seeded in six-well culture plates. After 24 h, wells were co-transfected with 1.1 µg full-length FLAG-tagged PTEN in pCDNA3.1 vector (Addgene #22231) and 1.1 µg N-terminal OSBP pleckstrin-homology domain tagged (to promote colocalization with PTEN), C-terminal 6×His-tagged proteases in pCDNA3.1 using Lipofectamine 2000 transfection reagent (Thermo Fisher Scientific) following the manufacturer’s instructions. For no protease negative controls, 1.1 µg pUC19 plasmid was transfected instead of protease expression plasmid. Cell lysates were harvested 24 h later in lysis buffer (RIPA (Thermo Fisher Scientific) with 1 mM PMSF and cOmplete protease inhibitor (Roche)). The lysates were analysed by western blot for PTEN (using an anti-FLAG tag antibody) or protease (using an anti-6×His tag antibody) as well as GAPDH as a loading control. Anti-FLAG and anti-6×His were detected with horseradish peroxidase-conjugated secondary antibodies and chemiluminescence measurement, whereas anti-GAPDH was detected with IRDye 680RD-conjugated secondary antibodies and fluorescence measurement. Band intensity was quantified with Image Lab (Bio-Rad).

### Substrate profiling assay

A single-stranded DNA oligo library of the SNAP25 sequence from D166 to D186 with every possible single-residue substitution was synthesized from Twist Biosciences. PCR was used to amplify the oligo library into double-stranded DNA, which was purified by PCR cleanup (Qiagen). A T7 substrate plasmid backbone was amplified by PCR, DpnI digested to remove template plasmids and purified by PCR cleanup. The double-stranded substrate library was cloned into the plasmid backbone by isothermal assembly (NEBuilder-HiFi, New England Biolabs), and the resulting plasmid library (t7s) was purified by ethanol precipitation using GlycoBlue coprecipitant (Thermo Fisher). Purified plasmid was electroporated into NEB-10β electrocompetent *E. coli* (New England Biolabs), recovered in 20 ml 2×YT medium supplemented with 25 mM glucose for 10 min, and transferred into 200 ml terrific broth supplemented with 50 µg ml^−1^ carbenicillin for overnight recovery at 37 °C in a 220-rpm shaking incubator. The cloned plasmid library was harvested from NEB-10β outgrowth culture via midiprep (Qiagen), following the manufacturer’s protocols. The plasmid library was subsequently electroporated into S2060 containing pBAD-BoNT/E plasmid, recovered in 20 ml 2×YT medium supplemented with 25 mM glucose for 10 min, and transferred into 200 ml terrific broth supplemented with 50 µg ml^−1^ carbenicillin and 50 µg ml^−1^ spectinomycin for overnight recovery at 37 °C in a 220-rpm shaking incubator.

To perform selection, 1 ml of S2060 [pBAD-BoNT/E] [t7s] library culture was pelleted and resuspended in 1 ml of DRM to remove residual growth medium. The washed cell suspension was inoculated into 49 ml pre-warmed DRM supplemented with carbenicillin, spectinomycin and 20 µM arabinose in a disposable 125-ml bacterial culture flask. The flask was incubated at 37 °C with 220 rpm shaking to induce protease expression. Once the cells reached an OD_600_ of 0.8, 1 ml of culture was inoculated into 49 ml DRM with carbenicillin, spectinomycin and arabinose supplemented with 25 µg ml^−1^ chloramphenicol. Of induced culture, 1 ml was also inoculated into 49 ml DRM–carbenicillin–spectinomycin–arabinose without chloramphenicol. Selection cultures were incubated overnight at 37 °C with 220 rpm shaking. Following overnight selection, plasmids from both the plus and the minus chloramphenicol cultures were harvested by midiprep (Qiagen), following the manufacturer’s protocols. The substrate sequences from the plasmid pools of both cultures were amplified by PCR using primers rCFH0600 and rCFH0601 and barcoded using the Illumina Truseq adaptor system. The substrate sequences of the plasmid pools were sequenced on an Illumina MiSeq (Illumina). Sequencing data were analysed using custom scripts to count the occurrence of each substrate sequence within both the plus and the minus chloramphenicol conditions. For each substrate *s*, the log-enrichment score E_s_ was calculated using the following formula:

$${{\rm{E}}}_{{\rm{s}}}={\log }_{2}\,\left(\frac{\left(\frac{{C}_{s,+}}{\sum _{S}{C}_{s,+}}\right)}{\left(\frac{{C}_{s,-}}{\sum _{S}{C}_{s,-}}\right)}\right)$$

where *C*_*s*,+_ is the read count of substrate *s* in the plus chloramphenicol condition and *C*_*s*,–_ is the read count of substrate *s* in the minus chloramphenicol condition. Raw read counts were normalized to the total number of reads across all substrates in each condition to account for differences in sequencing yield.

### Plaque assay

S2060 cells were transformed with pJC175e (Addgene #79219) to generate S2208 (ref. ^8^). Overnight cultures of single S2208 colonies grown in 2×YT medium supplemented with carbenicillin and tetracycline were diluted 50-fold into fresh DRM with the same antibiotics and grown at 37 °C with shaking at 220 rpm to OD_600_ ~ 0.6–0.8. Phage were serially diluted 100-fold (4 dilutions total) in DRM. Of cells, 100 µl was added to 10 µl of each phage dilution. Of top agar pre-warmed to 55 °C (LB medium + 0.33% LB agar), 500 µl was added and mixed by pipetting up and down once. These mixtures were then immediately pipetted onto 24-well plate wells containing solidified bottom agar (LB agar with 0.04% Bluo-Gal (Research Products International), no antibiotics). After solidification of the top agar, plates were incubated at 37 °C for 16–18 h. Phage titres were determined by counting plaques in dilution wells with approximately 10–100 plaques.

### Overnight propagation assays

Host cells were prepared by transforming S2060 cells with accessory plasmid, and overnight cultures of single colonies were grown overnight in 2×YT medium supplemented with antibiotics. Cultures were diluted 50-fold into fresh DRM with antibiotics and grown at 37 °C with shaking at 220 rpm to OD_600_ ~ 0.6–0.8. Host cells were infected with phage at a titre of 1 × 10^5^ PFU ml^−1^ then grown at 37 °C with shaking at 220 rpm overnight. Phage were collected by centrifugation at 8,000*g* for 2 min. Titres of propagated phage were determined by plaque assay, and overnight propagation values were calculated as output phage titre/input phage titre.

### General method for eVOLVER-enabled phage-assisted continuous evolution

PACE host strains, phage, turbidostats, lagoons and media were prepared as previously described^2,5,6,11^. Hardware and software for autonomous eVOLVER and min-eVOLVER devices were used as reported for these systems^45,47^. For comparison evolutions, to prevent differences in mutation profiles occurring due to differences in codon usage, phage-encoded proteases were designed so that the same codon was used between the WT and designed starting points at all synonymous positions.

Hosts were prepared by transforming chemically competent S2060s with AP(s) and MP6, plated on 2×YT agar with antibiotics and 100 mM glucose to suppress MP6 expression, then grown at 37 °C for 16–18 h. Colonies were picked into cultures of DRM with antibiotics, and a dilution series of six 10-fold dilutions of each culture was prepared and grown with shaking at 37 °C for 16–18 h. Cultures with OD_600_ ~ 0.4–0.8 were used to inoculate 30 ml eVOLVER or min-eVOLVER reservoirs to serve as turbidostats. Turbidostats were set to maintain mid-log OD_600_ of 0.6–0.8 at constant volume. Phage were subjected to evolutionary drift before PACE by preparing a drift host (S2208 transformed with MP6), infecting mid-log culture of drift host with phage, then collecting phage by centrifugation at 8,000*g* for 2 min after growing 12–16 h at 37 °C.

Lagoons were maintained at a volume of 5 ml with the indicated influx flow rate of host culture from the turbidostat. Mutagenesis was induced by pumping 250 mM arabinose into the lagoon to a constant final concentration of 10 mM. Lagoons were infected with phage by inoculating 500 µl phage at the desired titre into each lagoon. Samples (500 µl) of the phage population were taken at indicated timepoints from the lagoon waste needle. Timepoint samples were centrifuged at 8,000*g* for 2 min and phage-containing supernatants were stored at 4 °C. Lagoon titres were determined by plaque assay of phage samples. For sequencing of phage clones, eight single plaques were picked, and the transgene region was amplified with PCR and sequenced by Nanopore (Quintara Biosciences or Plasmidsaurus). Consensus clones were defined as the genotypes comprised each observed combination of recurring mutations.

### General method for PANCE

Hosts and phage were prepared as described above for PACE. Cultures of host cells containing MP6 at OD_600_ ~ 0.4–0.8 were supplemented with 20 mM arabinose to induce mutagenesis then aliquoted into 1 ml cultures in a 96 deep-well plate. Cultures were inoculated with selection phage at the indicated dilution, then grown 12–16 h at 37 °C and harvested the next day by centrifugation (4,000*g* for 10 min) and storage of supernatant at 4 °C. Phage were then used to infect the subsequent PANCE passage with hosts prepared the same way. The process was repeated for the number of passages indicated, with titres determined by plaque assay after each passage. Drift passages were performed as described for PACE.

### LoopSeq

Phage pools from PACE were prepared by propagating phage with S2208 host cells overnight followed by PCR amplification of phage pools using phage transgene PCR primers used for plaque sequencing. A low cycle count of 6–14 cycles was used to reduce the frequency of recombination during PCR. Amplicon pools were purified by Ampure XP bead purification and size purity was confirmed by TapeStation. Sequencing of prepared samples and in silico assembly of synthetic long reads was performed at Element Biosciences. Long reads were analysed by aligning reads to the starting protease gene as the reference, determining the coding mutations compared with the reference, and counting the number of reads of each observed mutation combination. Abundant genotypes were defined as those with more than 100 assembled long reads mapped, which were plotted in histograms and included in mutation set sharing, entropy and Hamming distance analyses. Mutation combinations arising from a particular starting point were classified as shared if the same mutation combination occurred in at least one read of at least one replicate of the alternative starting point, and mutation combinations were classified as starting point specific if not.

### Analysis of protein sequence embeddings in ESM-C

Sequence embeddings of evolved consensus genotypes in the WT BoNT/E background were generated according to the ESM repository embedding tutorial (https://github.com/evolutionaryscale/esm/tree/main/cookbook/tutorials). Principal component analysis was performed on each embedding layer, then the embedding layer with principal components 1 and 2 that clustered evolved protease sequences by substrate closest to the *k*-means clusters, according to Rand index, was selected for analysis (layer 5). The BoNT/E background mutants were plotted in the principal component space and the mutants were replotted with the same coordinates to show activity in the D3, D4 and PROSS1 backgrounds.

### Gel shift proteolysis assays

Substrate proteins were diluted to the indicated concentration in reaction buffer. Proteases were diluted in reaction buffer to 20× the indicated final concentration. Of diluted protease samples or buffer-only negative controls, 2.5 µl was added to diluted substrates in PCR strip tubes, mixed, then incubated for the indicated reaction time at 37 °C. Reactions were quenched in LDS loading buffer supplemented with 2 mM dithiothreitol, then analysed by SDS–PAGE and Coomassie InstantBlue stain (Abcam).

### Intact protein mass spectrometry

Proteolysis reactions were diluted to 100 ng μl^−1^ in 25 mM HEPES pH 7.5, 10 mM MgCl_2_ and 150 mM NaCl. Of diluted protein, 1 μl was injected onto a Waters BioAccord LC-TOF (composed of an ACQUITY I-Class UPLC and RDa detector with an ESI source) with an ACQUITY UPLC Protein BEH C4 column (300 Å, 1.7 μm, 2.1 × 50 mm) at 80 °C for the duration of analysis. Samples were desalted for 1 min before being eluted onto the mass spectrometer with a 5–85% gradient of acetonitrile in 2.5 min at a flow rate of 0.4 ml min^−1^. Ionization was performed at a cone voltage of 55 V and desolvation temperature of 550 °C. The instrument scanned at a rate of 0.2 scans per second over the range 50–2,000 *m*/*z*. Protein *m*/*z* spectra were deconvoluted into intact mass using the MaxEnt1 function within UNIFI software (Waters).

### Molecular dynamics simulations of protease–substrate complexes

WT BoNT/E and D3 protease–substrate complex structures from which simulations were initiated from were prepared by AF3 prediction of the protease with Zn^2+^ and the following SNAP25 cleavage site-flanking subsequence:

MDENLEQVSGIIGNLRHMALDMGNEIDTQNRQIDRIMEKADSNKTRIDEANQRATKMLG.

Because AF3 accurately placed the substrate in the correct register for the highly active SNAP25-cleaving proteases WT BoNT/E and D3, but not for the proteases evolved not to cleave SNAP25, evolved protease–substrate complexes were instead prepared by mutating the parent WT or D3 complex prediction to the evolved sequence.

All-atom molecular dynamics simulations were performed using OpenMM (v8.2)^61^. Protein systems were parameterized with the Amber ff14SB force field^62^, and the catalytic Zn^2+^ ion was treated using the standard Amber non-bonded 12-6 model^63^. Existing hydrogen atoms were removed from input structures and re-added at pH 7.0 to ensure consistent protonation states. Each system was solvated in a rectangular box of TIP3P water^64^ with a minimum padding of 10 Å between the solute and box edges. No counterions were added to preserve the native electrostatic environment of the peptide–Zn^2+^ complexes. Long-range electrostatic interactions were calculated using the particle mesh Ewald method^65^, and all bonds involving hydrogen atoms were constrained using the SHAKE algorithm, permitting a 2-fs integration timestep.

Simulations were conducted in the canonical (NVT) ensemble at 298 K using a Langevin integrator with a friction coefficient of 1.0 ps^−1^. Before production dynamics, each system underwent 500 steps of energy minimization to relieve steric clashes. Production trajectories were propagated for 3 μs per system (1.5 × 10^9^ integration steps), with coordinates saved every 100 ps for subsequent analysis. To reduce storage requirements, only protein and Zn^2+^ ion coordinates were retained in trajectory files, excluding bulk solvent. All simulations were performed on NVIDIA L40s and H100 GPUs, hosted on the MIT Engaging cluster administered by MIT Research Computing, using mixed-precision arithmetic. Checkpoint files were written every 50 ps to enable seamless restart upon computational resource preemption, ensuring continuous trajectory accumulation across multiple job submissions.

### Ataxin-2 protease expression and cleavage assay in mammalian cells

Of HEK293T cells (ATCC) at 160,000 cells per millilitre, 2.7 ml was seeded in six-well culture plates. After 24 h, wells were co-transfected with 1.1 µg full-length FLAG-tagged *ATXN2* cDNA in a pCDNA3.1 vector and C-terminal 6×His-tagged proteases in pCDNA3.1 using Lipofectamine 2000 transfection reagent (Thermo Fisher Scientific) following the manufacturer’s instructions. For protease expression blots, 1.1 µg protease plasmid was transfected, whereas for cleavage detection blots 0.11 µg was transfected. For no protease negative controls, an equivalent mass of pUC19 plasmid was transfected instead of protease expression plasmid. Cell lysates were harvested 24 h later in lysis buffer (RIPA (Thermo Fisher Scientific) with 1 mM PMSF and cOmplete protease inhibitor (Roche)). The lysates were analysed by western blot for ataxin-2 (using an anti-FLAG tag antibody) or protease (using an anti-6×His tag antibody) as well as GAPDH as a loading control. Anti-FLAG and anti-6×His were detected with horseradish peroxidase-conjugated secondary antibodies and chemiluminescence measurement, whereas anti-GAPDH was detected with IRDye 680RD-conjugated secondary antibodies and fluorescence measurement. Band intensity was quantified with Image Lab (Bio-Rad).

### Mammalian cell viability assay to measure protease toxicity

Of HEK293T cells (ATCC) at 160,000 cells per millilitre, 100 µl was seeded in 96-well culture plates. After 24 h, wells were transfected with the indicated mass of indicated protease expression plasmid using Lipofectamine 2000 transfection reagent (Thermo Fisher Scientific) following the manufacturer’s instructions. Viability was measured 42 h post-transfection with the CellTiter-Glo kit (Promega) according to the manufacturer’s instructions. Cells were incubated at room temperature for 30 min before 100 µl CellTiter-Glo reagent was added to each well. Contents were mixed, signal was allowed to stabilize for 10 min, then luminescence dependent on cellular ATP was measured using a Tecan Spark plate reader.

### Reporting summary

Further information on research design is available in the Nature Portfolio Reporting Summary linked to this article.

## Online content

Any methods, additional references, Nature Portfolio reporting summaries, source data, extended data, supplementary information, acknowledgements, peer review information; details of author contributions and competing interests; and statements of data and code availability are available at https://doi.org/10.1038/s41586-026-10820-0.

## Supplementary information

Supplementary Information Supplementary Notes 1–7 and Supplementary Figures 1–8

Reporting Summary

Supplementary Tables Supplementary Tables 1–13

Peer Review file

## Data Availability

The raw data are provided in the Supplementary Tables, and uncropped western blots are provided in Supplementary Information.

## Code availability

Codes for the protease sequence design with ProteinMPNN (https://github.com/Nicholas-Krasnow/sequence-design-guide) and the LoopSeq analysis (https://github.com/Nicholas-Krasnow/LoopSeq) are available on GitHub.
