# Supplementary material for: AI-redesigned starting points and outcomes enhance protein evolution
Source: Nature. 2026 Jul 22;657(8131):525–38. doi: 10.1038/s41586-026-10820-0 (PMC13477572; doi:10.1038/s41586-026-10820-0)
Supplement: Supplementary file 1 [file 41586_2026_10820_MOESM1_ESM.pdf]

nature portfolio

https://doi.org/10.1038/s41586-026-10820-0

# Supplementary information

# AI-redesigned starting points and outcomes enhance protein evolution

In the format provided by the authors and unedited

Supplementary Information

# AI-redesigned starting points and outcomes enhance protein evolution

Nicholas A. Krasnow, Joy A. Xu, Emily Zhang, Gandhar K. Mahadeshwar, Y. Allen Tao, Julia McCreary, Colin F. Hemez, Logan E. Brown, Wei Jiang, and David R. Liu

## Supplementary Notes

Supplementary Note 1. Sequence redesign of BoNT/F and BoNT/X proteases with ProteinMPNN.
Supplementary Note 2. Prediction of evolvability from redesign metrics.
Supplementary Note 3. Identification of an ataxin-2 target peptide.

Supplementary Note 4. Evolutionary trajectory for the ataxin-2 cleavage campaign.

Supplementary Note 5. Observations of ataxin-2-evolved protease genotypes.

Supplementary Note 6. Alphafold3 prediction and mass spectrometry determination of ataxin-2 protease cleavage sites.
Supplementary Note 7. Molecular dynamics simulations of protease•SNAP-25 complexes.

## Supplementary Figures

Supplementary Figure 1. Uncropped PAGE gels and western blots.

Supplementary Figure 2. Properties of redesigned protease mutations for each BoNT serotype and redesign method.

Supplementary Figure 3. Frequency of redesign mutations in multiple sequence alignments (MSA)s by serotype and redesign method.

Supplementary Figure 4. Thermal stability of selected proteases from the comparison evolution panel evolved from wild-type or redesigned starting points and their grafts.

Supplementary Figure 5. Catalytic activity and stability of the alternative ProteinMPNN redesign D4.

Supplementary Figure 6. PANCE and PACE hosts by positive and negative substrates used over the evolutionary trajectory to evolve ataxin-2-specific proteases overviewed in Extended Data Fig. 8d.

Supplementary Figure 7. Phage titers over each stage of the ataxin-2 protease evolution overviewed in Extended Data Fig. 8d and intermediate characterization.

Supplementary Figure 8. Thermal stability of D2-evolved ataxin-2-cleaving proteases in comparison to wild-type-evolved proteases.

**Supplementary Tables (provided as sheets in a separate Excel file)**

Supplementary Table 1. Plasmid and phage genome sequences.

Supplementary Table 2. Primer sequences.

Supplementary Table 3. ProteinMPNN and PROSS redesign sequences and assay results plotted in Fig. 1c, Extended Data Fig. 2b, and Extended Data Fig. 3, b and g.

Supplementary Table 4. Statistics of redesign mutation structural features plotted in Supplementary Figure 2.

Supplementary Table 5. Melting temperatures derived from Fig. 1e, Fig. 2c, and Supplementary Fig. 4c.

Supplementary Table 6. Michaelis-Menten kinetic parameters derived from Fig. 1f and Fig. 2d.

Supplementary Table 7. PTEN-cleaving proteases used in Fig. 2.

Supplementary Table 8. Success rate and adaptation time for PACE experiments in Fig. 3.

Supplementary Table 9. Raw data for comparison evolution variant characterization in Fig. 3, a to c, bottom panels.

Supplementary Table 10. Raw data for the activity of consensus genotypes in all starting point backgrounds on the comparison evolution panel of substrates in Fig. 5.

Supplementary Table 11. Fraction of evolved consensus genotypes active in each starting point background on the comparison evolution panel of substrates in Extended Data Fig. 6.

Supplementary Table 12. Consensus genotypes evolved from the ataxin-2 cleavage evolution campaign outlined in Fig. 4, a to c.

Supplementary Table 13. Ataxin-2-cleaving protease sequences used in Fig. 4 and Fig. 5.

**Supplementary Note 1. Sequence redesign of BoNT/F and BoNT/X proteases with ProteinMPNN.**

To redesign BoNT/F protease, we used ProteinMPNN to generate sequences with binding pocket distance cutoffs of 10 Å, 14 Å, and 18 Å, and conservation cutoffs of 30% and 60% (**Extended Data Fig. 3a**). We generated 14 designs per constraint group and experimentally assayed 81 total variants. Sixteen (20%) of these designs were active proteases, six (7.4%) showed activity at least that of the wild-type, and all six of these variants offered higher soluble yield than wild-type BoNT/F protease (**Extended Data Fig. 3b**).

For BoNT/X protease, we set a single binding pocket constraint cutoff of 18 Å as this distance yielded all of the best variants for BoNT/E and BoNT/F, and used the 30% and 60% conservation cutoffs. Because we previously showed that evolved BoNT/X proteases can be compatible with the toxin heavy chain module for intracellular delivery (*16*), we tested an additional criterion in which protease residues that contact the belt domain of the heavy chain and may contribute to assembly of the delivery-capable holotoxin were constrained from redesign (*46*, *47*) (**Extended Data Fig. 3f**). We generated 22 redesigned sequences in each of the groups defined by the combination of conservation and belt-contacting criteria, and experimentally assessed 69 total proteases. Of these, 68 (99%) were active and 18 (26%) showed activity at least as high as that of wild-type BoNT/X protease (**Extended Data Fig. 3g**). We selected one belt-constrained variant X/D1 and one belt-unconstrained variant X/D2 for further characterization. Thermal denaturation measurements revealed that the melting temperatures of X/D1 and X/D2 were 22 °C and 14.5 °C higher than that of wild-type BoNT/X protease, respectively (**Extended Data Fig. 3h**). The more stable X/D1 variant reached higher luciferase activation in the PACE host, but catalytic efficiencies ($k_{cat}/K_m$)compared to wild-type BoNT/X protease were reduced by 2-fold for X/D1 and 1.4-fold for X/D2 (**Extended Data Fig. 3, i and j**).

Next, we tested whether redesigned BoNT proteases can retain their compatibility with BoNT heavy chain-mediated intracellular delivery. We used a luciferase assay in which cytosolic delivery of the luciferase fragment HiBiT complements a cytosol-expressed LgBiT fragment, reconstituting the full luciferase enzyme and yielding luminescence[57,58]. We purified BoNT toxins consisting of catalytically inactive versions of redesigned or wild-type protease fused with the HiBiT luciferase reporter and complexed with the translocation domain of BoNT/A. Treatment of reporter N2A cells with toxin containing redesigned BoNT/X protease generated luminescence signal comparable to the signal from toxins containing wild-type BoNT/X protease and above that observed from treatment of HiBiT peptide alone. This result confirms that redesigned BoNT/X protease is compatible with BoNT-mediated delivery into mammalian cells, and the belt-constraining criterion was not necessary to retain this function (**Extended Data Fig. 3k**).

**Supplementary Note 2. Prediction of evolvability from redesign metrics.**

Across the panels of both ProteinMPNN and PROSS redesigns, we found that soluble expression does not correlate with ProteinMPNN negative log-likelihood redesign sequence scores for the apo BoNT/E structure (**Extended Data Fig. 7a-b**). However, for the four evolved starting points ProteinMPNN negative log-likelihood correlates with protein melting temperature, suggesting that redesign score can be used to predict stable starting points (**Extended Data Fig. 7c**). Examining the function of genotypes evolved, correlating circuit activation on evolution substrates did not correlate with apo structure scores for mutations evolved to cleave those substrates (**Extended Data Fig. 7d-g, top**), indicating that the explicit design objective for sequences that fold to the BoNT/E structure does not explain evolved activity. Separately, activity for evolved mutations modestly correlate with scores for those mutations in the respective protease•substrate complex in the D3 and PROSS1 backgrounds, although not in the BoNT/E or D4 backgrounds (**Extended Data Fig. 7d-g, lower**). A high ProteinMPNN score (low probability) appears to be predictive of low activity.

## Supplementary Note 3. Identification of an ataxin-2 target peptide.

We predicted a potential ataxin-2 binding register within the BoNT/E binding groove by placing Ile1193 at the P3 position of the protease, since our substrate profile data indicated a strong preference for Ile at this position (**Extended Data Fig. 4a**). This binding mode places the putative scissile bond of ataxin-2 between His1195 and Ala1196 (**Fig. 6a**). To validate that this region is accessible to protease-catalyzed cleavage, we designed a mock ataxin-2 substrate with the TEV protease substrate inserted within this target sequence. Co-transfection of plasmids expressing the mock substrate and TEV protease led to complete degradation of the substrate, supporting that ataxin-2 can be efficiently cleaved by a protease that targets a peptide within this region (**Extended Data Fig. 8a**).

## Supplementary Note 4. Evolutionary trajectory for the ataxin-2 cleavage campaign.

Proteases were first evolved to cleave a stepping-stone that introduces the single ataxin-2 residue that reduces starting point activity the most: assaying protease cleavage circuit activation on each single-residue substitution of ataxin-2 to the amino acid in SNAP-25 at the corresponding position of anticipated substrate binding identified the P2 D>Y mutation as the most challenging (stepping-stone 1, SS1; **Extended Data Fig. 8b-c**). Phage pools evolved on this substrate were next evolved on the complete ataxin-2 target peptide sequence with increasing levels of stringency (**Extended Data Fig. 8d, Supplementary Fig. 6**). Purification and cleavage assays of variants emerging from high-stringency ePACE confirmed that evolved proteases could cleave the ataxin-2 target peptide flanked by G/S-rich linkers encoded in the standard PACE selection circuit. However, these proteases were inactive on substrates without the flanking linkers, demonstrating that they evolved recognition of linker sequences that are not present in the native ataxin-2 protein (**Supplementary Fig. 7, j and k**). We therefore designed new PACE substrates in which we replaced the G/S linkers with an equal length of ataxin-2 residues from either side of the target. PACE on this substrate proceeded with increasing stringency followed by dual selection for ataxin-2 cleavage and against SNAP25 cleavage (**Extended Data Fig. 8d**).

**Supplementary Note 5. Observations of ataxin-2-evolved protease genotypes.**

Luciferase activity assay of consensus clones derived from each starting point demonstrated ataxin-2 cleavage activity and minimal off-target SNAP25 cleavage activity, demonstrating that dual selection PACE generated proteases highly specific for the desired target over the native substrate (**Extended Data Fig. 8f**). Stronger ataxin-2 cleavage was observed for clones evolved from D3 (D3(428) series), the most stable starting point, than for those evolved from wild-type BoNT/E (E(428) series). Although D2-evolved proteases did not yield higher activity than the wild-type-evolved proteases, they had higher thermal stability (**Supplementary Fig. 8**). We next confirmed that evolved proteases cleave ataxin-2 at the targeted scissile bond via mass spectrometry, although we note that the cleavage site was not accurately predicted by AlphaFold3[53] (**Extended Data Fig. 9a-d and Supplementary Note 6**). We therefore focused subsequent structural analysis on the apo protease (**Extended Data Fig. 8e and Supplementary Note 6**).

For the top-performing redesign-evolved variant D3(428)2, evolved residues were distributed along the substrate binding groove, with clusters of mutations proximal and distal to the cleavage site (**Extended Data Fig. 8e, left**). Mutations to the stretch of residues 162-166 increased hydrophobicity, suggesting that burial of this surface with the nearby P3-P9 substrate residues drives binding to ataxin-2. Notably, only the S162I mutation in this pocket also occurs in wild-type-evolved clones; the other four are unique to design-evolved clones. The K225 residue, previously described to form a salt bridge with P2 D residue in SNAP-25[54], mutated to I in this evolved protease which may support binding with the new P2 Y residue in ataxin-2. Based on the occurrence of N164I and I165T in both of the highest activity D3-evolved clones following dual selection but not in any earlier positive-only selection populations, (**Fig. 6b and Supplementary Table 12**), specificity for ataxin-2 over SNAP-25 is likely conferred by either or both of these mutations.

The wild-type-evolved clones had fewer total mutations, fewer mutations unique to the starting point, and fewer evolved contacts distal to the cleavage site compared to the redesign-evolved clones. Evolved residues were found along the expected P5-P4’-interacting region of the substrate binding groove (**Extended Data Fig. 8e, right**). In the top wild-type-evolved protease E(428)4, P5-P3 in the substrate are accommodated by N161K, S162C, and G220I, which are alternatives to other mutations at the same positions in the design-initiated evolutions. Residues known to bind C-terminal substrate residues in the native protease evolved into a new P2’-P4’ binding pocket formed by G353S and Y355C[55], along with the additional S66Y mutation that arose during dual selection.

**Supplementary Note 6. Alphafold3 prediction and mass spectrometry determination of ataxin-2 protease cleavage sites.**

To gain insight into how the alternative mutations found by design and wild-type starting proteases impart new specificity, we predicted the structures of evolved protease•ataxin-2 complexes using AlphaFold3. To evaluate whether AlphaFold3 accurately models complexes between evolved proteases D3(428)2 or E(428)4 and the ataxin-2 substrate, we used AlphaFold3 to predict the substrate register of ataxin-2 in the protease binding groove. The predicted scissile bond in the ataxin-2 substrate was defined to be the substrate peptide bond in the Alphafold3 complex structure with closest proximity to the catalytic $Zn^{2+}$ ion and catalytic residue Y351 that stabilizes the tetrahedral intermediate. The predicted substrate register in the binding groove of D3(428)2 and E(428)4 shifted the scissile bond from the original alignment, with P1200-T1201 in the scissile bond position for D3(428)2, and P1203-S1204 for E(428)4. However, experimental determination of the cleavage sites by mass spectrometry revealed product masses consistent with the originally anticipated cleavage site H1195-A1196 for both proteases, demonstrating that AlphaFold3 did not accurately predict the substrate binding register (**Extended Data Fig. 9a-d**).

**Supplementary Note 7. Molecular dynamics simulations of protease•SNAP-25 complexes.**

We sought to explain how the PACE-reprogrammed proteases diminish undesired SNAP-25 cleavage by investigating the molecular dynamics of the protease•SNAP-25 complex. Performing molecular dynamics simulations initiated with each AF2-predicted complex, we observed that each evolved protease sampled fewer hydrogen bonds and salt bridges to the SNAP-25 substrate than the corresponding parent SNAP-25-cleaving protease over the 3-μs time course. Q-native scores for evolved protease complexes were also lower than for the parent proteases, suggesting that the evolved proteases spend less time in a SNAP-25-cleaving conformation (**Extended Data Fig. 10d-g**). These simulation results support that specificity evolution yielded proteases that interact less favorably with the negative selection substrate, accounting for their high specificity observed in biochemical assays. These metrics, however, did not differ between wild-type-evolved and design-evolved proteases, and thus they cannot explain why only the design-evolved proteases virtually eliminate negative selection substrate cleavage.

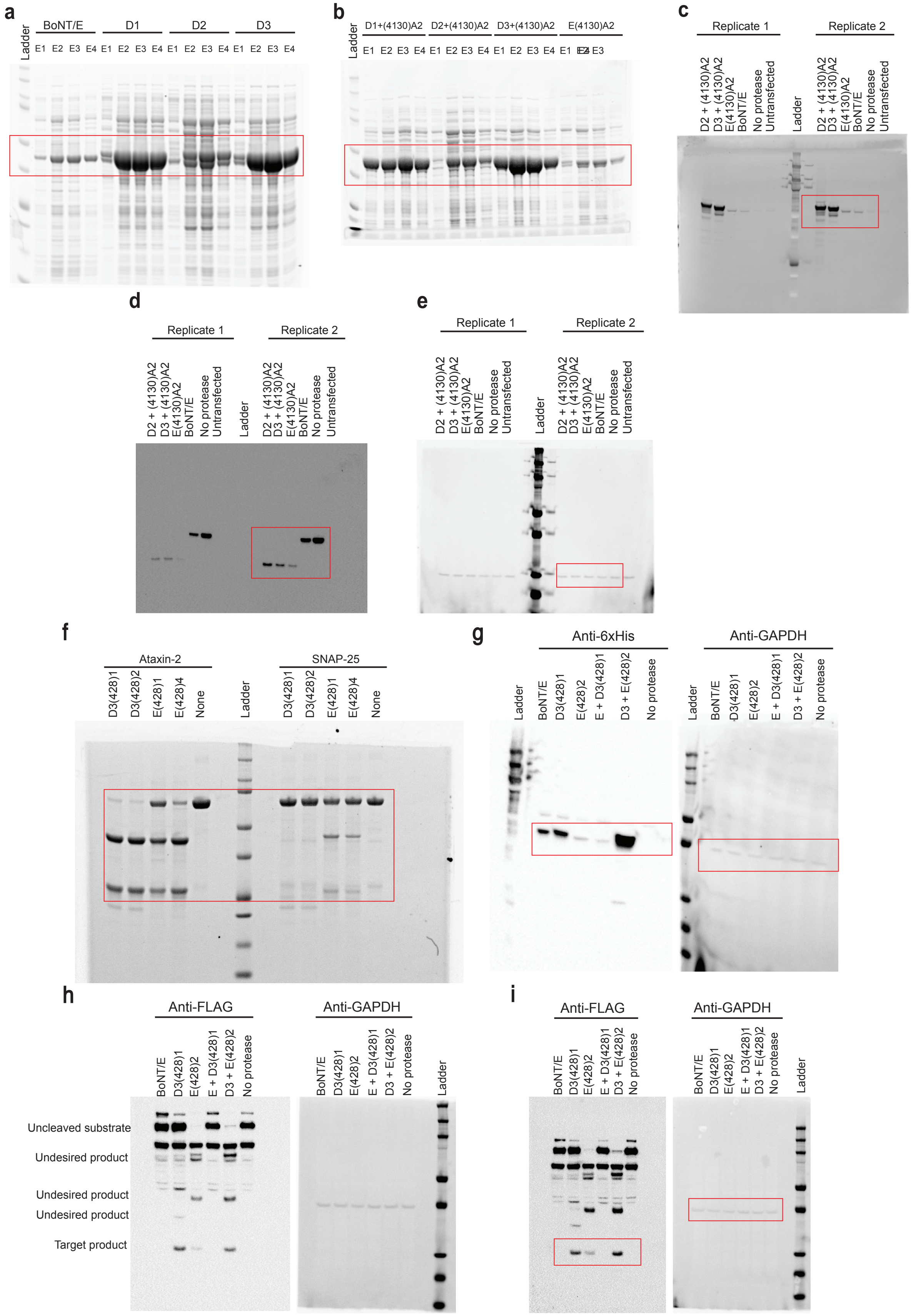


**Supplementary Figure 1. Uncropped PAGE gels and western blots.** (**a**) BoNT/E redesign soluble yield assay PAGE gel in **Fig. 1d**. (**b**) PTEN-cleaving protease soluble yield assay

PAGE gel in **Fig. 2b**. (**c** to **e**) Western blots for PTEN cleavage assay in **Fig. 2f**. Anti-6xHis blot showing 488 nm fluorescence for protease expression (**c**)**.** Anti-FLAG blot chemiluminescence image for FLAG-tagged PTEN (**d**)**.** Anti-cofilin 680 nm fluorescence image for the **(d) blot** (**e**). (**f**) Gel shift assay for selected specificity PAGE gel in **Fig 6g**. (**g** to **i**) Western blots for the ataxin-2-cleaving protease mammalian expression assay in **Fig. 6h**. Protease expression by detection of 6xHis tag (**g**). Replicate 1 ataxin-2 full-length and cleavage products seen by detection of FLAG tag (**h**). Replicate 2 ataxin-2 full-length and cleavage products seen by detection of FLAG tag (**i**). Deduced ataxin-2 uncleaved substrate and cleavage products labelled.

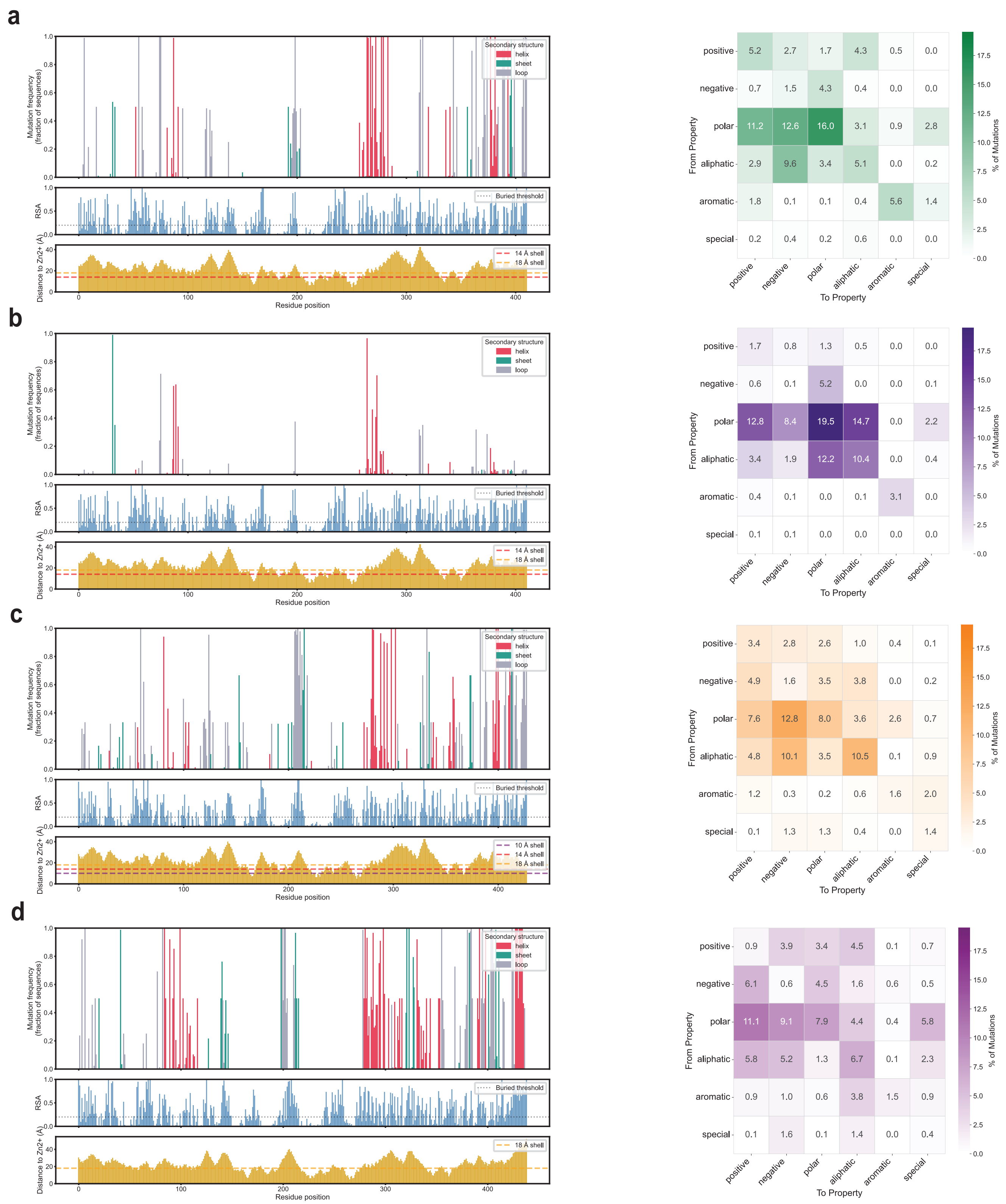


**Supplementary Figure 2. Properties of redesigned protease mutations for each BoNT serotype and redesign method.** (**a** to **d**) Analysis of redesign residues from ProteinMPNN of BoNT/E, BoNT/F, and BoNT/X, and PROSS of BoNT/E, respectively. Mapping of mutations to secondary structure element and solvent accessibility (left). Transition matrices indicating the

percentage of mutations with each property change. Structural features of redesign mutations are tabulated in **Supplementary Table 4**. RSA, relative solvent accessibility.

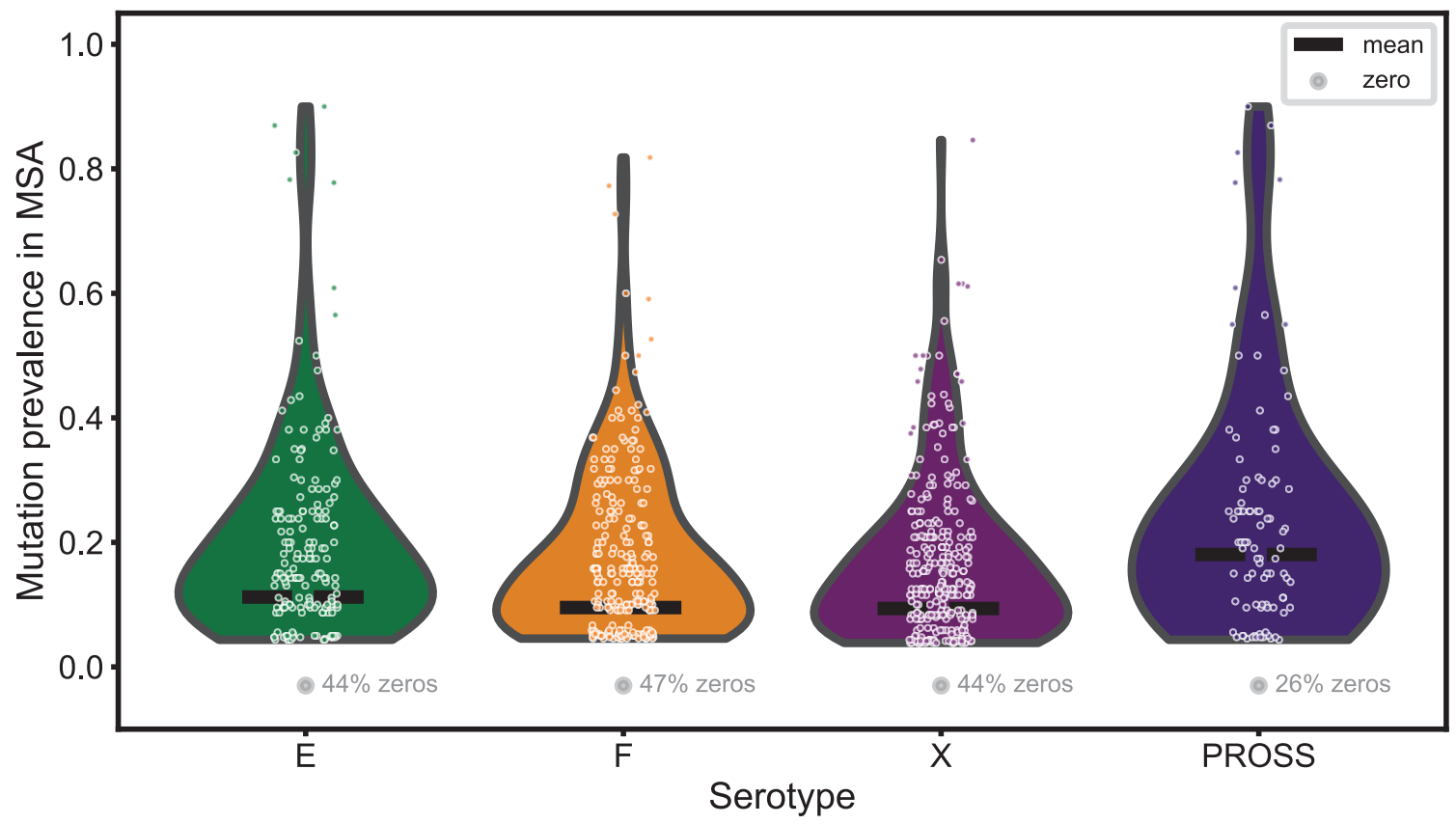


**Supplementary Figure 3. Frequency of redesign mutations in multiple sequence alignments (MSA)s by serotype and redesign method.** Prevalence in MSA is defined as the fraction of sequences in the MSA used for redesign conservation analysis with the same residue as the redesign mutation at the same position. E, F, and X indicate ProteinMPNN redesign with BoNT/E, BoNT/F, and BoNT/X, respectively, and PROSS refers to redesign of BoNT/E with PROSS.

a

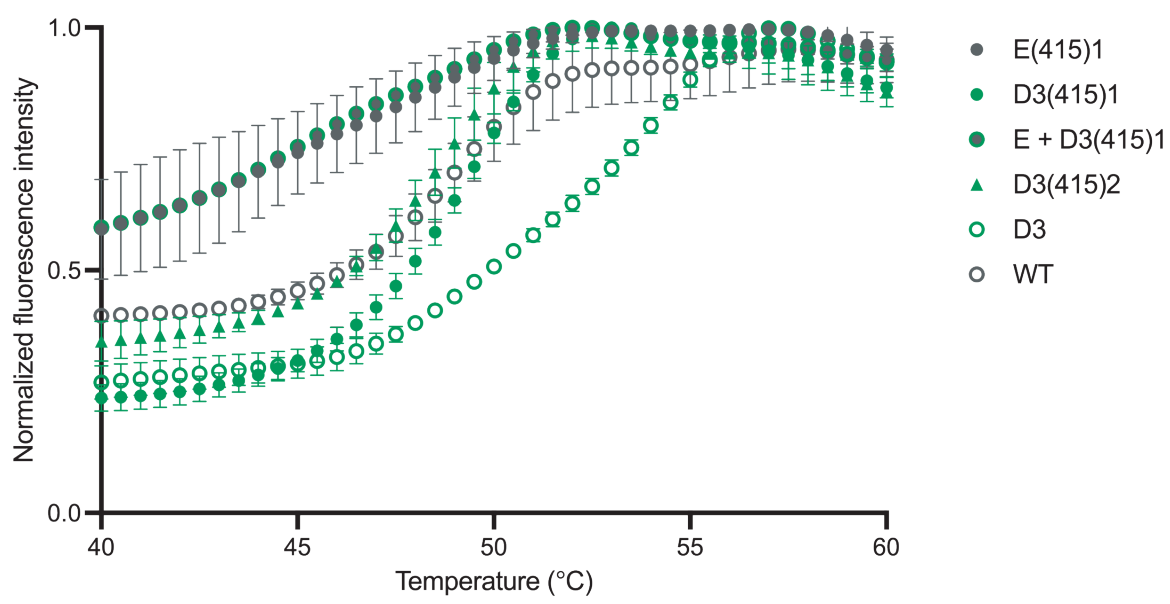


| | E(415)1 | D3(415)1 | E + D3(415)1 | D3(415)2 |
|---|---|---|---|---|
| Tm (°C) | 46.2 | 49.5 | 45 | 48.7 |

| | WT | D3 |
|---|---|---|
| Tm (°C) | 49 | 54.5 |

b

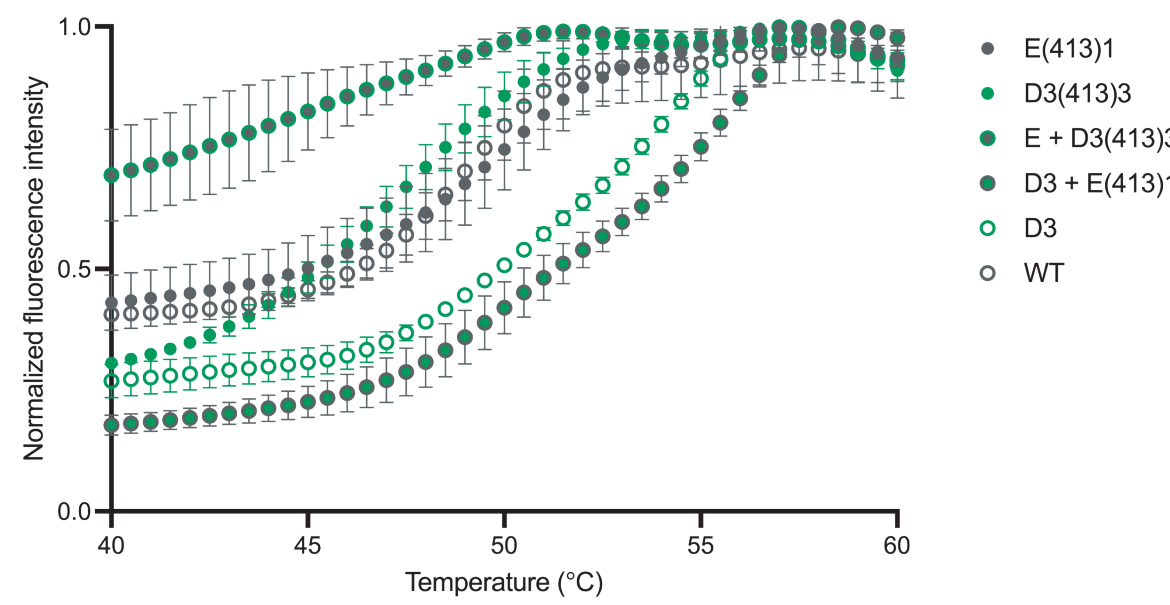


| | E(413)1 | D3(413)3 | E + D3(413)3 | D3 + E(413)1 |
|---|---|---|---|---|
| Tm (°C) | 49.8 | 47.5 | 45.7 | 55.7 |

| | WT | D3 |
|---|---|---|
| Tm (°C) | 49 | 54.5 |

c

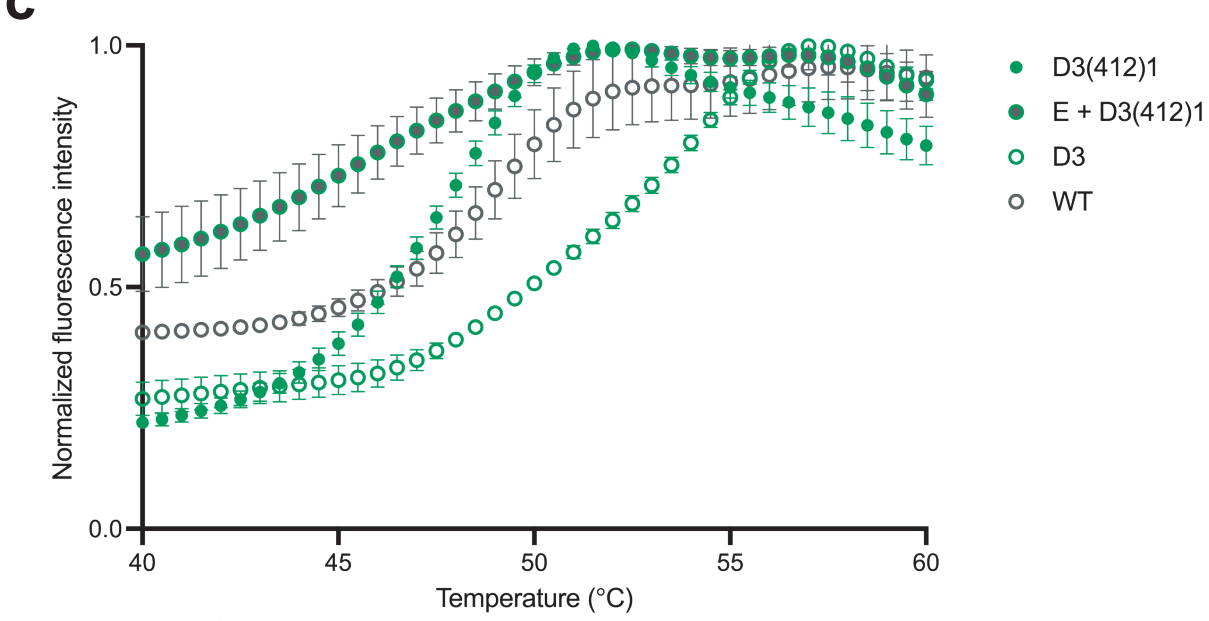


| | D3(412)1 | E + D3(412)1 |
|---|---|---|
| Tm (°C) | 48 | 44.7 |

| | WT | D3 |
|---|---|---|
| Tm (°C) | 49 | 54.5 |

**Supplementary Figure 4. Thermal stability of selected proteases from the comparison evolution panel evolved from wild-type or redesigned starting points and their grafts.** Tm values were calculated as the temperature at which slope of the SYPRO Orange fluorescence intensity curve is maximal and are reported in Fig. 3d. (**a**) Proteases from lowest difficulty evolution on substrate 415. (**b**) Proteases from intermediate difficulty evolution on substrate 413. (**c**) Proteases from hardest difficulty evolution on substrate 412.

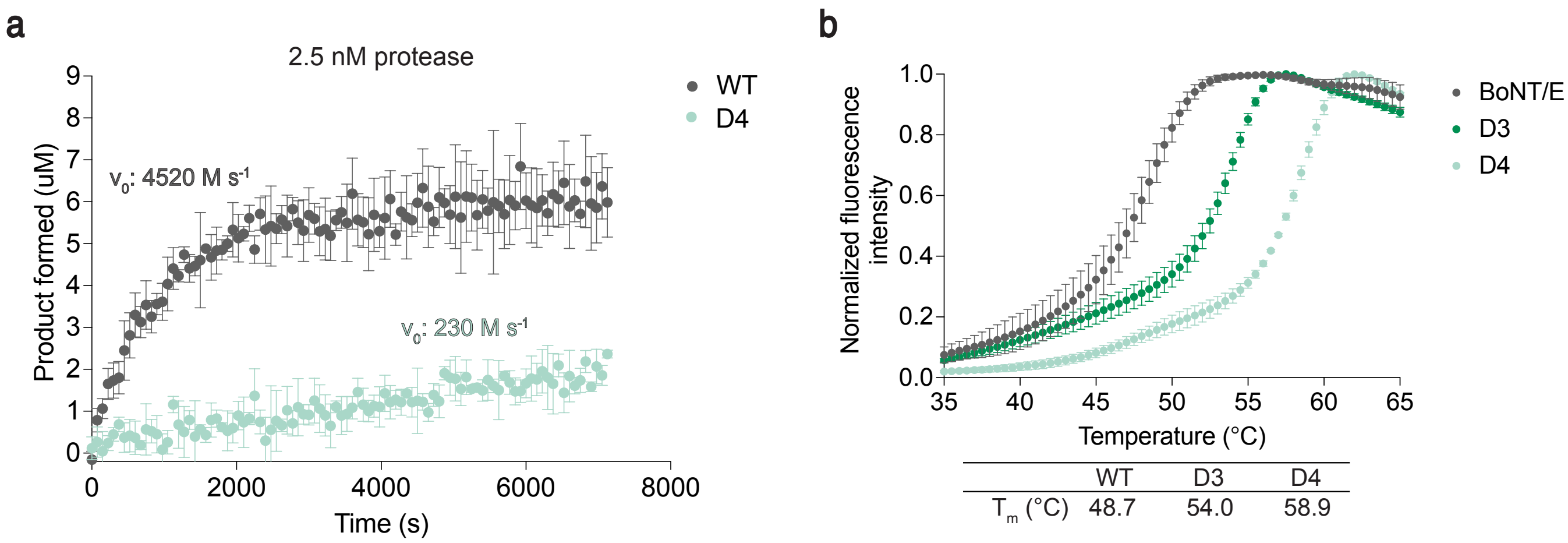


| | WT | D3 | D4 |
|---|---|---|---|
| $T_m$ (°C) | 48.7 | 54.0 | 58.9 |

**Supplementary Figure 5. Catalytic activity and stability of the alternative ProteinMPNN redesign D4.** (**a**) In vitro cleavage time course of the SNAP-25 FRET substrate at 5 µM with 2.5 nM of the indicated protease. (**b**) Protein melting curves using SYPRO Orange fluorescence to measure thermal stability and the resulting melting temperatures. Values and error bars represent mean±s.d. of three replicates.

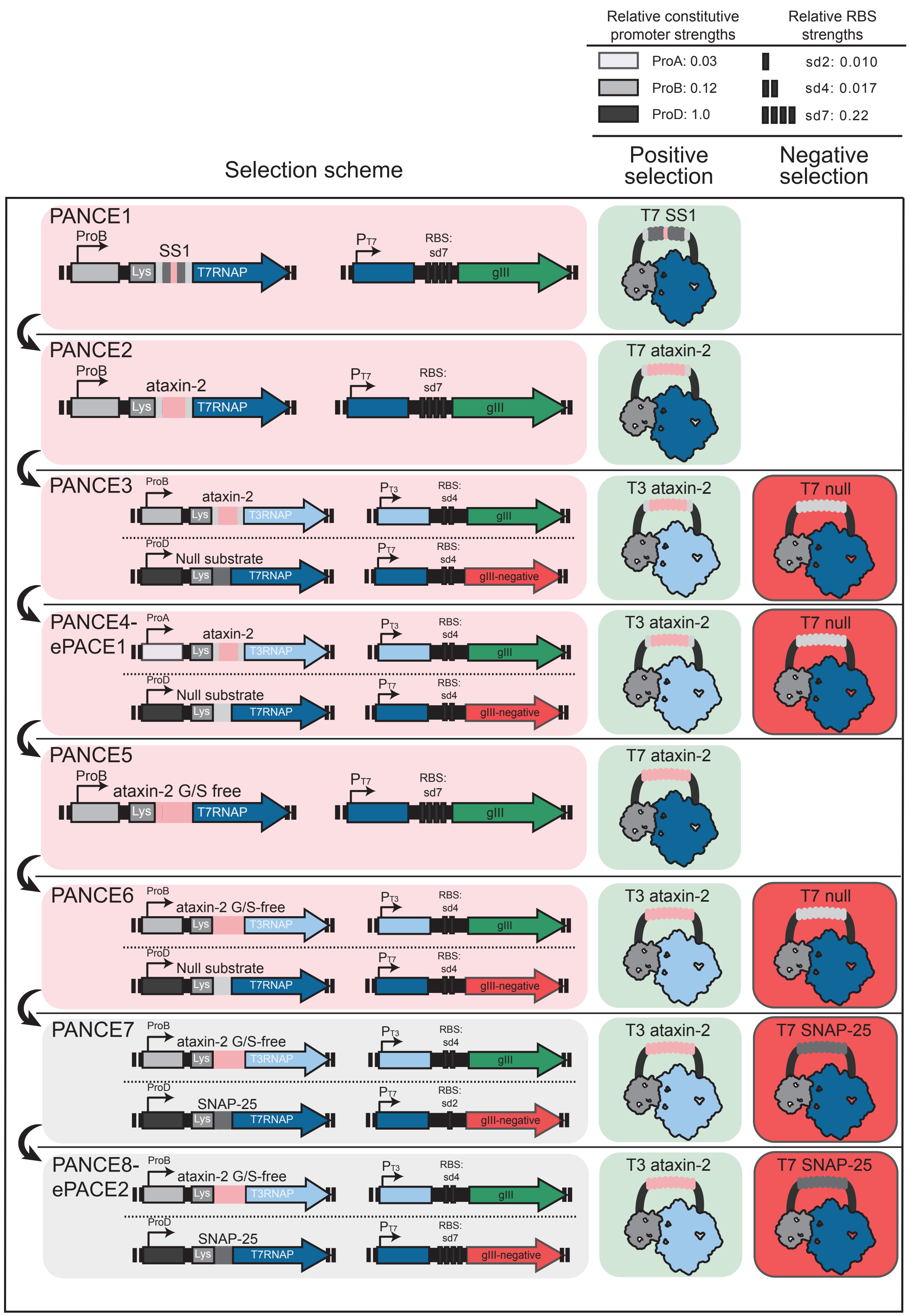


**Supplementary Figure 6.** PANCE and PACE hosts by positive and negative substrates used over the evolutionary trajectory to evolve ataxin-2-specific proteases overviewed in **Extended Data Fig. 8d**. SS1, stepping-stone 1.

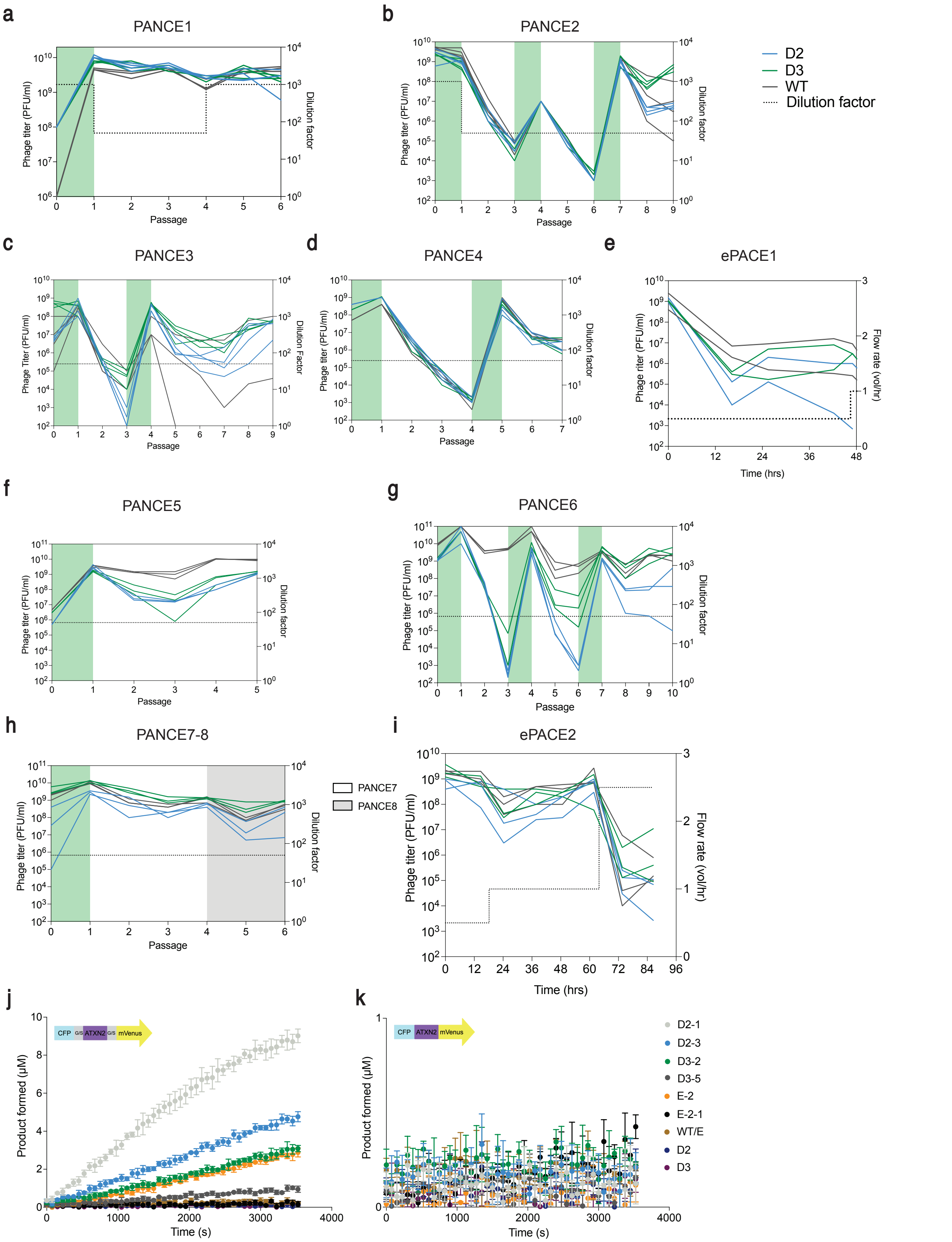


**Supplementary Figure 7. Phage titers over each stage of the ataxin-2 protease evolution overviewed in Extended Data Fig. 8d and intermediate characterization.** (a-i)

Phage titer by PANCE passage or PACE time point. Host substrate plasmids provided in Supplementary Fig. 6. Green indicates drift passages. (**j**) Time course for ePACE1-evolved variants and unevolved starting points cleaving the G/S linker-containing substrate from ePACE4. (**k**) Time course for ePACE1-evolved variants cleaving the substrate without the G/S linkers. Assays were performed with 1 μM protease and 10 μM substrate. Data in j-k are shown as mean±s.d. for three replicates.

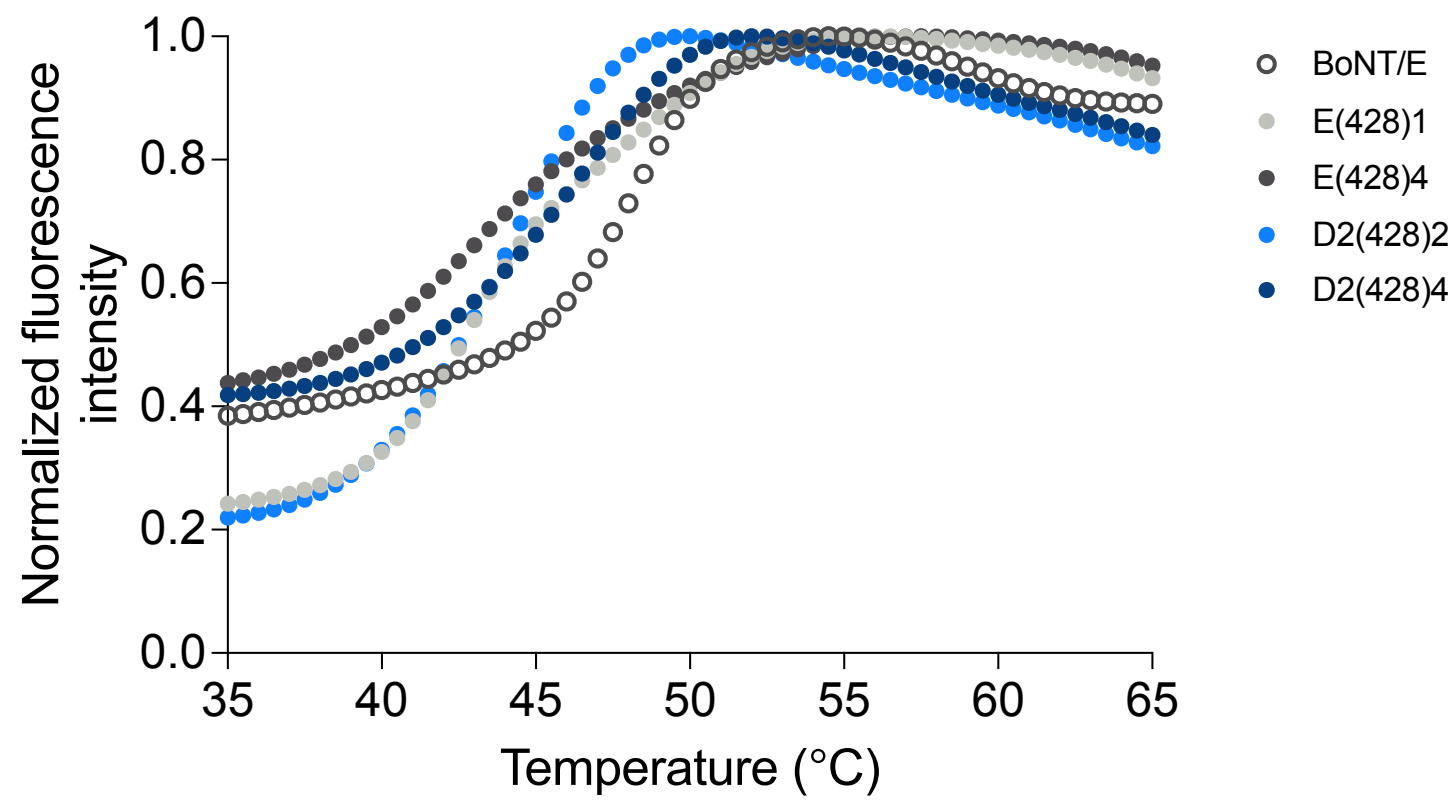


| | BoNT/E | E(428)1 | E(428)4 | D2(428)2 | D2(428)4 |
|---|---|---|---|---|---|
| $T_m$ (°C) | 48.0 | 42.2 | 42.8 | 44.2 | 46.5 |

**Supplementary Figure 8. Thermal stability of D2-evolved ataxin-2-cleaving proteases in comparison to wild-type-evolved proteases.** Tm values (bottom) were calculated as the temperature at which slope of the SYPRO Orange fluorescence curve (top) is maximal.
