# Supplementary material for: AI-redesigned starting points and outcomes enhance protein evolution
Source: Nature. 2026 Jul 22;657(8131):525–38. doi: 10.1038/s41586-026-10820-0 (PMC13477572; doi:10.1038/s41586-026-10820-0)
Supplement: Supplementary file 3 [file 41586_2026_10820_MOESM4_ESM.pdf]

nature portfolio

Peer Review File

AI-redesigned starting points and outcomes enhance protein evolution

Corresponding Author: Professor David Liu

Any redactions in this file are there to maintain patient confidentiality, the confidentiality of unpublished data, or to remove third-party material.

This file contains all reviewer reports in order by version, followed by all author rebuttals in order by version.

Version 0:

Reviewer comments:

Referee #1

(Remarks to the Author)
In this manuscript, Krasnow et al. present an experimental workflow which integrates computational sequence redesign (ProteinMPNN and benchmarked against PROSS) with continuous evolution (PACE) to achieve functional outcomes that are difficult to access when starting from the natural enzyme. The authors support the premise that natural proteases are constrained by their low stability using thermal stability measurements (melting temperature) and soluble expression yield. The central conceptual claim, that increasing the stability of enzymes via computational redesign enables otherwise-inaccessible genotypes, is well substantiated by parallel evolution, deep sequencing of endpoint genotypes, and systematic grafting of mutations across the wild-type and redesigned backgrounds. Additionally, the therapeutic target ataxin-2 provides a potential application of the protease function and specificity engineering.

Overall, the authors did an excellent job defending their claim. However, I do not find the findings to be surprising. As the authors have acknowledged in Refs 25 and 36, the concept that the 'evolvability' of a protein often correlates with its stability has been established by experimental means over several decades now. The key innovation of this paper is to show that computational redesigned sequences can indeed be used as starting points in PACE, and by doing so, the additional stability enabled access to the destabilizing (yet functionally enhancing) mutations that could not be accommodated by natural proteins, corroborating the abovementioned concept. However, computational redesign to improve protein stability is by no means the only way to yield more stable starting points. Have the authors attempted to use their own solubility-improving PACE circuits to improve the expression of the natural proteases? What about other directed evolution techniques? In other words, the main idea – that stability enhances evolvability – is well known. And, it is also well known that computation can be used to enhance stability. This paper simply takes two well-known concepts and mixes them together. From a utility perspective, this is very exciting and potentially useful. From a conceptual or innovation perspective though, I find this paper does not warrant publication in a journal targeting a very broad audience.

My other major concern is that the experiments were all aimed at confirming the authors' hypotheses, without null testing. For example, one idea, is the authors could include a lower expressing, similar activity starting point or similar expressing, higher activity starting point and compare the evolution outcomes. I suspect these evolutions could yield better performing variants than the WT starting point, which would contradict the authors' claim that higher stability always give better evolution outcomes. This would actually be exciting, as it would show some additional nuance to the literature, which as of now, already directly correlates stability and evolvability. Similarly, the authors could test a series of similarly stabilized redesigned variants, and possibly test if evolvability does not only correlate with stability (perhaps some variant with a lower Tm wins?). Again, this would at least be new knowledge, if it turned out to be true.

Minor issues

1. If the low expression of natural starting point is the limiting factor for accumulating destabilizing mutations, have the authors tried simply increasing the strength of the promoter and RBS or protein solubility tags to boost the expression?

2. In PACE, there is also selection pressure for higher solubility/better expression. Can the authors decouple the effect of

some mutations boosting expression vs. improving catalytic function?

3. The authors used ProteinMPNN and benchmarked with PROSS. It would be more convincing if some variants from PROSS are also subjected to PACE and draw similar conclusions.

4. The authors claimed 'specificity' with only comparison to the native substrate SNAP-25. I don't think it's sufficient to claim specificity.

5. Fig. 1d shows D1-D3 all have higher expression level than WT. Yet, in the PACE evolution campaign for Ataxin-2, D2 evolved variants showed even lower activity than WT-evolved variants for on-target substrate and higher off-target activities (Fig. 4e). How do the authors account for this observation?

6. In Ext. Data Fig. 6 selection scheme for PANCE1, what does SS1 stand for?

7. Page 14, "We predicted a potential ataxin-2 binding register within the BoNT/E binding groove by placing Ile1193 at the P3 position of the protease, since our substrate profile data indicated a strong preference for Ile at this position (Fig. 3a).": The reference to Fig. 3a does not support the claim.

(Remarks on code availability)

Referee #2

(Remarks to the Author)
Summary: Krasnow et al. couple deep learning-based sequence redesign with laboratory directed evolution to show that enhancing protein stability of evolutionary starting points expands the accessible sequence space during evolution. Using ProteinMPNN, the authors generated stabilized botulinum neurotoxin (BoNT) proteases and subsequently performed parallel phage-assisted continuous evolution campaigns with the wild-type proteases, selecting for proteolytic activity and/or specificity in two contexts: 1) on similar, yet non-native substrates or 2) on a disease relevant ataxin-2 protein. In all campaigns, sequence-redesigned starting points yielded evolved variants with enhanced activity and specificity (vs. variants derived from the wild-type starting point). Critically, the amino acid substitutions uncovered in the re-designed protease campaigns were largely incompatible with the wild-type background due to protein stability penalties, underscoring the impact of sequence redesign toward evolving otherwise inaccessible variants.

Impact: This study offers a practical strategy for addressing a commonly observed limitation of laboratory evolved proteins: reduced activity and/or specificity for a non-native function compared to the wild-type protein on its native function. While the principle of protein stability conferring mutational robustness (and therefore enhanced evolvability) is well established, generating stable proteins as inputs for evolution experiments has been historically cumbersome (e.g., requiring protein stability selections, modifying the chaperone network, etc.). Sequence redesign with ProteinMPNN appears to be a promising approach to rapidly generate starting points for laboratory evolution, given the observation that proteases evolved from redesigned starting points consistently displayed enhanced activity, solubility, and specificity vs. those evolved from wild-type. Though this study implements existing tools/methods and focuses on a single protein class, the head-to-head comparisons afforded by the parallel campaigns are valuable case studies for the protein engineering community. Furthermore, the evolved redesigned (D3) ataxin-2 proteases themselves are useful biotechnological tools and serve as a proof-of-concept for evolving highly active and specific proteases for cleavage of therapeutically relevant targets. Future work to establish generalizability of the overall strategy to alternative protein targets is greatly anticipated. The work is technically well-done and suitable for publication.

Major Concerns: n/a

Minor Comments:

1) Supplementary note 1:
• First paragraph references Fig. S2b, but is describing data displayed in Ext. Data Fig. 3b.
• Second paragraph: References to Fig. S3a-e should be re-labeled to Ext. Data Fig. 3f-k. Additionally, Tm and kcat/KM data for X/D1 and X/D2 appear to be flipped. For example, X/D2 variant is described as more stable, yet has a Tm of 60 vs 68 for X/D1.

2) pp. 9-10, "E(4130)A2 is less stable than wild-type BoNT/E protease (Fig. 2c). We observed that the mutation-grafted D1, D2, and D3 enzymes…express to a higher level in E. coli cells than either BoNT/E or E(4130)A2 (Fig. 2b).": Expression and Tm data for WT BoNT/E are not shown in Fig. 2b and 2c, respectively. These should be added to support the claims in the two sentences listed.

3) p. 14: Substrate profile data is listed as Fig. 3a, yet is actually shown in Ext. Data Fig. 4.

4) Ext. Data Fig. 8a: Blue residues not colored as in Fig. 4.

5) Suppl. note 3: Fig. S13 doesn't exist. Rename to Ext. Data Fig. 8a?

6) p. 16: First reference to Fig. 5b in the text should be changed to Fig. 5a.

7) p. 17: In paragraph 2, when describing cleavage rate assay, change units of mM to uM.

8) Fig. S3b: D3(428)1 also appears to generate some undesired product (second band from bottom in lane 2), though curiously this is rescued when grafting into E(428)2 (fifth lane). It would be useful to comment on what whether that is a bona fide undesired cleavage byproduct.

(Remarks on code availability)

Referee #3

(Remarks to the Author)
I co-reviewed this manuscript with one of the reviewers who provided the listed reports.

(Remarks on code availability)

Referee #4

(Remarks to the Author)
Krasnow et al. compare the evolvability of redesigned proteins (using models like ProteinMPNN) with their wild-type counterparts, using BoNT proteases as case studies. The authors show that sequence-redesigned enzyme have greater evolvability, in part mediated by enhanced stability of the starting point enzyme. The authors conduct substantial experimental work including continuous evolution experiments that help to concretely establish claims around evolvability, as well as thorough biochemical and functional assays to characterize the evolved variants.

This paper excels through the strength of its experimental data, especially in the ability to sample many evolutionary trajectories in laboratory settings, as well as a breadth of orthogonal readouts supporting the conclusions (including soluble expression, Tm, activity and kinetics, luciferase circuit fitness, sequencing, mammalian cell assays, gel shift, MS, and MD). However, the paper has much more limited conceptual novelty; it is well established dogma in the directed evolution field that stability promotes evolvability (Bloom et al., PNAS, 2006), which this paper does not overturn, and the methods the authors use (in particular, ProteinMPNN) have already been shown to improve directed evolution in lower throughput settings (e.g., Sumida et al., 2024).

Our view of this paper is therefore as a borderline case and for which we would request responses to our questions below, especially regarding the conceptual novelty of this work, before we can make a decision on whether to recommend publication of the paper in Nature.

Major comments:

• ProteinMPNN is already well known to rescue or improve protein expression, stability, and functon(e.g., Sumida et al., 2024, as cited by the authors). In addition, as the authors note, prior work has shown that ProteinMPNN can increase the thermal stability of wild-type enzymes through a systematic pipeline, and that sequence-redesigned enzymes can be evolved to improved activity for a non-native remote C(sp3)-H hydroxylation reaction (King et al., 2025). Given this context, it is critical for the manuscript to more clearly articulate the unique value and new insights enabled specifically by the present study.

• It would be especially valuable to summarize grafting asymmetry quantitatively and experimentally across campaigns. This could be done by comparing the fraction of wild type-evolved mutation sets that remain functional in D3, versus the fraction of D3 evolved mutation sets that remain functional in wild type, ideally alongside activity and stability effect sizes. This appears likely to be achievable from data already generated and would sharpen the robustness claim considerably, though the authors should regenerate new data as appropriate to answer this question.

• An analysis that could add substantial value to the community is a comparison of ProteinMPNN sequence scores or likelihoods for PACE-evolved variants, versus the initial seed(s). This would help clarify the division of labor between model-guided redesign and evolutionary search, and could show whether redesign alone would be likely to generate variants in the same fitness region (which is unlikely). Relatedly, Ext. Data Fig. 1 already provides a useful in silico evaluation of redesigned seeds (AF2 confidence, RMSD, and sequence divergence), but the manuscript is also in a strong position to provide a post hoc benchmark of redesign metrics against empirical outcomes. Correlation plots between AF2-derived metrics (for example pLDDT, iPAE, RMSD, or designability type metrics) and experimental outcomes such as soluble expression, Tm, activity retention, or later evolvability would be very informative, even if none show any signal. In particular, it would be valuable to test whether any computational metric has a signal for predicting good starting points with D3 like behavior.

• Relatedly, a residue-level analysis of where MPNN-introduced mutations cluster (ex: surface versus core, secondary structure context, distance to active site, or packing networks) would also strengthen the mechanistic interpretation, even if descriptive only. The distance and conservation cutoff choices seem reasonable and the empirical comparisons are useful,

but the cutoffs still read somewhat heuristic. A brief, more explicit justification of these cutoffs would help, especially around the tradeoff between preserving catalytic and substrate-recognition determinants and allowing enough redesign freedom to improve stability and robustness. A small table summarizing the number of frozen versus redesignable residues for each ProteinMPNN setting and serotype would also make the design strategy easier to evaluate and reproduce.

• Finally, much of the current evidence focuses and the start and endpoints of evolution, with the grafting experiments carrying much of the mechanistic interpretation. The manuscript could be further strengthened by a more explicit trajectory level comparison of wild-type versus redesigned backgrounds across replicates. For example, presenting forms of "adaptation dynamics" such as i) time to stable propagation, ii) time to a predefined activity threshold, or iii) success frequency under matched stringency schedules. If intermediate timepoint samples are available, a simple diversity over time analysis (for example high abundance genotype counts, mutational distance from seed, or population entropy) would provide direct support for the accessible sequence space and evolvability claims.

Minor comments:

• Protein expression levels in Figs. 1d and 2b should be quantified.

• In Ext. Data Fig. 2e, the kinetic measurements appear to differ substantially from those in Fig. 1f (for WT, D1, D2, and D3). The authors should explain the source of these differences.

• Similarly, the scatter in Ext. Data Fig. 2b for D1–D3 appears different from the scatter plot in Fig. 1c. The authors should explain the discrepancy.

• Fig. 5g would benefit from quantification, including on-target/off-target fold changes across variants and direct comparisons of these fold changes. From the bar plots, the on/off ratio appears similar between D3(428)2 and E(428)4 evolved from the wild-type BoNT protease.

(Remarks on code availability)

Referee #5

(Remarks to the Author)
I co-reviewed this manuscript with one of the reviewers who provided the listed reports.

(Remarks on code availability)
The scripts seem basically correct to me, but there are several hard coded places which need to be modified if the authors expect other people to use this codebase.

Version 1:

Reviewer comments:

Referee #1

(Remarks to the Author)
In the revised work, Krasnows et al. have substantially addressed the concerns raised in the initial review through impressive additional experimental work. The characterization of evolutionary intermediates (Fig. 3a–c) along the evolution timescales demonstrates that redesigned starting points can evolve maximal activity faster than wild-type counterparts, and that wild-type starting points require additional (potentially stabilizing) mutations accumulated to access functionally-enhancing mutations; the LoopSeq analysis (Fig. 4) reveals that redesign-initiated pools converge on genotypes that wild-type pools do not sample, and do so with fewer mutations. The reversion analysis (Extended Data Fig. 5) is convincing, showing that wild-type-evolved proteases require compensatory stabilizing mutations while redesigned variants achieve comparable activity without this cost; and critically, the evolution of alternative redesigns D4 (stabilized but catalytically impaired) and PROSS1 (Fig. 5)—demonstrates that the benefits hold even when redesign involves trade-offs between stability and catalytic function. The experimental work was rigorous and substantially strengthens the manuscript's claims, and I commend the authors for the thoroughness of their response.

That said, I remain uncertain whether these advances represent sufficient conceptual novelty for Nature. While the data convincingly demonstrate that computational stabilization expands the fitness landscape and accelerates adaptation, the underlying mechanism continues to be an expected consequence of the well-established stability-evolvability synergy, that a more stable protein can better accommodate destabilizing mutations, permitting earlier access to locally beneficial genotypes. The practical value of this workflow is clear, but the conceptual insight that stability facilitates access to sequence space does not substantially deepen our understanding of protein evolution or the principles governing evolvability. Furthermore, although the authors claimed that this approach has been adopted in their own lab for additional protein evolution campaigns, it is not evident that this approach generalizes beyond the carefully designed BoNT protease system tested here. While the practical demonstration is undoubtedly valuable, this work remains fundamentally methodological rather than conceptual in nature. I defer to the editors of the fit.

1. Fig. 2d shows that D3+(4130)A2 has lower (but comparable) $k_{cat}/K_M = 0.0085\ \mu M^{-1}s^{-1}$ compared to E(4130)A2 ($0.010\ \mu M^{-1}s^{-1}$), yet Fig. 2e shows D3+(4130)A2 appears to be faster then E(4130)A2. Can the authors clarify this discrepancy?

2. Fig. 2f shows cleavage of FLAG-PTEN in mammalian cells. However, it is unclear whether equal amounts of FLAG-PTEN substrate were transfected across all conditions. The D2+(4130)A2 and D3+(4130)A2 lanes show robust cleavage product with no uncleaved substrate, yet no protease and BoNT/E condition showed abundant uncleaved product. E(4130)A2 shows weak product and also minimal uncleaved substrate. For E(4130)A2, very few cleavage product is shown with no uncleavage product. Otherwise, please clarify the band identities.

3. For Ext. Data Fig. 4a, how is starting activity defined? Does it represent phage enrichment?

4. For Fig. 3a-b, the flow rate for D3 is invisible. Are they the same as WT flow rate?

5. For Fig. 3a-c middle lower showing the initial reaction velocity, purification yield and lux fold change, I cannot find annotation for each of the isolated variants. Also, it might be helpful to label starting point in these graphs for comparison.

6. Does the N238K mutation identified in Fig. 3a truly destabilize the WT and WT-evolved variants?

7. A striking observation across Figs 3a–c and 5 is that grafting mutations evolved from WT variants onto redesigned backgrounds frequently yields superior activity compared to mutations evolved directly on redesigned starting points. For example, in Fig. 3a, D3+E(415)1 show higher circuit activation than some D3-evolved variants. This pattern appears general. Can the authors explain why these WT-evolved mutations were not accessed during redesign-evolution, despite being beneficial? Do they confer only marginal fitness advantages insufficient to afford selective advantage in PACE? Can the Hamming distance analysis (Fig. 4d) and fitness landscape visualization (Fig. 5d) account for why certain beneficial mutations remain unexplored in the redesign-evolved pools? This raises the mechanistic question of whether PACE selection stringency or sampling dynamics, rather than protein stability, limit access to certain high-fitness genotypes.

8. Related to point 7, if the grafting analysis reveals that WT-evolved mutations often further enhance redesign-evolved mutations, this has practical implications. Would the authors instead recommend a hybrid strategy: conducting parallel evolution on both WT and redesigned starting points, then systematically grafting mutations between backgrounds to identify combinatorial beneficial mutations? This could enable discovery of higher-activity variants. Please discuss whether this workflow would be practical and whether it would change the authors' recommendation for future protein evolution campaigns.

9. Fig. 6 demonstrates that redesign-evolved ataxin-2 proteases achieve dramatically higher selected specificity compared to the best WT-evolved variant. The authors show that D3-evolved proteases essentially eliminate detectable SNAP-25 cleavage while maintaining robust ataxin-2 activity. However, the mechanistic basis for this superior specificity remains unclear. Is this outcome serendipitous? Is it a consequence of the expanded fitness landscape allowing access to a particularly favorable specificity solution, or is there an underlying mechanism why redesign projects evolution toward higher specificity? The molecular dynamics simulations (Ext. Data Fig. 10d-g) show reduced H-bonds and salt bridges to SNAP-25 in evolved proteases, but this pattern does not distinguish between WT- and redesign-evolved variants. How do the authors explain the dramatic specificity difference given equivalent loss of SNAP-25 contacts?

Finally, I note that the manuscript's readability is quite low, and the figures are often confusing due to excessive layering of information. Many figures present multiple panels with complex genotype tables, activity readouts, and mutational annotations simultaneously, making it difficult to extract the main message. The authors should consider simplifying figure presentations, use clearer visual hierarchies, and potentially move some supplementary data to Extended Data to improve accessibility for readers.

(Remarks on code availability)

Referee #2

(Remarks to the Author)
The additional evolution campaigns and associated analyses (primarily Figs. 4 and 5, and Ext. Data Fig. 6) for the D4 and PROSS1 redesigned proteases included in this revision meaningfully improved the work. The new conceptual finding that redesign enhances adaptation rate in addition to expanding the functional sequence landscape (even for the D4 starting point where redesign compromised activity relative to wild-type) is an important observation that will help guide future experimental evolution campaigns. In this regard, the main impact of the work is a practical and potentially general strategy to bolster laboratory evolution studies. To facilitate adoption of this approach by the wider protein engineering community, identifying redesign metrics predictive of function as suggested by Reviewer 3 (beyond the weak correlations summarized in Ext. Data Fig. 7) will be important to triage experimental effort. However, as this is the first redesign+evolution study of this scale/throughput, the lack of clear predictive metrics does not detract from the work and highlights the value of integrating computational tools with parallelizable continuous evolution methods.

Krasnow et al. have sufficiently addressed all of our minor comments and the work is suitable for publication.

Minor typo:

p. 16, last sentence of paragraph 2: In-text reference to “Fig. 4a, middle, bottom” seems to actually refer to Fig. 4b

(Remarks on code availability)

Referee #3

(Remarks to the Author)
I co-reviewed this manuscript with one of the reviewers who provided the listed reports.

(Remarks on code availability)

Referee #4

(Remarks to the Author)
I thank the authors for their very thorough revision, which has yielded interesting new data, and so I am happy to recommend acceptance of this manuscript.

(Remarks on code availability)

Referee #5

(Remarks to the Author)
I co-reviewed this manuscript with one of the reviewers who provided the listed reports.

(Remarks on code availability)

Open Access This Peer Review File is licensed under a Creative Commons Attribution 4.0 International License, which permits use, sharing, adaptation, distribution and reproduction in any medium or format, as long as you give appropriate credit to the original author(s) and the source, provide a link to the Creative Commons license, and indicate if changes were made.
In cases where reviewers are anonymous, credit should be given to 'Anonymous Referee' and the source.
The images or other third party material in this Peer Review File are included in the article’s Creative Commons license, unless indicated otherwise in a credit line to the material. If material is not included in the article’s Creative Commons license and your intended use is not permitted by statutory regulation or exceeds the permitted use, you will need to obtain permission directly from the copyright holder.
To view a copy of this license, visit https://creativecommons.org/licenses/by/4.0/

HHMI
HOWARD HUGHES MEDICAL INSTITUTE

May 6, 2026

Dear Reviewers,

Thank you for reviewing our manuscript, "**AI-redesigned starting points and outcomes enhance laboratory protein evolution**". In response to your thoughtful and detailed comments, as well as your suggestions, we performed many new experiments and analyses, including:

(1) **Characterization of evolutionary intermediates across starting points and selections**, as requested by Reviewer 3 (Fig. 3a-c), demonstrating that a ProteinMPNN-redesigned starting point can evolve maximal activity faster than the natural counterpart starting point.
(2) **High-throughput, long-read sequencing of phage populations over the course of evolution**, as requested by Reviewer 3 (Fig. 4), where we find that redesign-initiated pools can converge on genotypes that the wild-type pools do not sample, and the redesign-initiated pools can achieve fitness with fewer mutations than the wild-type pools.
(3) **Reversion analysis measuring the expression, stability, and activity contributions of individual mutations from multi-mutant evolved genotypes**, as requested by Reviewer 1, showing how the wild-type starting point can evolve stabilizing mutations to compensate for function-conferring, destabilizing mutations.
(4) **Evolution campaigns of two new, distinct redesigned starting points on the full panel of selection substrates**, in response to questions raised by Reviewer 1 (Fig. 5a-c), showing that a stabilized but catalysis-impaired ProteinMPNN redesign starting point, as well as a PROSS redesign starting point, enhance evolution outcomes.
(5) **Comprehensive assessment of evolved genotype function grafted into all evolutionary starting point backgrounds**, in response to questions from Reviewers 1 and 3 (Fig. 5a-c, Extended Data Fig. 6), finding that redesigns are broadly robust to retain function when combined with mutations that evolved from wild-type or redesigned starting points, while the wild-type protease functions poorly when combined with mutations that evolved from each of the redesigned starting points.
(6) **Analysis of the fitness landscape surveyed by natural and redesigned starting points across evolution campaigns,** advancing our results from (5). We represent the fitness landscape by encoding evolved sequences with a protein language model, which yielded a visualization of protein sequences clustered by evolved function. The analysis helps explain adaptation dynamics observed in (1) and (2) (Fig. 5d), showing that the three alternative redesigns expand and smoothen the fitness landscape, both in the sequence space proximal and distal to the starting point.
(7) **Evaluation of ProteinMPNN score correlations to evolvability measures and activity of evolved variants**, as requested by Reviewer 3 (Extended Data Fig. 7), showing that redesign scores can correlate with, but are not strongly predictive of, evolution outcomes.
(8) **Additional comparisons of the expression and stability of redesigned PTEN-cleaving proteases to the wild-type, unevolved starting point**, as requested by Reviewer 2 (Fig. 2b-c), supporting that ProteinMPNN redesign restores expression and stability lost during new function evolution.

These new experiments and analyses collectively reinforce our conclusion that redesign raises protein evolvability for new function. Further, we extend the stability-evolvability relationship to the new insights that starting point redesign can accelerate adaptation to new function. We find that starting point stabilization confers function to genotypes both closer and farther in mutation space than those that function in the natural protein. Because closer mutants are sampled more rapidly, redesigned starting points can evolve maximal activity faster than the wild-type. Further, stability can raise evolvability for alternative redesign outcomes, including a catalytically impaired ProteinMPNN redesign and a PROSS redesign generated with physics-based modeling.

David R. Liu, Ph.D.
Investigator

Harvard University
12 Oxford Street, Cambridge, MA 02138
Office 617.496.1067 • Fax 617.496.5688 • drliu@fas.harvard.edu

We have also revised the manuscript, figures, and extended/supplementary data to address the reviewers' comments in detail. Point-by-point (in blue) are provided below to each of the reviewers' comments:

Reviewer #1:
In this manuscript, Krasnow et al. present an experimental workflow which integrates computational sequence redesign (ProteinMPNN and benchmarked against PROSS) with continuous evolution (PACE) to achieve functional outcomes that are difficult to access when starting from the natural enzyme. The authors support the premise that natural proteases are constrained by their low stability using thermal stability measurements (melting temperature) and soluble expression yield. The central conceptual claim, that increasing the stability of enzymes via computational redesign enables otherwise-inaccessible genotypes, is well substantiated by parallel evolution, deep sequencing of endpoint genotypes, and systematic grafting of mutations across the wild-type and redesigned backgrounds. Additionally, the therapeutic target ataxin-2 provides a potential application of the protease function and specificity engineering.

Overall, the authors did an excellent job defending their claim. However, I do not find the findings to be surprising. As the authors have acknowledged in Refs 25 and 36, the concept that the 'evolvability' of a protein often correlates with its stability has been established by experimental means over several decades now. The key innovation of this paper is to show that computational redesigned sequences can indeed be used as starting points in PACE, and by doing so, the additional stability enabled access to the destabilizing (yet functionally enhancing) mutations that could not be accommodated by natural proteins, corroborating the abovementioned concept. However, computational redesign to improve protein stability is by no means the only way to yield more stable starting points. Have the authors attempted to use their own solubility-improving PACE circuits to improve the expression of the natural proteases? What about other directed evolution techniques? In other words, the main idea – that stability enhances evolvability – is well known. And, it is also well known that computation can be used to enhance stability. This paper simply takes two well-known concepts and mixes them together. From a utility perspective, this is very exciting and potentially useful. From a conceptual or innovation perspective though, I find this paper does not warrant publication in a journal targeting a very broad audience.

My other major concern is that the experiments were all aimed at confirming the authors' hypotheses, without null testing. For example, one idea, is the authors could include a lower expressing, similar activity starting point or similar expressing, higher activity starting point and compare the evolution outcomes. I suspect these evolutions could yield better performing variants than the WT starting point, which would contradict the authors' claim that higher stability always give better evolution outcomes. This would actually be exciting, as it would show some additional nuance to the literature, which as of now, already directly correlates stability and evolvability. Similarly, the authors could test a series of similarly stabilized redesigned variants, and possibly test if evolvability does not only correlate with stability (perhaps some variant with a lower Tm wins?). Again, this would at least be new knowledge, if it turned out to be true.

We appreciate the reviewer's suggested ideas to augment the novelty and impact of the work. For alternative approaches to generate stabilized starting points such as solubility PACE, we note that retaining function is critical for starting point stabilization and although solubility PACE can simultaneously select for function while evolving expression in the case of protein binders, this selection scheme generally cannot co-select protein function without being vulnerable to a ribosomal reinitiation cheating event in which protein expression uncouples from reporter expression and activation (1). Any high-throughput selection that links soluble expression to reporter activation would in principle suffer the same problem. We have added a statement of this point to the Introduction on page 4.

In response to concern about conceptual advance, we have not yet seen computational protein re-design of natural proteins be adopted by other labs' experimental protein evolution workflows. However, the strategy described in this study has now been implemented in virtually all of our lab's many protein evolution projects because of its major positive impact on outcomes; we are hopeful that other experimental protein evolution researchers will find this approach similarly enabling. To answer the Reviewer's thoughtful questions on how

David R. Liu, Ph.D.
Investigator

Harvard University
12 Oxford Street, Cambridge, MA 02138
Office 617.496.1067 • Fax 617.496.5688 • drliu@fas.harvard.edu

redesigns with distinct properties perform in evolution, we have added new evolution campaigns for two new starting point redesigns evolved on the full panel of selection substrates in replicate. We chose redesign D4, a strongly stabilized ProteinMPNN variant with retained but compromised catalytic activity, and PROSS1, a PROSS redesign with enhanced stability and activity. We find that each of these redesigns evolve to outperform wild-type-initiated evolutions and make functional use of mutation space inaccessible to the wild-type. Surprisingly, D4 evolves the highest activity in multiple cases despite its slower starting kinetics, and D4 evolves a genotype that is functional in no alternative starting point genetic background.

These results add a new and practical nuance that a redesigned starting point can enhance evolution even in cases when redesigned stability trades off with catalytic function. We have dedicated the new **Figure 5** and associated text to these new evolution experiments and the characterization of their outcomes.

Minor issues

1. If the low expression of natural starting point is the limiting factor for accumulating destabilizing mutations, have the authors tried simply increasing the strength of the promoter and RBS or protein solubility tags to boost the expression?

Transgenes are translated from phage with the highly efficient SD7 RBS (2). As with any component of the phage genome, the RBS is subject to elevated mutagenesis and evolution, yet enhanced RBS mutants did not emerge to enhance expression of the wild-type starting point. Solubility tags can impose a fitness penalty by increasing the size of the phage genome and do not confer stability benefit when the protein is applied in settings outside the selection, including therapeutic delivery (3). The latter point is noted in the Introduction on page 4.

2. In PACE, there is also selection pressure for higher solubility/better expression. Can the authors decouple the effect of some mutations boosting expression vs. improving catalytic function?

We have added a new reversion analysis of selected wild-type-evolved and redesign-evolved proteases performed through a series of biochemical assays. We measured the contribution of each individual mutation to expression, thermal stability, and catalytic rate. We observed for the wild-type-evolved clone that mutations that confer function but diminish stability can be rescued by an additional, stabilizing mutation that arises later in PACE. The redesigned enzyme, however, functions as a stable protein without this compensatory mutation, thereby reaching maximal activity with fewer mutations. We describe these results in **Extended Data Fig. 5** and have updated the text accordingly.

3. The authors used ProteinMPNN and benchmarked with PROSS. It would be more convincing if some variants from PROSS are also subjected to PACE and draw similar conclusions.

We agree that an exploration of PROSS redesign evolution would add to the study. In response, we have performed new evolution campaigns on the PROSS-redesigned enzyme described above and in **Figure 5** and in the associated text. We find that the PROSS redesign also evolves higher activity over an expanded functional sequence space and is broadly robust to ProteinMPNN-evolved genotypes.

4. The authors claimed 'specificity' with only comparison to the native substrate SNAP-25. I don't think it's sufficient to claim specificity.

We have modified terminology describing activity for the positive selection substrate (ataxin-2) vs. the negative selection substrate from "specificity" to "selected specificity" to reflect the precise specificity property selected during evolution. To substantiate that enhanced selected specificity reduces off-target effects, we have performed a new toxicity assay in mammalian cells in which the top redesign-evolved protease is less toxic than the wild-type-evolved protease, consistent with higher overall specificity for the redesign-evolved protease mitigating cleavage of cellular proteins. This new result is provided in **Extended Data Fig. 10h.**

David R. Liu, Ph.D.
Investigator

Harvard University
12 Oxford Street, Cambridge, MA 02138
Office 617.496.1067 • Fax 617.496.5688 • drliu@fas.harvard.edu

5. Fig. 1d shows D1-D3 all have higher expression level than WT. Yet, in the PACE evolution campaign for Ataxin-2, D2 evolved variants showed even lower activity than WT-evolved variants for on-target substrate and higher off-target activities (Fig. 4e). How do the authors account for this observation?

Though D2-evolved proteases showed lower on-target activity and similar, near baseline off-target activity for native substrate compared to wild-type evolved variants, we now show in **Supplementary Figure 8** that the D2-evolved proteases are more thermally stable and refer to this new result in the text. The residual stability after the evolution campaign suggests further potential to evolve activity and specificity.

6. In Ext. Data Fig. 6 selection scheme for PANCE1, what does SS1 stand for?

Stepping-stone 1, the intermediate substrate that proteases were evolved to cleave before evolution to cleave ataxin-2, is denoted SS1. The abbreviation is now defined in the associated **Figure 6** caption**, Supplemental Note 3**, and **Supplementary Figure 6**.

7. Page 14, "We predicted a potential ataxin-2 binding register within the BoNT/E binding groove by placing Ile1193 at the P3 position of the protease, since our substrate profile data indicated a strong preference for Ile at this position (Fig. 3a).": The reference to Fig. 3a does not support the claim.

This text, now a part of Supplementary Note 2, has been updated to cite the correct figure **Extended Data Fig. 4a.**

Reviewer #2:
Summary: Krasnow et al. couple deep learning-based sequence redesign with laboratory directed evolution to show that enhancing protein stability of evolutionary starting points expands the accessible sequence space during evolution. Using ProteinMPNN, the authors generated stabilized botulinum neurotoxin (BoNT) proteases and subsequently performed parallel phage-assisted continuous evolution campaigns with the wild-type proteases, selecting for proteolytic activity and/or specificity in two contexts: 1) on similar, yet non-native substrates or 2) on a disease relevant ataxin-2 protein. In all campaigns, sequence-redesigned starting points yielded evolved variants with enhanced activity and specificity (vs. variants derived from the wild-type starting point). Critically, the amino acid substitutions uncovered in the re-designed protease campaigns were largely incompatible with the wild-type background due to protein stability penalties, underscoring the impact of sequence redesign toward evolving otherwise inaccessible variants.

Impact: This study offers a practical strategy for addressing a commonly observed limitation of laboratory evolved proteins: reduced activity and/or specificity for a non-native function compared to the wild-type protein on its native function. While the principle of protein stability conferring mutational robustness (and therefore enhanced evolvability) is well established, generating stable proteins as inputs for evolution experiments has been historically cumbersome (e.g., requiring protein stability selections, modifying the chaperone network, etc.). Sequence redesign with ProteinMPNN appears to be a promising approach to rapidly generate starting points for laboratory evolution, given the observation that proteases evolved from redesigned starting points consistently displayed enhanced activity, solubility, and specificity vs. those evolved from wild-type. Though this study implements existing tools/methods and focuses on a single protein class, the head-to-head comparisons afforded by the parallel campaigns are valuable case studies for the protein engineering community. Furthermore, the evolved redesigned (D3) ataxin-2 proteases themselves are useful biotechnological tools and serve as a proof-of-concept for evolving highly active and specific proteases for cleavage of therapeutically relevant targets. Future work to establish generalizability of the overall strategy to alternative protein targets is greatly anticipated. The work is technically well-done and suitable for publication.

Major Concerns: n/a

We greatly appreciate the Reviewer's support and interest in our work.

David R. Liu, Ph.D.
Investigator

Harvard University
12 Oxford Street, Cambridge, MA 02138
Office 617.496.1067 • Fax 617.496.5688 • drliu@fas.harvard.edu

Minor Comments:

1) Supplementary note 1:
• First paragraph references Fig. S2b, but is describing data displayed in Ext. Data Fig. 3b.
• Second paragraph: References to Fig. S3a-e should be re-labeled to Ext. Data Fig. 3f-k. Additionally, Tm and kcat/KM data for X/D1 and X/D2 appear to be flipped. For example, X/D2 variant is described as more stable, yet has a Tm of 60 vs 68 for X/D1.

We have updated these figure citations and text to accurately describe the data.

2) pp. 9-10, "E(4130)A2 is less stable than wild-type BoNT/E protease (Fig. 2c). We observed that the mutation-grafted D1, D2, and D3 enzymes…express to a higher level in E. coli cells than either BoNT/E or E(4130)A2 (Fig. 2b).": Expression and Tm data for WT BoNT/E are not shown in Fig. 2b and 2c, respectively. These should be added to support the claims in the two sentences listed.

We agree that these comparisons are necessary and have added expression and Tm data to **Fig. 2b-c** in support of our claim.

3) p. 14: Substrate profile data is listed as Fig. 3a, yet is actually shown in Ext. Data Fig. 4.

We have corrected this figure citation to refer to the appropriate figure.

4) Ext. Data Fig. 8a: Blue residues not colored as in Fig. 4.

This note has been removed as coloring is indeed different.

5) Suppl. note 3: Fig. S13 doesn't exist. Rename to Ext. Data Fig. 8a?

This citation has been updated to **Extended Data Fig. 9.**

6) p. 16: First reference to Fig. 5b in the text should be changed to Fig. 5a.

This citation has been updated to the current **Fig. 6a.**

7) p. 17: In paragraph 2, when describing cleavage rate assay, change units of mM to uM.

These units have been corrected.

8) Fig. S3b: D3(428)1 also appears to generate some undesired product (second band from bottom in lane 2), though curiously this is rescued when grafting into E(428)2 (fifth lane). It would be useful to comment on what whether that is a bona fide undesired cleavage byproduct.

We have added a comment to this section that an undesired minor product was observed for the D3-evolved protease, though, there is a greater number and more abundant byproducts formed with E(428)2.

Co-referees #3-5:

Krasnow et al. compare the evolvability of redesigned proteins (using models like ProteinMPNN) with their wild-type counterparts, using BoNT proteases as case studies. The authors show that sequence-redesigned enzyme have greater evolvability, in part mediated by enhanced stability of the starting point enzyme. The authors conduct

David R. Liu, Ph.D.
Investigator

Harvard University
12 Oxford Street, Cambridge, MA 02138
Office 617.496.1067 • Fax 617.496.5688 • drliu@fas.harvard.edu

substantial experimental work including continuous evolution experiments that help to concretely establish claims around evolvability, as well as thorough biochemical and functional assays to characterize the evolved variants.

This paper excels through the strength of its experimental data, especially in the ability to sample many evolutionary trajectories in laboratory settings, as well as a breadth of orthogonal readouts supporting the conclusions (including soluble expression, Tm, activity and kinetics, luciferase circuit fitness, sequencing, mammalian cell assays, gel shift, MS, and MD). However, the paper has much more limited conceptual novelty; it is well established dogma in the directed evolution field that stability promotes evolvability (Bloom et al., PNAS, 2006), which this paper does not overturn, and the methods the authors use (in particular, ProteinMPNN) have already been shown to improve directed evolution in lower throughput settings (e.g., Sumida et al., 2024).

Our view of this paper is therefore as a borderline case and for which we would request responses to our questions below, especially regarding the conceptual novelty of this work, before we can make a decision on whether to recommend publication of the paper in Nature.

We thank the Reviewers for their interest in our work and for their suggestions to highlight and strengthen the novelty of our findings.

Major comments:

• ProteinMPNN is already well known to rescue or improve protein expression, stability, and functon(e.g., Sumida et al., 2024, as cited by the authors). In addition, as the authors note, prior work has shown that ProteinMPNN can increase the thermal stability of wild-type enzymes through a systematic pipeline, and that sequence-redesigned enzymes can be evolved to improved activity for a non-native remote C(sp3)-H hydroxylation reaction (King et al., 2025). Given this context, it is critical for the manuscript to more clearly articulate the unique value and new insights enabled specifically by the present study.

We have edited the abstract, introduction, and conclusion to more explicitly state insights our original submission contributes to the stability-evolvability literature:

- Systematic evolved genotype grafting reveals that mutations that function within the wild-type background are a subset of those that function within a stability-redesigned background.
- Redesign can enhance the outcome of specificity evolution away from the native substrate in favor of a nonnative substrate, even when redesigns are identified for retained or elevated native substrate activity.

The experimental investigations suggested by these and the other Reviewers, yielded two new conceptual findings that we have added to this revised submission:

- Redesigned starting points can adapt faster to new function. Redesigned starting points can enable new activity with genotypes that are closer in sequence to the starting point than those genotypes that allow the wild-type starting point to achieve similar levels of new activity. Closer mutants are accessed earlier in evolution than distant mutants, explaining how the redesign can evolve maximal activity faster than the wild-type. We present the results in support of this claim in PACE intermediate characterization in **Fig. 3**, genotype distribution dynamics in **Fig. 4**, reversion analysis in **Extended Data Fig. 5**, and fitness landscape analysis **Fig. 5d**. We added these findings to the revised text.
- Alternative redesigned starting points, now including a highly stabilized but catalytically impaired ProteinMPNN redesign as well as a PROSS redesign, each raise activity across the fitness landscape and expand the landscape to new functional sequences. across selections. A highly stable yet kinetically slow redesign can reach superior performance during evolution compared to wild-type and alternative redesigns. These additional evolutions and the comprehensive assessment of their outcomes grafted into all starting genetic backgrounds are described in **Figure 5, Extended Data Fig. 6** and related text.

• It would be especially valuable to summarize grafting asymmetry quantitatively and experimentally across campaigns. This could be done by comparing the fraction of wild type-evolved mutation sets that remain functional in D3, versus the fraction of D3 evolved mutation sets that remain functional in wild type, ideally alongside activity and stability effect sizes. This appears likely to be achievable from data already generated and would sharpen the

David R. Liu, Ph.D.
Investigator

Harvard University
12 Oxford Street, Cambridge, MA 02138
Office 617.496.1067 • Fax 617.496.5688 • drliu@fas.harvard.edu

robustness claim considerably, though the authors should regenerate new data as appropriate to answer this question.

Having now evolved three redesigns and the wild-type protease on the panel of three substrates, we report analyses of the activity of each consensus genotype in all four starting point backgrounds (**Fig. 5a-c**). We also calculate the fraction of genotypes that are functional in each background according to their origin background in **Extended Data Fig. 6c-e.** Correlating evolved genotype activity in each redesign to activity in the wild-type shows a poor association, further reinforcing that redesigns confer strong activity to genotypes that perform poorly in a wild-type genetic background.

• An analysis that could add substantial value to the community is a comparison of ProteinMPNN sequence scores or likelihoods for PACE-evolved variants, versus the initial seed(s). This would help clarify the division of labor between model-guided redesign and evolutionary search, and could show whether redesign alone would be likely to generate variants in the same fitness region (which is unlikely). Relatedly, Ext. Data Fig. 1 already provides a useful in silico evaluation of redesigned seeds (AF2 confidence, RMSD, and sequence divergence), but the manuscript is also in a strong position to provide a post hoc benchmark of redesign metrics against empirical outcomes. Correlation plots between AF2-derived metrics (for example pLDDT, iPAE, RMSD, or designability type metrics) and experimental outcomes such as soluble expression, Tm, activity retention, or later evolvability would be very informative, even if none show any signal. In particular, it would be valuable to test whether any computational metric has a signal for predicting good starting points with D3 like behavior.

We agree, and in response have performed new analyses to determine whether Protein MPNN scores predict properties related to evolvability or the fitness of evolved genotypes (now presented in **Extended Data Fig. 7**). We show that ProteinMPNN scores correlate poorly with soluble expression across the panel of assayed redesigns, but scores do correlate with Tm measured for the variants used as starting points. We also find that high ProteinMPNN scores (low likelihood) are weakly predictive of low fitness when calculated for the mutated residues for the protease-substrate complex structure. We have updated the text accordingly.

• Relatedly, a residue-level analysis of where MPNN-introduced mutations cluster (ex: surface versus core, secondary structure context, distance to active site, or packing networks) would also strengthen the mechanistic interpretation, even if descriptive only. The distance and conservation cutoff choices seem reasonable and the empirical comparisons are useful, but the cutoffs still read somewhat heuristic. A brief, more explicit justification of these cutoffs would help, especially around the tradeoff between preserving catalytic and substrate-recognition determinants and allowing enough redesign freedom to improve stability and robustness. A small table summarizing the number of frozen versus redesignable residues for each ProteinMPNN setting and serotype would also make the design strategy easier to evaluate and reproduce.

In response, we have added new analyses of the structural features and residue property transitions for redesign mutations in **Supplementary Figure 2**, and the conservation of redesign mutations defined by frequency observed in an MSA are now included in **Supplementary Figure 3.** These redesign statistics are quantified in Supplementary Table 4 and observations summarized in the text. To help justify the choice of distance constraints, we add a note to the text acknowledging that "distance constraints, though heuristic, were set intending to preserve the structural dynamics that contribute to function in BoNT proteases" with an added citation where those dynamics are observed (4).

• Finally, much of the current evidence focuses and the start and endpoints of evolution, with the grafting experiments carrying much of the mechanistic interpretation. The manuscript could be further strengthened by a more explicit trajectory level comparison of wild-type versus redesigned backgrounds across replicates. For example, presenting forms of "adaptation dynamics" such as i) time to stable propagation, ii) time to a predefined activity threshold, or iii) success frequency under matched stringency schedules. If intermediate timepoint samples are available, a simple diversity over time analysis (for example high abundance genotype counts, mutational

David R. Liu, Ph.D.
Investigator

Harvard University
12 Oxford Street, Cambridge, MA 02138
Office 617.496.1067 • Fax 617.496.5688 • drliu@fas.harvard.edu

distance from seed, or population entropy) would provide direct support for the accessible sequence space and evolvability claims.

We thank the Reviewers for their thoughtful recommendation to explore how adaptation dynamics vary between redesigned and wild-type starting points, which led to striking observations. In response, we performed new experiments measuring activity of consensus genotypes from intermediate timepoints across the panel of selection substrates, and we observed that the redesign-evolved clones are consistently more active than wild-type-evolved clones over the timepoints assayed. For two of the three selections, the redesign reaches maximal activity faster than the wild-type (**Fig 3a-c, bottom**). These data and our new results presented in **Fig. 4**, **Extended Data Fig. 5**, and **Fig. 5d** support a model in which redesign accelerates adaptation when the expanded functional sequence space of redesigned starting points includes solutions more local to the starting point that are rapidly sampled in evolution.

The suggested summary statistics for adaptation observed in PACE titers are now summarized in **Supplementary Table 6**. To deeply profile the mutation distributions, diversity, and mutational distance over these PACE experiments, we have added new high-throughput, long-read sequencing data of population genotypes over the course of multiple replicates each of the redesigned and wild-type starting points for one evolution. These data make up the new **Figure 4**. Surprisingly, the redesign-initiated populations sampled single mutants more frequently and had lower average Hamming distance than wild-type-initiated populations early in evolution, but redesigns sampled more multi-mutant genotypes not observed in wild-type-initiated populations as time and flow rate progressed. These data support that early sampling of local mutations underlies rapid adaptation, which is further supported by fitness landscape analysis in **Figure 5d** revealing that redesign raises the fitness of genotypes proximal to the starting point. We have added these findings to the text accordingly.

Minor comments:

• Protein expression levels in Figs. 1d and 2b should be quantified.

The text for these sections now notes the fold improvements to soluble yield that each redesign confers.

• In Ext. Data Fig. 2e, the kinetic measurements appear to differ substantially from those in Fig. 1f (for WT, D1, D2, and D3). The authors should explain the source of these differences.

The two sets of data were each generated from an independent round of protein purifications, and protein quality may vary between purification rounds. We rely on data from proteins purified in the same round for head-to-head comparisons.

• Similarly, the scatter in Ext. Data Fig. 2b for D1–D3 appears different from the scatter plot in Fig. 1c. The authors should explain the discrepancy.

Because the data in Fig. 1c were generated in singleton we do not know the variability of the data or whether the different results for D1-D3 in Ext. Data Fig. 2b are within error of the first experiment. We expect that this small scale-parallelized purification experiment is effective for identifying redesign hits with improved properties while lower throughput, higher yielding purification and assays that we follow these experiments with best quantify stability and catalytic measurements.

• Fig. 5g would benefit from quantification, including on-target/off-target fold changes across variants and direct comparisons of these fold changes. From the bar plots, the on/off ratio appears similar between D3(428)2 and E(428)4 evolved from the wild-type BoNT protease.

We have added the following note for the text describing the now **Fig. 6i:**
"Though D3(428)2 and E(428)4 showed similar selected specificity in this experiment (30.0-fold vs. 30.2-fold), this is likely because both proteases cleave SNAP-25 with activity below the detection limit of this assay"

David R. Liu, Ph.D.
Investigator

Harvard University
12 Oxford Street, Cambridge, MA 02138
Office 617.496.1067 • Fax 617.496.5688 • drliu@fas.harvard.edu

We appreciate the time and effort you dedicated to evaluating our work. The revised manuscript has been substantially improved by the inclusion of our new experimental data arising from your feedback, and by the resulting new conceptual insights. We are hopeful these additions resolve the prior concerns and make a compelling case for publication in *Nature*. Thank you very much for your assistance; I look forward to your reply.

Sincerely yours,

David R. Liu
Thomas Dudley Cabot Professor of the Natural Sciences and
Professor of Chemistry and Chemical Biology,
Harvard University
Vice-Chair of the Faculty and Core Institute Member,
Broad Institute of Harvard and MIT
Howard Hughes Medical Institute Investigator

David R. Liu, Ph.D.
Investigator

Harvard University
12 Oxford Street, Cambridge, MA 02138
Office 617.496.1067 • Fax 617.496.5688 • drliu@fas.harvard.edu

References

(1) Wang, T., Badran, A. H., Huang, T. P. & Liu, D. R. Continuous directed evolution of proteins with improved soluble expression. *Nat. Chem. Biol.* **14**, 972–980 (2018).
(2) Ringquist, S., Shinedling, S., Barrick, D., Green, L., Binkley, J., Stormo, G. D. & Gold, L. Translation initiation in *Escherichia coli*: sequences within the ribosome-binding site. *Mol. Microbiol.* **6**, 1219–1229 (1992).
(3) Tokuriki, N. & Tawfik, D. S. Chaperonin overexpression promotes genetic variation and enzyme evolution. *Nature* **459**, 668–673 (2009).
(4) Breidenbach, M. A. & Brunger, A. T. Substrate recognition strategy for botulinum neurotoxin serotype A. *Nature 2005 432:7019* **432**, 925–929 (2004).

David R. Liu, Ph.D.
Investigator

Harvard University
12 Oxford Street, Cambridge, MA 02138
Office 617.496.1067 • Fax 617.496.5688 • drliu@fas.harvard.edu
